# Improved protein binder design using β-pairing targeted RFdiffusion

**DOI:** 10.1038/s41467-025-67866-3

**Published:** 2026-01-10

**Authors:** Isaac Sappington, Martin Toul, David S. Lee, Stephanie A. Robinson, Inna Goreshnik, Clara McCurdy, Tung Ching Chan, Nic Buchholz, Buwei Huang, Dionne Vafeados, Mariana Garcia-Sanchez, Nicole Roullier, Matthias Glögl, Christopher J. Kim, Joseph L. Watson, Susana Vázquez Torres, Koen H. G. Verschueren, Kenneth Verstraete, Cynthia S. Hinck, Melisa Benard-Valle, Brian Coventry, Jeremiah Nelson Sims, Green Ahn, Xinru Wang, Andrew P. Hinck, Timothy P. Jenkins, Hannele Ruohola-Baker, Steven M. Banik, Savvas N. Savvides, David Baker

**Affiliations:** 1 Department of Biochemistry, University of Washington, Seattle, WA USA; 2 Institute for Protein Design, University of Washington, Seattle, WA USA; 3 Graduate Program in Biological Physics, Structure and Design, University of Washington, Seattle, WA USA; 4 Unit for Structural Biology, Department of Biochemistry and Microbiology, Ghent University, Ghent, Belgium; 5 Unit for Structural Biology, VIB Center for Inflammation Research, Ghent, Belgium; 6 Department of Genome Sciences, University of Washington, Seattle, WA USA; 7 Department of Chemistry, Stanford University, Stanford, CA USA; 8 Howard Hughes Medical Institute, University of Washington, Seattle, WA USA; 9 Institute for Stem Cell and Regenerative Medicine, University of Washington, Seattle, WA USA; 10 Department of Bioengineering, University of Washington, Seattle, WA USA; 11 Department of Chemistry, University of Minnesota, Minneapolis, MN USA; 12 Department of Structural Biology, University of Pittsburgh School of Medicine, Pittsburgh, PA USA; 13 Department of Biotechnology and Biomedicine, Technical University of Denmark, Kongens, Lyngby Denmark; 14 Molecular & Cellular Biology, Medical Scientist Training Program, University of Washington, Seattle, WA USA; 15 Sarafan ChEM-H, Stanford University, Stanford, CA USA

**Keywords:** Protein design, X-ray crystallography, Kinetics

## Abstract

Designing proteins that bind with high affinity to hydrophilic protein target sites remains a challenging problem. Here we show that RFdiffusion can be conditioned to generate protein scaffolds that form geometrically matched extended β-sheets with target protein edge β-strands in which polar groups on the target are complemented with hydrogen bonding groups on the design. We use this approach to design binders against edge-strand target sites on KIT, PDGFRɑ, ALK-2, ALK-3, FCRL5, NRP1, and α-CTX, and obtain higher (pM to mid nM) affinities and success rates than unconditioned RFdiffusion. Despite sharing β-strand interactions, designs have high specificity, reflecting the precise customization of interacting β-strand geometry and additional designed binder-target interactions. A binder-KIT co-crystal structure is nearly identical to the design model, confirming the accuracy of the design approach. The ability to robustly generate binders to the hydrophilic interaction surfaces of exposed β-strands considerably increases the range of computational binder design.

## Abstract (web summary)

This study demonstrates the capability of deep learning protein design models in generating functionally validated β-strand pairing interfaces, expanding the structural diversity of de novo binding proteins and accessible target surfaces.

## Introduction

There has been considerable recent progress in de novo protein binder design^1–6^. Both energy based^1^ and deep learning methods, such as RFdiffusion^2^, now enable design of protein binders given only the structure of the target of interest and (optionally) the specification of region of the target surface to bind^3^. Despite this progress, design of high affinity binders to hydrophilic regions of a target surface remains challenging since the exposed hydrogen bond donors and acceptors must be complemented with precisely positioned acceptors and donors on the designed binder to compensate for the loss of interactions with water. While RFdiffusion excels at generating backbones that are shape complementary to the targeted region of a protein surface, these solutions do not always provide detailed complementarity of hydrogen bonding donors and acceptors^7^. Many therapeutically relevant target proteins have β-sheets with unpaired and exposed β-strands; these often have non-canonical structures to reduce the tendency for aggregation^8,9^. β-strand targeted binders have been generated using pre-deep learning Rosetta methodology^10^, but this approach has limited ability to match the diversity of edge β-strand geometries. A generalizable deep learning method for designing β-strand pairing based binders to complement arbitrary target β-strand twists^11^, bends^12^, bulges^13^, and other irregular features^14,15^ could improve the design of binders to polar target surfaces.

Here, we demonstrate that RFdiffusion generation of binder backbone mediated hydrogen bonding interfaces with the target can yield designs with polar groups nearly perfectly complementing those on the target. We develop a general approach for guiding RFdiffusion denoising-diffusion trajectories towards such β-strand centric interfaces, and we experimentally validate this binder design approach.

### β-strand interface conditioning improves binder designs

**Fig. 1:**
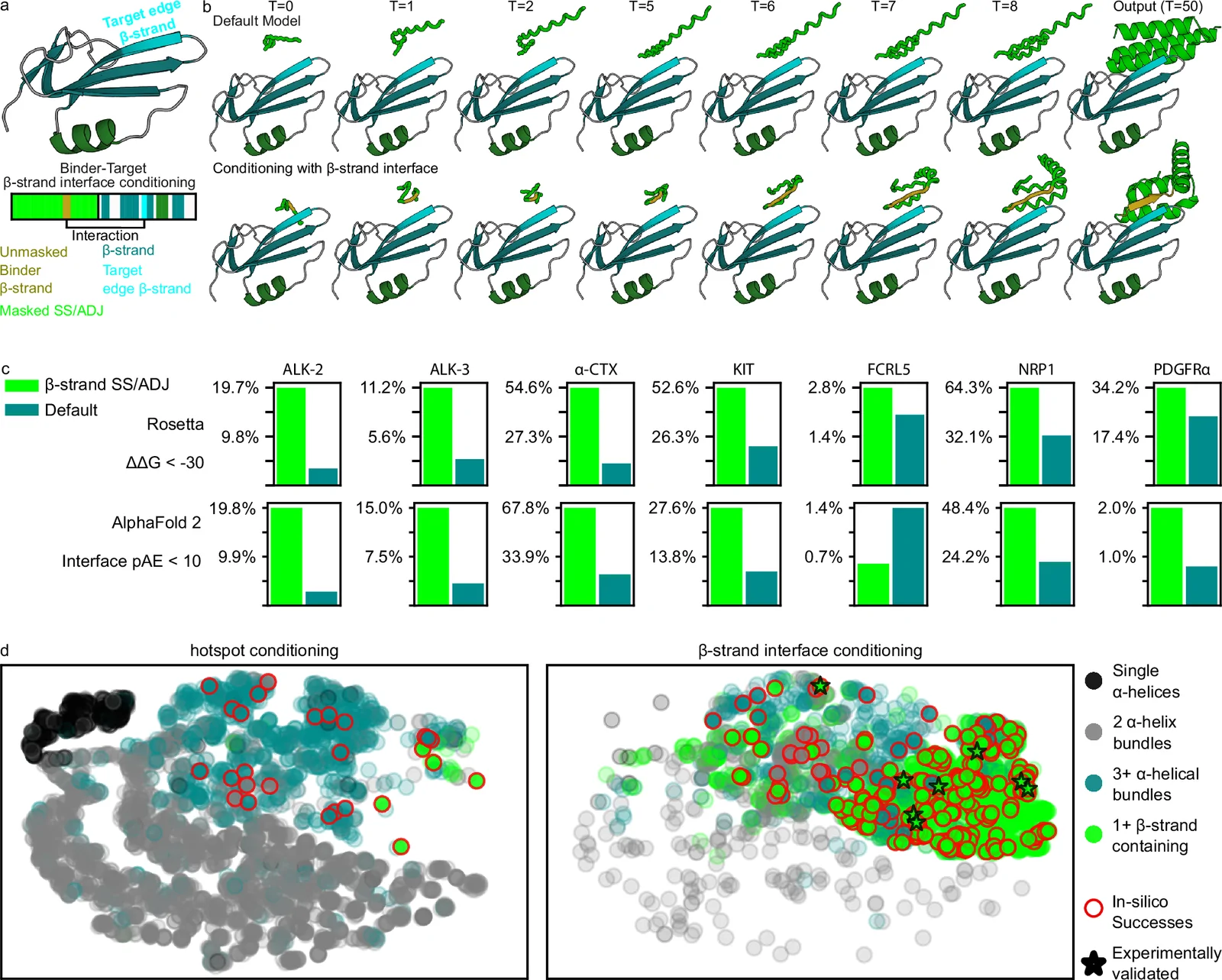
Design of β-strand pairing binders. **a** Representation of β-strand interface conditioning information provided as a tensor to RFdiffusion to generate β-strand pairing binders. **b** Example RFdiffusion denoising trajectories without (top) and with (bottom) β-strand interface conditioning. Conditioning indicates that part of the binder scaffold should be a β-strand (gold) that contacts the indicated target edge strand (cyan). This information influences the denoising in very early trajectory timesteps (t), with the tertiary fold determined within 5 timesteps and the final output at *t* = 50. **c** Binder design success rates using β-strand interface conditioning or RFdiffusion default settings with hotspots indicating the target edge strand of interest. **d** Structural clustering of RFdiffusion output binder scaffolds using hotspot conditioning (left) or β-strand interface conditioning (right) t-distributed Stochastic Neighbor Embedding (t-SNE) transforms of all-by-all pairwise template modeling (TM) scores^60^ among binder scaffolds across all targets are plotted, with close proximity of points representing structural similarity. Output fold secondary structures are classified by color as indicated in the legend. Bold bordered data points indicate in silico successes (red circles, pAE interaction <10, pLDDT >85, ΔΔG < −30) and experimentally validated binders (black stars). Source data are provided as a Source Data file.

During training of RFdiffusion, a subset of training examples was provided with secondary structure and secondary structure block-adjacency (SS/ADJ) information; this was found to enable the conditioning of the model towards user defined protein monomer folds (as previously described by Watson et. al.)^2^. We explored whether providing interface conditioning information indicating a binder β-strand pairing with a target edge β-strand at the point of inference could yield designable strand pairing complexes. RFdiffusion takes as input desired residue secondary structures as well as an N by N block-adjacency matrix (N is the length of the designed protein), which specifies desired adjacencies between secondary structure blocks. These conditioning tensors can specify binder residues having desired secondary structure identities (helix, strand, loop, or masked) as well as the binder residue interaction identities (interacting, non-interacting, masked) with other binder residues (used for fold conditioning), or with target residues (used here for interface conditioning). Given the length of the designed interacting β-strand (L) and the identity of the target β-strand to be bound (T), RFdiffusion generates binders assigning a random set of L consecutive binder residues to form a β-strand pair with the target strand T, while the remainder of the binder output residues are not explicitly assigned a secondary structure or target interaction. We found that outputs from conditioned β-strand pairing runs indeed contained the user specified β-strand interfaces (Fig. 1a-b, Supplementary Fig. 1).

We supplied RFdiffusion with β-strand interface conditioning tensors for larger scale binder design campaigns against protein targets containing edge β-strands sufficiently exposed for binder access. To evaluate the generality of the approach, we selected targets that span a range of edge β-strand geometries. The seven selected targets also have considerable therapeutic relevance. Activin receptor-like kinases 2 and 3 (ALK-2 and ALK-3) are both Type I bone morphogenetic protein receptors that regulate growth of bone, vasculature, hair follicles, enamel as well as wound healing and tumor suppression in various soft tissues^16–20^. Platelet-derived growth factor receptor ɑ (PDGFRɑ) and Mast/stem-cell factor receptor (also known as KIT, SCFR, CD117) are both type III receptor tyrosine kinases that play roles in cardiomyocyte proliferation and heart tissue regeneration after myocardial infarction^21–29^. FCRL5 functions in critical signaling pathways for B-cell activation^30^. NRP1 is a coreceptor for various growth factor signaling pathways (TGF-1, EGF, VEGF, PI3K, HGF, and PDGF)^31^, a viral entry factor^32^, and plays a role in the of RAS/MAPK signaling in various cancers^33,34^. α-Cobratoxin (α-CTX), the lone non-receptor target, is a prominent toxin derived from elapid snakes^35^ that acts by blocking muscle and nerve acetylcholine signaling. All except α-CTX are therapeutic targets for different cancers^36–48^. Designed protein binders against these targets could be useful for antagonizing native signaling, targeting drug conjugates and other therapeutics to tumors, designing novel agonists^49–51^ and targeted receptor degradation^52^, inhibitors^1^, triggering cargo endocytosis^52^, or target therapeutics towards particular cell types^53^.

The set includes single β-sheet targets with highly exposed edge β-strands (α-CTX, FCRL5, NRP1) and somewhat occluded edge β-strands (ALK-2 and ALK-3), as well as immunoglobulin (Ig) fold β-sandwich domains (FCRL5, KIT, and PDGFRɑ). These were found to be challenging for binder design using standard hotspot-only conditioned RFdiffusion–either in silico design calculations had failed to generate designs predicted with high confidence to bind to the target, or experimental testing had failed to yield binders (ALK-3, KIT, see Data Availability). Because no experimentally determined structure exists for FCRL5, we designed binders against the AlphaFold 2 model^54–56^. To provide a stringent test of design specificity, the set includes some structurally related targets: ALK-2 and ALK-3 are members of the TGF-β superfamily^57^, and KIT and PDGFRɑ are type-III receptor-tyrosine kinases^58^ (Supplementary Fig. 2).

In a first in silico benchmark experiment, we compared the diversity and quality of binders designed using our interface conditioning method to designs generated using the standard RFdiffusion target site “hotspot” directed approach. Following the generation of 500 scaffolds using each diffusion approach, ten candidate binder sequences were generated for each scaffold by ProteinMPNN^59^ and each binder candidate complex was predicted with AlphaFold 2^54^. Across all eight target sites across the seven target proteins (two sites were targeted for PDGFRɑ due to predicted structural variability), β-strand interface conditioning yielded design models with improved in silico binding metrics^6^ AlphaFold 2 interface predicted aligned error (pAE) and Rosetta ΔΔG (Fig. 1b), with over 9.2% of β-strand interface conditioned designs meeting reasonable quality metrics, in contrast to 0.98% success rates by RFdiffusion conditioned on target hotspots alone. β-strand interface conditioning yielded in silico design successes for all targets, whereas hotspot conditioning alone did not yield any in silico successes for two targets (ALK-3 and FCRL5). β-strand interface conditioning also yielded more globular binder designs with 88.7% of output scaffolds meeting radius of gyration criteria and only 25.5% of designs from the other methods meeting the same criteria (Supplementary Fig. 3). β-strand interface conditioning yielded outputs that covered a distinct protein structure space compared to other methods, as quantified by all-by-all pairwise template modeling (TM) scores^60^ between aligned binder scaffolds (Fig. 1c), with as expected a higher fraction of β-sheet containing binders and a decrease in ɑ-helical bundle outputs (Fig. 1d).

**Fig. 2:**
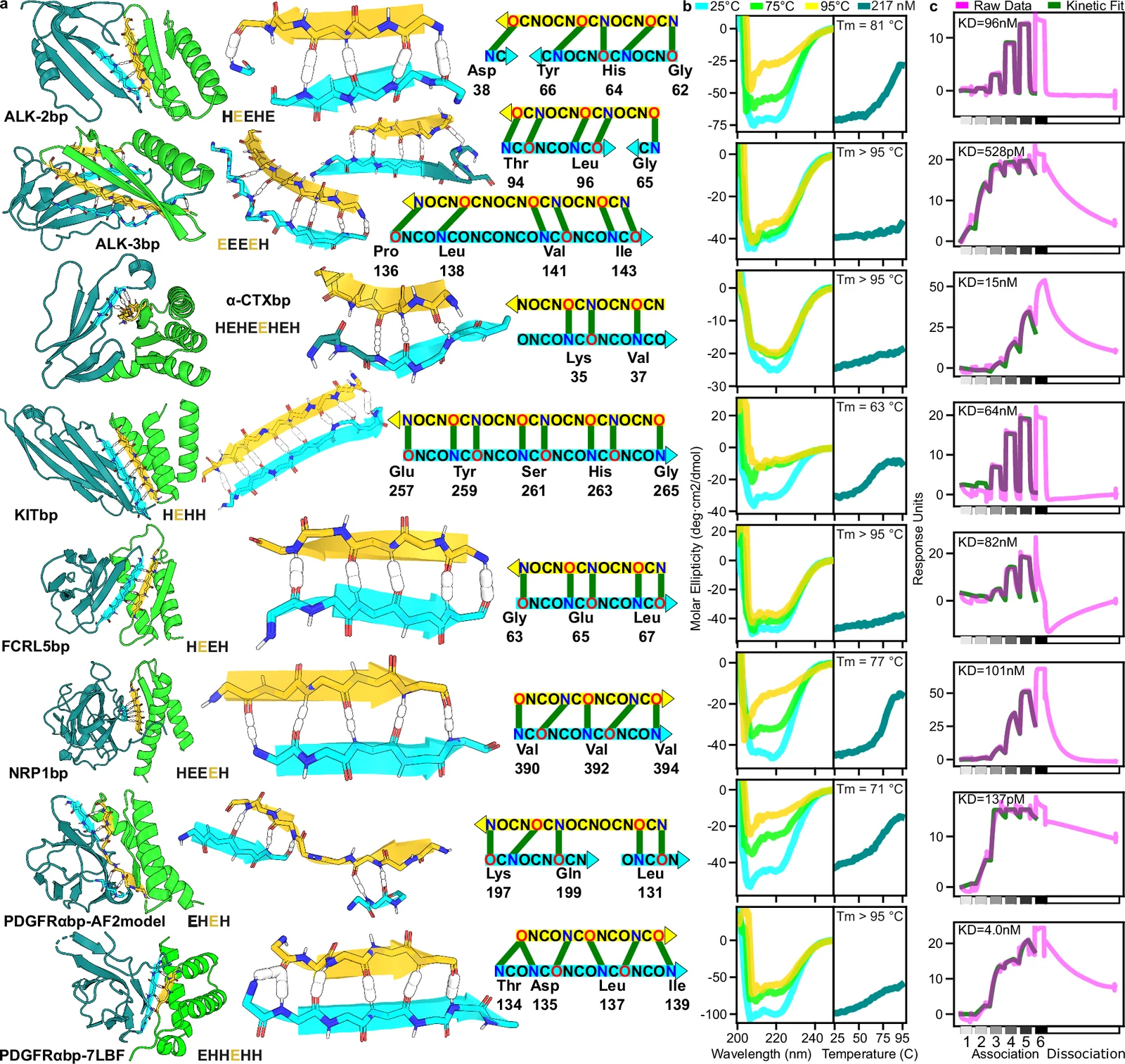
Binder design models and biophysical characterization. **a** Design models of binding complexes for each target. Left: Entire complex design models with binders indicated in green/gold and targets in teal/cyan. β-strand interface conditioned scaffold design yielded binders with interfacial β-strands (gold) forming strand-pairing hydrogen bonding interactions with target edge β-strands (cyan). Middle: Close up view of design model backbone hydrogen-bond interactions with putative hydrogen bonds shown in white. Right: schematic representation of strand pairing interactions showcase the diversity of sequence-independent β-strand pairing interactions. **b** Circular dichroism thermal melts. Full spectrum analyses (left) performed at 25 °C (cyan), 75 °C (green), and 95 °C (gold) assess the overall binder fold at these three temperatures, while single wavelength thermal melts (right) were measured at 217 nM to calculate binder Tm values. **c** SPR measurement (pink) of binding kinetics at 600pM, 4 nM, 30 nM, 200 nM, 1.5 μM, and 10 μM (association phases 1-6 on the X-axis). Fits for *K*_d_ determination (green) excluded the 10 μM data excluded due to signal aberrations at this high binder concentration. Binders were reproduced and similar SPR kinetics were fitted with *n* ≥ 2 for each binding protein. Source data are provided as a Source Data file.

A second, much larger campaign was carried out for each target to generate designs for experimental testing. The sets were generated using β-strand targeted RFdiffusion and with standard hotspot conditioned RFdiffusion, followed by ProteinMPNN, and selection based on AlphaFold 2 and Rosetta metrics; partial diffusion scaffold optimization^4,61^ and additional sequence sampling was carried out to generate designs with improved metrics in several cases. β-strand pairing designs greatly outperformed other scaffold types in this in silico filtering stage for most targets with the exception of the PDGFRɑ target site, which was likely more amenable to helical bundle binder design success due to its flat, hydrophobic surface (Supplementary Fig. 2b). Structurally diverse designs with high AlphaFold 2 confidence (interface pAE <8 and pLDDT > 85) designs were selected for experimental screening by yeast surface display. While we ordered libraries including thousands of designs for some targets (KIT, PDGFRɑ, FCRL5, ALK-2, and ALK-3), binder success rates in our screen suggested that it would be possible to design strand pairing binders at smaller scales (Supplementary Table 1). Indeed, design sets for NRP1 and α-CTX yielded binders at 96 well scales. The α-CTX binder originally introduced by Vazques-Torres et. al., referred to in that work as LNG, will be referenced as α-CTX binding protein (α-CTXbp) in this work. α-CTXbp had been designed with the interface-conditioning method introduced here, but limited details on the method were revealed previously^62^. For PDGFRɑ, where both helical bundle designs and β-strand pairing designs passed in silico filters, significantly more binders were obtained with the strand directed approach than with standard RFdiffusion at this selection stage (Supplementary Fig. 4). The most enriched designs for binding affinity and expression from this assay were cloned, expressed in *E. coli*, and purified (Supplementary Fig. 5). SPR revealed mid- to sub-nanomolar affinity binders for our targets (Fig. 2a-c). In the following sections, we describe the active designs for each target in turn.

### TGF-β superfamily targets: ALK-2 and ALK-3

ALK-2 and ALK-3 are two of four known Type I Bone Morphogenetic Protein Receptors (BMPRI). Targeting BMPR therapeutics towards specific tissues and pathologies could have considerable utility, but achieving binding specificity for ALK-2 versus ALK-3 presents a difficult challenge due to their structural homology. The ALK-2 and ALK-3 ectodomains have 30% sequence identity and high structural similarity (Supplementary Fig. 2). On both targets, there is an edge β-strand with five consecutive residues with backbone atoms in very similar atomic positioning (RMSD 0.07), and each strand is partially occluded by an N-terminal coil. Prior to this study, multiple design campaigns—including campaigns utilizing RFdiffusion, ProteinMPNN, and AlphaFold 2—had failed to yield binders for ALK-3 (see Data Availability). The difficulty of designing binders to these targets is demonstrated in their lower success rates during our yeast surface display assay, with less than 1% of designs showing strong binding and enrichment (Supplementary Table 1).

We conditioned RFdiffusion on generating β-strand pairings to these five edge β-strand residues that were not occluded by the N-terminal coil. We identified binding proteins, ALK-2bp and ALK-3bp, with *K*_d_ values of 96 nM and 528 pM, respectively (Fig. 2c) by surface plasmon resonance (SPR). While both binders were intended to strand pair with the homologous target edge β-strand, the designed interaction footprints of their validated were quite distinct, with ALK-2bp forming a parallel β-strand interaction with the target β-strand and ALK-3bp pairing to the same strand in an antiparallel manner. ALK-3bp makes additional strand-pairing like contacts with the C-terminal coil of ALK-3 in a non-canonical hydrogen bonding pattern, with the binder β-strand curvature nearly perfectly complementing a bulge in the coil motif; these additional interactions may account for the four orders of magnitude greater binding affinity. This additional hydrogen bonding network with the C-terminal coil was not prespecified; RFdiffusion simply generated the secondary binder β-strand given the context of the initial β-strand motif specified by interface conditioning.

### Single exposed edge β-strand: α-CTX, FCRL5, and NRP1

Three of the targets have more exposed edge β-strands. The acetylcholine receptor antagonist α-CTX is a three-finger toxin that consists of a single β-sheet with extended loops that bind a hydrophobic receptor pocket. We aimed to design a β-strand pairing binder that would sterically interfere with this interaction. FCRL5 weakly binds IgG to modulate B-cell activation. While the exact IgG binding site is unknown^63^, the receptor consists of several Ig domains. In the AlphaFold 2 model, the N-terminal Ig domain contains one three stranded sheet and one five stranded sheet, leaving an exposed β-strand available to target during design. Finally, we targeted the discoidin domain of NRP1, which contains a highly twisted β-sheet with a potential β-strand binding site where the edge β-strand twists about 90 degrees with exposed backbone polar atoms before entering into the protein core. While quite exposed, these target edge strands have distinctive structures that we hypothesized could allow for specific β-strand complementarity.

β-strand interface conditioned RFdiffusion resulted in a 5-fold improved in silico success rate for these targets compared to hotspot directed RFdiffusion which, even without conditioning, generated β-strand pairing binder designs with up to 20% of outputs (β-strand pairing designs were generated <5% of the time for the less exposed edge strand containing targets). Binders for α-CTX (originally introduced by Vazques-Torres, et. al.) and NRP1 (1.9 nM and 101 nM, respectively) were obtained by testing the 96 designs based solely on in silico metrics. The best binder to FCRL5, with a 82 nM Kd, was obtained from a yeast display library of 4841 designs. The FCLR5 and NRP1 target β-strands both had similar conformational twists, but FCLR5bp and NRP1bp utilize quite distinct antiparallel and parallel hydrogen bonding binding modes, respectively (Fig. 2a, right). Similar to FCLR5bp, α-CTXbp forms a mostly canonical antiparallel strand pair with a slight irregularity as the binder β-strand adapts to complement a small target strand bulge.

### Type III receptor tyrosine kinases: KIT and PDGFRɑ

The type III RTK family receptors KIT and PDGFRɑ play roles in angiogenesis, tissue regeneration, and aberrant cancer signaling. Both receptor ectodomains are comprised of five Ig-like folds, with native ligands—stem cell factor and platelet derived growth factor—activating cellular signaling pathways by binding Ig domain 2 of KIT and PDGFRɑ, respectively, to induce receptor dimerization and intracellular cross-phosphorylation. Binders designed to occupy the ligand-binding pocket could act as antagonists to prevent aberrant signaling, and when oligomerized^50^, high-affinity binders could function as strong signaling agonists for tissue repair therapeutics. As such, we targeted the ligand binding sites on domain 2 of each receptor. As in the case of ALK-3, previous attempts to de novo design binders against KIT had failed (see Data Availability).

For KIT, a 65 nM binder (KITbp) was identified from a yeast surface library screening of 1298 designs. KITbp was designed to bind domain 3 strand on KIT which is part of the stem cell factor ligand binding site; the binding interface features an extensive canonical antiparallel hydrogen bond network, with 8 consecutive hydrogen bonds contributing a β-strand pair of 18 total binder and target residues (Fig. 2a, left). For PDGFRɑ, two binders were identified—PDGFRɑbp-7LBF (*K*_d_ = 137 pM) was designed to bind the cryo-EM structure (PDB accession code: 7LBF), while PDGFRɑbp-AF2model (*K*_d_, 4 nM) was designed to bind the AlphaFold 2 model. They were enriched from 5427 and 189 member design libraries via yeast display, without any further experimental sequence optimization. Despite significant disagreement between the cryo-EM structure and the AlphaFold 2 model regarding the conformation of the target domain 2 Ig fold, design against both conformations yielded high-affinity binders. The binders may selectively induce the cognate PDGFRɑ conformation upon binding, as their design model conformations are not cross-compatible (Supplementary Fig. 7). PDGFRɑbp-7LBF forms a complex h-bond network in which the binder strand forms both parallel and antiparallel interactions with two different target strands of the target domain 2 Ig fold. This precisely complementary strand pairing h-bond network highlights the power of β-pairing conditioned RFdiffusion to design complex β-strand architectures to perfectly complement idiosyncratic target topologies.

### Stability of binders with exposed edge β-strand interfaces

No obvious trends were observed between the size of the edge β-strand interface and aggregation propensity of the binders. All binders could be purified at high yields, and analysis of size exclusion chromatography curves reflects elution patterns consistent with monomeric binders being the most prominent purified species (Supplementary Fig. 5). Circular dichroism thermal melts (Fig. 2b) obtained for each binder indicate that the binder folds remain intact at high temperatures, even those with significant edge β-strand content. ALK-3bp was stable and monomeric up to 95 °C during a circular dichroism thermal melt experiment despite having four exposed edge β-strands. The binder with the second most β-strand content, ALK-2bp, was also thermostable with a measured TM of 81.2 °C. Binders with majority ɑ-helical support for their β-strand interfaces (KITbp, α-CTXbp, and PDGFRɑbp-AF2model) did not seem to be intrinsically more thermostable with measured TMs of 63.2 °C, 95 °C, and 95 °C, respectively. The binders with ɑ/β secondary structure content (2-3 β-strand sheets with 2 buttressing ɑ-helices; FCRL5bp, NRP1bp, and PDGFRɑbp-7LBF) had TMs of >95 °C, 77.2 °C, and 71.1 °C, respectively. The excellent solution behavior of the designs despite having edge β-strands clearly available for intermolecular interactions suggests that the same idiosyncratic features that enable high affinity and specificity (see below) target binding disfavor self-self interactions^9^.

### β-strand pairing binder interfaces are target specific

**Fig. 3:**
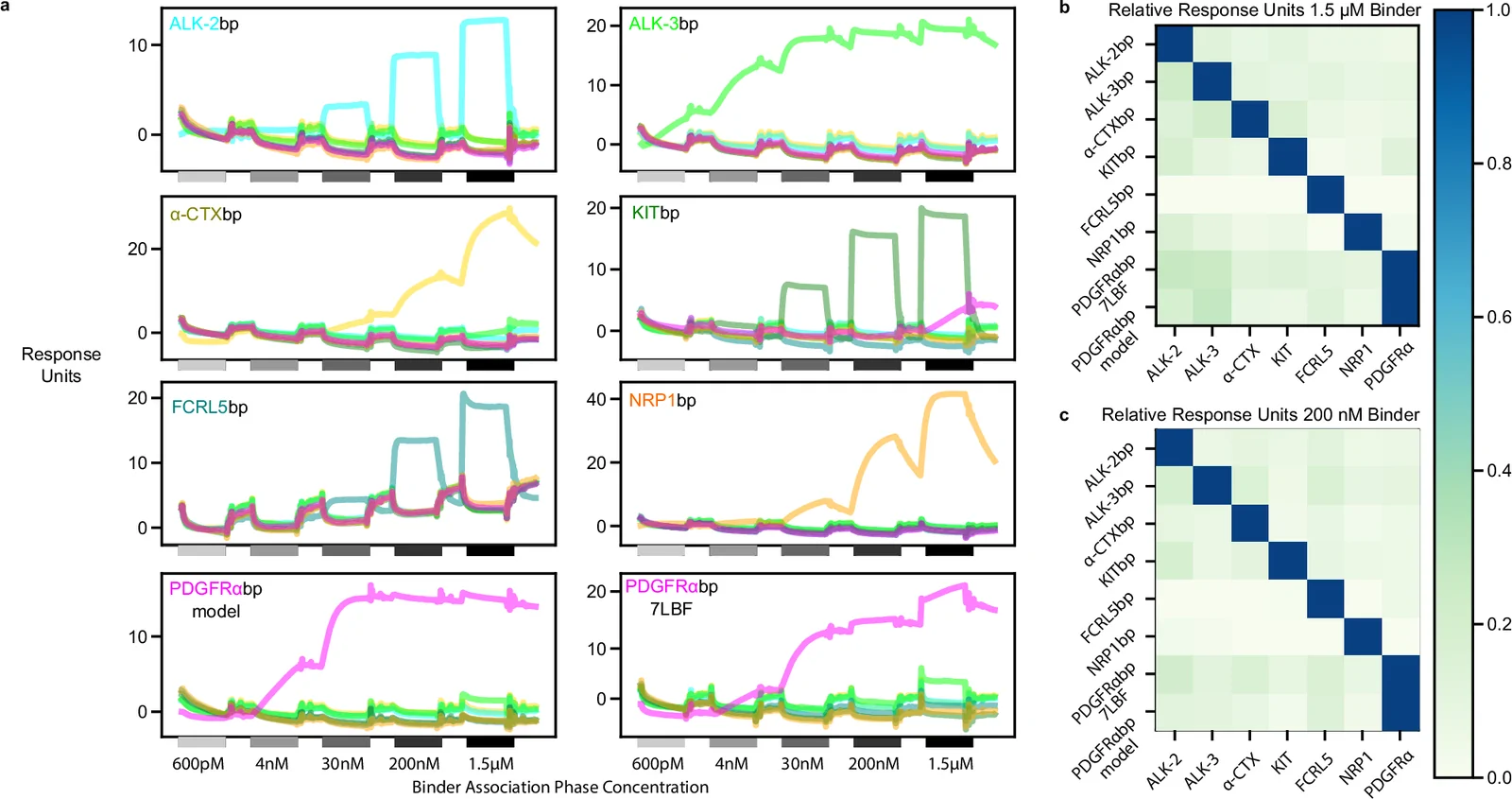
Designed binders are highly specific for their targets. **a** Designed binder SPR response traces against all targets. For each binding protein, immobilized cognate target protein yielded the strongest SPR binding response at various binder concentrations (7-fold increasing binder concentrations ranging from 600pM to 1.5 μM, from left to right in each trace). Average response units for the (**b**) 1.5 μM and (**c**) 200 nM binder concentrations. Grid colors indicate response units relative to the maximum response for each binder. Source data are provided as a Source Data file.

We next investigated whether the identified binders were specific for their designed target and did not form off target high affinity β-strand pairing interactions. To test this, we performed an all-by-all SPR experiment where each binder was tested for binding affinity against each of the receptor targets in the test set. At 1.5 μM and 200 nM concentrations, all of the binders showed strong SPR response for their intended target receptor compared to off-target receptors (Fig. 3a). There was no evidence for off-target binding of the ALK-2, ALK-3, KIT, and PDGFRɑ binding proteins to related family members (e.g., ALK-2bp did not bind strongly to ALK-3). This high specificity may arise because the binders for each structurally similar target pair (i.e., ALK-2 and ALK-3, KIT and PDGFRɑ, FCRL5 and NRP1), were designed with different β-strand pairing hydrogen bonding arrays, and each binder makes additional non-strand-pairing contacts with the intended target. With the exception of ALK-3bp which forms exclusively β-strand contacts with ALK-3, all designed binders form additional ɑ-helical contacts that are complementary to neighboring target surface topologies. Overall, the interface side chain contacts resemble natural protein interfaces, with binder side chains making electrostatic and shape complementarity interactions with target side chains. The polar interactions made by strand-pairing lead to lower overall hydrophobicity, as measured by Spatial Aggregation Propensity (Supplementary Fig. 6b).

### KITbp:KIT complex structure confirms design model accuracy

**Fig. 4:**
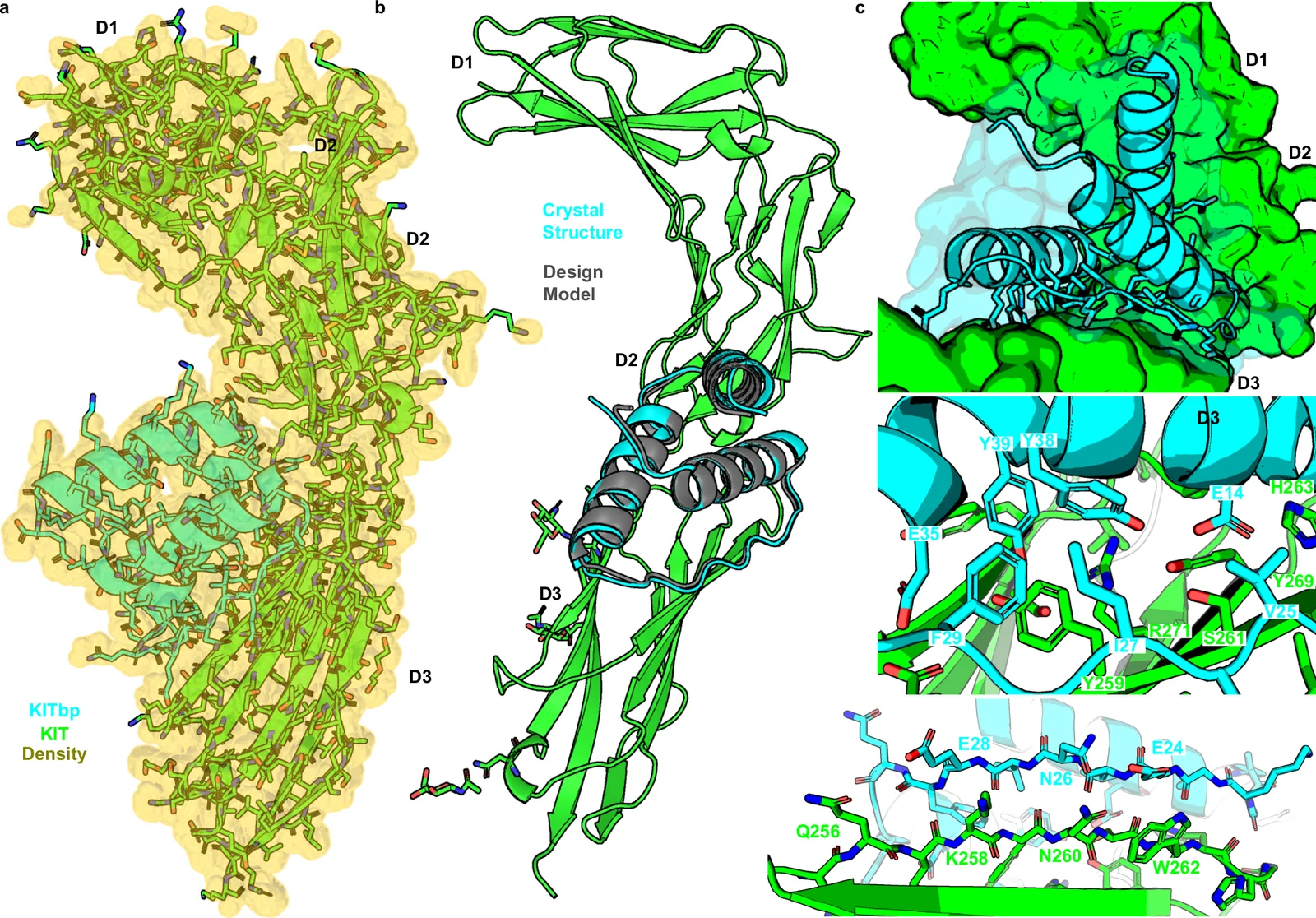
Structural analysis of the KITbp-KIT complex. **a** Crystal structure (PDB ID: 9H71) of KITbp (cyan) in complex with KIT (green) and 2Fo-Fc electron density contoured at +1.0 RMSD (gold). **b** The KITbp crystal structure superimposes on the binder design model (grey) with subangstrom backbone atom RMSD, and 2.0 Å all-atom RMSD. **c** Close-up views of the binder interface reveal high shape complementarity of the binder-target complex (top). The binder interface consists of hydrophobic and polar interactions between binder core-boundary residues and KIT receptor domain 2 core-boundary residues (middle). The binder forms an extensive hydrogen-bond β-pairing interaction with the targeted domain 2 edge strand (bottom). There are multiple side-chain interactions between binder and KIT tyrosine residues (Y38 and Y39 in the binder; Y259 and Y269 in KIT) in the core-boundary interaction, and between binder glutamate residues (E14, E28, E24, E35) and KIT hydrogen bond donors (Q256, K258, N260, W262, H263, R271). The binder also makes electrostatic interactions with both the opposite domain 2 edge strand and KIT domain 3. Source data are provided as a Source Data file.

To assess the accuracy of our design method, we solved the structure of KITbp in complex with domains 1–3 of KIT at 2.8 Å resolution (Fig. 4a, Supplementary Table 2). The crystal structure has near exact agreement with the computational design model with 1.9 Å all-atom RMSD between the design model and the structure. Over the designed binder alone, the RMSD over all atoms between the crystal and design is 2.0 Å, and over the backbone, 0.98 Å RMSD (Fig. 4b). The design model and crystal structure align with atomic level accuracy over nearly all interfacial side chain residue atoms. The KITbp binding site on KIT overlaps with that of the KIT native ligand stem cell factor (SCF), as all the binder designs targeted this site. Consistent with this, saturating concentrations of SCF (60μM) reduced KIT library binding sort counts by 99% in a yeast surface display binding assay (Supplementary Table 2).

The 29 residue binder solvent exposed surface area (SASA) of the binder interface (1164 Å^2^) consists of 8 strand-pairing binder residues (from Gly21 to Asp28, 291 Å^2^) and 21 additional interface residues (873 Å^2^). By heavy atom SASA, the polar atoms comprise 65% of the strand-pairing interface, a somewhat higher proportion compared to the rest of the interface surface, which was only 45% polar. There are 21 sidechain-sidechain and backbone-backbone hydrogen bonds between KITbp and KIT (calculated by HBPLUS^64^), many more than in previous designed binders (Supplementary Fig. 6) and in most native protein-protein interfaces with similar interface sizes^65^ (Fig. 4c). The eight strand pairing binder residues participate in nearly half (9) out of the total (21) hydrogen-bonds in the 29 residue binder interface. The majority of these hydrogen bonds were predicted accurately in our design model (16 out of 21). At the center of the interface, a network of four binder and KIT tyrosine residues (Y38, Y39, Y259, Y269) form a highly complementary interface between the binder interfacial helix and core-boundary of the KIT domain 2 Ig fold. Lining the perimeter of the interface are numerous polar interactions, including the designed β-pairing interface as well as several polar side-chain interactions.

KITbp contains an unpaired β-strand that pairs with the target, with three buttressing ɑ-helices that tether the β-strand in place. Foldseek^66^ alignment of KITbp against the PDB did not identify significant matches to known protein structures. We were unable to find examples of lone β-strands in natural proteins, except those found in protein-protein complexes where a loop in one partner forms a β-strand conformation that extends the β-sheet of the other partner^67^. A similar induction of β strand formation upon binding may occur with KITbp; Alphafold 2 prediction of the monomeric KITbp without KIT target places the β-strand pairing residues in a coil conformation that better shields the binder’s hydrophobic core residues and hydrogen bonding atoms of the β-strand interface (Supplementary Fig. 8); such KIT-dependent conformational switching may contribute to the observed binding specificity.

### Biological functionality of designed binders

**Fig. 5:**
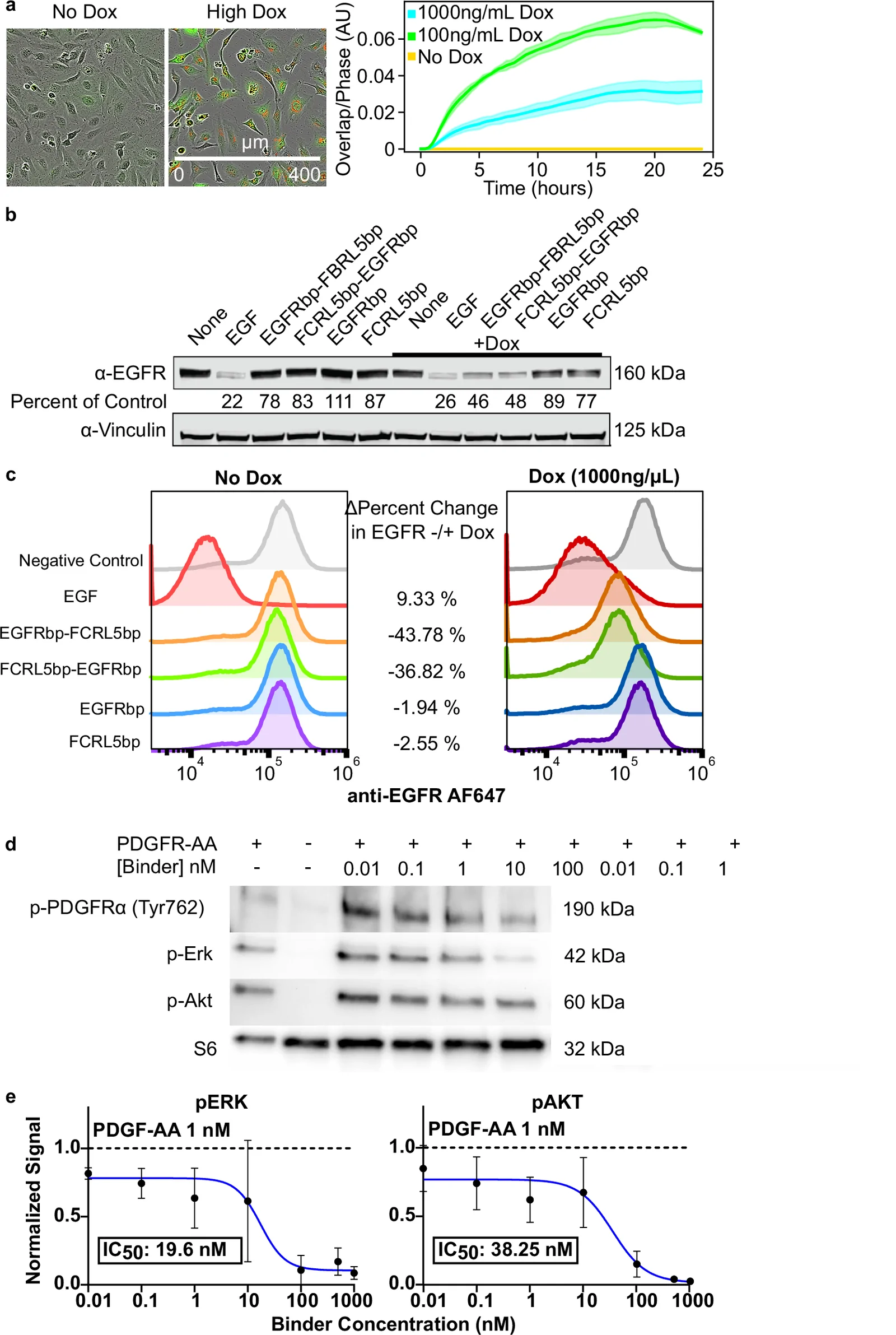
Functional activity of FCRL5 and PDGFRα binding proteins. **a** HeLa cells engineered to express FCRL5 (HeLa-FCRL5) receptor in a Dox-inducible manner were treated with 50 nM neutravidin-labeled pHrodo DeepRed complexed FCRL5bp, and live cell imaging (top panels) was used to measure overlap of GFP (FCRL5-expressing cells) and pHrodo Red fluorescence (internalized FCRL5bp). 1000 ng/mL doxycycline results in strong GFP expression and intracellular pH activated pHrodo Red fluorescence. Internalization (measured arbitrary units calculated by overlap of red and green fluorescence divided by phase area) reached steady state in 18 hours (images are from this timepoint); values are presented as mean ± SD for *n* = 3 replicate wells. **b**, **c** EGFR is degraded HeLa-FCRL5 cells by de novo binders. Measuring EGFR degradation by immunostaining and Western blot (**b**) or flow cytometry (**c**); *n* = 3 independent biological replicates for western blots and flow cytometry. FCRL5bp fusion to EGFRbp resulted in comparable degradation to the native degradation mechanism (EGF signaling). Gating strategies for flow cytometry are provided in Supplementary Fig. 9. **d**, **e** Western blot analysis of PDGFRα inhibition in Chinese Hamster Ovary cells engineered to overexpress PDGFRα (repeated four times with similar results). Levels of phosphorylated PDGFRα, Erk, and Akt were measured by immunoblots with fluorescent antibodies. Signals were normalized by the fluorescent signal of an antibody against the constitutively expressed housekeeping proteins S6 or actin. Data are presented as mean ± SD of *n* = 3 independent biological replicates. Source data are provided as a Source Data file.

We next sought to assess the biological functionality of the designs. FCRL5 is internalized and transits through the endocytic degradation pathway upon binding to antibodies^63^, and we investigated whether FCRL5bp could be similarly internalized. We incubated cells expressing FCRL5 with FCRL5bp tagged with pHrodo DeepRed, a pH sensitive (~5 pK_a_) dye that emits 655 nm fluorescence at late endocytic vesicle pH, and observed binder internalization that correlated with FCRL5 expression levels, reaching a steady state in this expression system (Fig. 5a). We hypothesized that FCRL5bp could thus be useful as an Endotag^52^ for targeted protein degradation, as recently demonstrated for other designed proteins. We fused FCRL5bp to EGFRbp (EGFRn from Cao et al.) in both terminal orientations and compared the ability of this molecule to degrade EGFR by endocytic lysosomal trafficking induced by FCRL5 binding. After treating cells with 50 nM of either EGF, the binder fusions, or each binder alone, EGFR was robustly degraded in a manner dependent on doxycycline induced expression of FCRL5.

PDGFRɑbp-7LBF was designed to sterically occlude the binding site of the native ligand PDGF-AA, and indeed PDGFRɑbp-7LBF blocked activation of signaling by PDGF-AA through the Akt and Erk pathways with nanomolar inhibition of ERK phosphorylation (IC_50_ = 19.6 nM) and AKT phosphorylation (IC_50_ = 38.25 nM) in a CHO cell line engineered to stably express PDGFRɑ (Fig. 5d-e). ALK-2bp, ALK-3bp, and NRP1bp could not be tested for functional agonism in a straightforward manner, as the native ligands for ALK-2 and ALK-3 promiscuously activate signaling with other BMP receptors and NRP1 acts as a cofactor in signaling for various RTKs. In a companion study, α-CTXbp was found to potently neutralize α-CTX, protecting mice from a lethal neurotoxin challenge^62^.

## Discussion

We show through in silico modeling and high throughput binding assays that β-strand interface conditioned RFdiffusion outperforms alpha-helical focused methods for the design of binders against seven edge β-strand containing targets. The binders are thermostable and target-specific, and the crystal structure of the KITbp:KIT complex shows the method has high structural accuracy. The higher success rate of binder design against polar targets containing β-sheets using the β-pairing targeted approach than with primarily ɑ-helical designs likely reflects the difficulty for the latter of complementing the many polar NH and CO groups on edge β-strands with designed sidechain-backbone hydrogen bonds–using geometrically matched β-strands this polar interface can be achieved in less convoluted fashion.

As noted by the Richardsons^9^, edge β strands of native proteins often have irregular geometries that they speculated functioned to suppress off-target β strand mediated interactions with themselves and with other proteins. The very high binding specificity and the lack of significant aggregation of our designs despite the availability of edge β-strands for intermolecular interactions supports the conjecture that non-canonical β strand geometries suppress off target pairing, and considerably extends the pre-deep learning observations of Sahtoe et al. in demonstrating that the irregular edge β-strands of native proteins can be systematically targeted provided that the geometry of the binder strand is sculpted to match the target.

Our β-strand targeted RFdiffusion approach should enable facile binder design against many previously challenging protein surfaces, including the many Ig domains and other β-sheet containing domains frequent in the extracellular domains of cell surface receptors. As many of these are current therapeutic targets, the method could contribute to new therapeutic interventions. While we acknowledge that the binders presented here may not be immediately feasible as therapeutic molecules, we expect that further optimization with these binders as starting points may improve their affinity, stability, pharmacokinetics, and other desired characteristics (e.g., mouse/human cross-reactivity, oligomerization, or protease stability) for therapeutic applications.

## Methods

### In silico comparison of RF diffusion-generated scaffolds

Binder scaffolds were generated with RFdiffusion conditioned with hotspots alone or with β-strand interface conditioning against the target edge β strand sites indicated throughout this work (Fig. 2, Supplementary Fig. 2). Interface conditioning tensors for this were pre-made with the script (see Data Availability) to ask RFdiffusion to create binder interface strands of equal length to the target edge β-strand. Scaffolds were compared by calculating the TM-score^60^ of each binder scaffold against all other binder scaffolds against all targets in the set. This data was transformed into a 2D-array with t-SNE transformation function in scikit-learn (version 0.24.2) to produce the graph in Fig. 2. After generation of ten sequences per scaffold with ProteinMPNN, sequence qualities were assessed with AlphaFold 2 initial guess^6^ and Rosetta FastRelax^1^.

### Production scale computational protein design workflow

Target structures used as inputs for binder design were obtained from the Protein Data Bank for KIT (PDB:2E9W)^68^, NRP1 (PDB:2QQI)^69^, and PDGFRɑ (PDB:7LBF)^70^. Publicly available AlphaFold 2 models were used for the design of binders against ALK-2 (Uniprot:Q04771, Accessed October 2022), ALK-3 (Uniprot:P36894, Accessed October 2023), FCRL5 (Uniprot:Q96RD9, Accessed February 2023), and PDGFRɑ (Uniprot:P16234 Accessed April 2023). Target surfaces were assessed for surface hydrophobics and edge strands. Five to ten thousand binder backbone scaffolds complementing these sites were generated by standard RFDiffusion design with hotspot conditioning as well as β-strand interface conditioning. To condition RFdiffusion towards generating β-strand interfaces, the script introduced in this work (Data Availability) was used to create conditioning tensors that guide RFdiffusion toward generating interface strands of equal length to the target edge β-strand. ProteinMPNN sequences were generated for the output backbones and subject to in silico screening based on AlphaFold 2 initial guess (pAE interaction and binder pLDDT)^6^, AlphaFold 2 monomer pLDDT^54^, Rosetta ΔΔG^1^, radius of gyration (ROG), and spatial aggregation propensity (SAP)^71^. The cutoff values for these metrics varied somewhat for each target protein, but obtained minimum values of pAE interaction <10, pLDDT >85, ΔΔG < −30, a variable ROG filter adjusted for binder size length/4.5 = ROG, and SAP < 45 (more details can be found in Supplementary Table 1). In some cases, in silico optimization was carried out by iterative partial RFdiffusion and ProteinMPNN cycling^72^. These filters were used for in silico selection of top binder designs from hundreds of thousands of sequences, resulting in libraries ranging in size from 96 to 5000 that were screened experimentally. In silico and experimental library sizes depended on the in silico filter success rate, and the perceived difficulty of designing a binder interface based on target surface concavity, target surface hydrophobicity measured by SAP, and known previous experimental challenges (as for ALK-2, ALK-3, KIT, unpublished data). To ensure our binder design libraries sampled diverse sequences and structures, we clustered designs by sequence similarity or RMSD and selected a subset of designs such that each cluster was equally represented in the final library for experimental characterization.

### DNA library preparation

For ALK-2, ALK-3, KIT, FCRL5, and PDGFRɑ, 10^4^ scale DNA libraries were generated by using DNAworks2.0 reverse translating designed amino acid sequences that optimally reflected the Saccharomyces cerevisiae codon frequency table. Additional 5‘ and 3‘ adapters were included to enable PCR amplification of libraries by single sets of primers. All libraries were amplified using Kapa HiFi polymerase (Kapa Biosystems) with a qPCR machine (Bio-Rad, CFX96). In detail, the libraries were first amplified in a 25 μl reaction, and the PCR reaction was terminated when the reaction reached half maximum yield to avoid overamplification. The PCR product was loaded onto a DNA agarose gel. The band with the expected size was cut out, and DNA fragments were extracted using QIAquick kits (Qiagen). Then, the DNA product was re-amplified as before to generate enough DNA for yeast transformation. The final PCR product was cleaned up with a QIAquick Clean up kit (Qiagen). For the yeast transformation step, 2–3 µg of linearized modified pETcon vector (pETcon3) and 6 µg of insert were transformed into the EBY100 yeast strain using the lithium-acetate/PEG protocol of ref. ^73^.

DNA libraries for deep sequencing were prepared using the same PCR protocol, except the first step started from yeast plasmid prepared from 5 × 10^7^ to 1 × 10^8^ cells by Zymoprep (Zymo Research). Illumina adapters and 6-bp pool-specific barcodes were added in the second qPCR step. Gel extraction was used to obtain the final DNA product for sequencing. All the different sorting pools were sequenced using Illumina NextSeq sequencing.

### Target protein preparation

Biotinylated target protein was commercially available for KIT (Acro Biosystems, CD7H5255), FCRL5 (Acro Biosystems, FC5-H82E3), NRP1 (Acro Biosystems, NR1-H82E3), α-CTX (Latoxan, L8114), and PDGFRɑ (Sino Biological, 10556-H27H-B). For ALK-2, ALK-3 biotinylated receptor was expressed as avi-tagged ectodomain constructs in *E. coli*, purified, and biotinylated as described by Tao Huang and Andrew P. Hinck^74^.

### Yeast display

Saccharomyces cerevisiae EBY100 strain cultures were grown in C-Trp-Ura medium supplemented with 2% (w/v) glucose. For induction of expression, yeast cells were centrifuged at 4500 × *g* for 5 min and resuspended in SGCAA medium supplemented with 0.2% (w/v) glucose at the cell density of 1 × 10^7^ cells per ml and induced at 30 °C for 16–22 h. Cells were washed with PBSF (PBS with 1% (w/v) BSA) and labeled with biotinylated targets using two labelling methods: with-avidity and without-avidity labeling. For the with-avidity method, the cells were incubated with biotinylated target, together with anti-c-Myc fluorescein isothiocyanate (FITC, Miltenyi Biotech) and streptavidin–phycoerythrin (SAPE, ThermoFisher). The concentration of SAPE in the with-avidity method was used at one-quarter of the concentration of the biotinylated targets. For the without-avidity method, the cells were first incubated with biotinylated targets, washed and secondarily labeled with SAPE and FITC. All the original libraries of de novo designs were sorted using the with-avidity method for the first few rounds of screening to exclude weak binder candidates, followed by several without-avidity sorts with different concentrations of biotinylated targets. Binder affinities were estimated from this titration data using the software provided (Data Availability) to estimate SC_50_, originally introduced by Cao et. al.

### Protein expression and purification

Protein binder designs were ordered as synthetic genes (eBlocks, Integrated DNA Technologies) and cloned via BsaI overhangs into the target cloning vector, LM0627^75^ for Golden Gate assembly. Golden Gate reaction mixtures were transformed into a chemically competent expression strain (BL21(DE3)), and overnight outgrowth cultures were used to seed 500 mL protein expression cultures in auto-induction media (autoclaved TBII media supplemented with Kanamycin, 2 mM MgSO4, 1×5052) and to propagate expression plasmid for isolation (QIAprep Spin Miniprep Kit) and sanger sequencing services performed by GENEWIZ (Azenta Life Sciences). The following day (20-24 hrs later), cells were harvested and lysed by sonication (QSonica Q500 Sonicator), and clarified lysates were purified by immobilized metal affinity chromatography using Ni-NTA agarose resin (Qiagen). Samples were eluted in a Tris elution buffer containing 300 mM imidazole, sterile filtered with 0.22μm Polyvinylidene Fluoride syringe filter prior to size exclusion chromatography. Protein designs were then screened via SEC using an AKTA FPLC outfitted with an autosampler capable of running samples from a 96-well source plate. The protein binders were run on a Superdex75 Increase 5/150 GL column (Cytiva 29148722) at room temperature.

### Circular dichroism

Far-ultraviolet circular dichroism measurements were carried out with a JASCO-1500 instrument equipped with a temperature-controlled multi-cell holder. Wavelength scans were measured from 260 to 190 nm at 25 and 95 °C. Temperature melts monitored the dichroism signal at 217 nm in steps of 2 °C/min with 30 s of equilibration time. Wavelength scans and temperature melts were performed using 0.3 mg/ml protein in PBS buffer (20 mM NaPO4, 150 mM NaCl, pH 7.4) with a 1 mm path-length cuvette. Melting temperatures were determined by fitting the data with a sigmoid curve equation. For designs retained more than half of the mean residue ellipticity values, Tm values are reported as greater than 95 °C. Tm values of the other designs were determined as the inflection point of the fitted function.

### Surface plasmon resonance measurement of binding kinetics

Binding kinetics were analyzed via Surface Plasmon Resonance (SPR) on a Biacore 8 K (Cytiva) operated at 25 °C with a data collection rate of 10 Hz. Receptor binding was measured by capturing biotinylated receptor ectodomains on a streptavidin-coated CAPture chip using the Biotin CAPture Kit (Cytiva #28920234). Biotinylated receptors (0.125 µg/mL) were injected for 100 s at a flow rate of 10 µL/min in HBS-EP⁺ buffer (0.01 M HEPES pH 7.4, 0.15 M NaCl, 3 mM EDTA, 0.005% v/v Surfactant P20; Cytiva #BR100669) to achieve capture levels of approximately 250 RU. Analytes were prepared by serial 1:7 dilution from 10 µM stocks in HBS-EP⁺ and injected at a flow rate of 30 µL/min to monitor association, followed by dissociation in running buffer (HBS-EP⁺) at the same flow rate. Single-cycle kinetics were performed by injecting increasing analyte concentrations sequentially over the same captured ligand surface (six concentrations per cycle, 100 s association, 60 s dissociation between each cycle, and 750 s final dissociation). Regeneration was achieved by two 60 s pulses of regeneration buffer at 10 µL/min. Blank-injection (buffer-only) subtraction was applied for all analyte injections, double referencing (reference-surface + blank) was performed. Sensorgrams were processed and fit in the Biacore Insight Evaluation Software using a 1:1 Langmuir binding model. An exception was made to use a heterogenous kinetic fit model for α-CTXbp as this was found in ref. ^62^ to better model the partially biphasic binding properties for the complex. Global fit was applied for kₐ, k_d_, R_max_, and tc parameters with default initial values 10e5, 10–e, Y_max_, 10e8 initial values. Drift was fitted locally (and is reported in the kinetic parameters in the Source Data file), and RI was set to 0 as a constant. Fitting drift was capped at a maximum contribution 15%, where the drift contribution over the 750 s fit could not exceed 15% of the Rmax parameter (capping only performed for KITbp and FCRL5bp for optimal fitting values χ^2^ and SE, while other binders were affected). 10 μM data was excluded from the fit due to non-ideal solution behaviour, observable in the overlaid fits in Fig. 2.

### Surface plasmon resonance to measure target specificity

All by all binder-target interactions were measured according to SPR protocol described above. Identical binder titres and receptor loading protocols were used to maintain as much signal consistency as possible, with binder titre values ranging in seven fold dilutions from 10 μM to 600pM to capture nonspecific binding across a large concentration spectrum. Non-specific binding responses were evaluated by averaging response values across the association phase for a given titre, normalizing responses such that all values were positive, and taking each response as a fraction of the maximum observed response (for each binder the maximum response was observed against the target protein for which it was designed).

### Recombinant production of KIT_D1-D3_ for X-ray crystallography

KIT123, i.e., extracellular domains D1–3 of the tyrosine kinase receptor KIT, was recombinantly produced via transient expression of suspension-adapted HEK 293 cells. Cells were grown and maintained in a 1:1 mixture of the Freestyle (Gibco) and the Ex-Cell (Merck) medium. Before the transfection, cells at the density of 1.5×  10^6^ cells.mL^–1^ were centrifuged at 250 × g for 6 min and resuspended in the pre-warmed FreeStyle medium only to reach the density of 3.0 × 10^6^ cells.mL^–1^ and incubated at 37 °C, 130 rpm, 70 % humidity, and 8.0 % CO_2_ for 15 min. The cells were subsequently added 450 µg of the plasmid DNA carrying the target construct per 100 mL of the medium. After 5 min, the cells were added 900 µg of linear polyethylenimine 25 kDa (Polysciences) and 3 µmol of kifunensine per 100 mL of the medium and continued incubation. After additional 5 h, an equal volume of the Ex-Cell medium to the FreeStyle medium was added to the cultures to return back to the density of ~1.5 × 10^6^ cells.mL^–1^. 24 h post-transfection, the cells were added D-glucose and valproic acid to the resulting concentration of 55 mM and 3.5 mM, respectively. 96 hours post-transfection, the cells were harvested by centrifugation at 500 × g and 4 °C for 10 min and the conditioned medium (supernatant), carrying the recombinantly produced protein of interest, was collected, added 10,000 U of Endo Hf (NEB) per 100 mL of the medium, and incubated for 4 h at room temperature to remove heterogenous N-linked glycans and facilitate the subsequent crystallization attempts. After filtering the incubated medium through a 0.22 µm filter, the clarified sample was loaded to a 1 mL Ni-NTA HisTrap HP column (Cytiva) equilibrated with HEPES-buffered saline (HBS; 20 mM HEPES, 150 mM NaCl, pH 7.4). The column was washed with 10 mM imidazole in HBS and the His-tagged protein of interest was eluted using 150 mM imidazole in HBS. The eluted protein fraction was subsequently loaded to a Superdex 75 Increase 10/300 GL column (Cytiva) to simultaneously remove aggregates and remaining impurities and to exchange buffer to HBS with no imidazole. The fractions corresponding to the protein of interest were pooled together, their purity was analyzed by SDS-PAGE (Bio-Rad), and the concentration was determined using the NanoDrop Spectrophotometer (Thermo Fisher Scientific).

### Crystal structure of KIT123 in complex with KITmb

The KIT123–KITbp complex was formed by adding a 3-fold molar excess of the purified KITbp to the recombinantly produced, EndoH-treated KIT123 receptor (domains D1–3 of the ectodomain). The complex was isolated using size-exclusion chromatography equipped with a Superdex 75 Increase 10/300 GL column (Cytiva) equilibrated with HEPES-buffered saline (HBS; 20 mM HEPES, 150 mM NaCl, pH 7.4). Fractions corresponding to the KIT123–KITbp complex were pooled and concentrated by centrifugal ultrafiltration to the concentration of 6.1 mg.ml^–1^. Sparse-matrix crystallization screens were carried out in 96-well 3-drop plates (Molecular Dimensions) using the BCS-Screen (Molecular Dimensions) at 293 K and the sitting-drop method. The vapour-diffusion geometry was used to set up sitting drops consisting of 100 nL of a protein solution and 100 nL of each reservoir solution using a Mosquito nanolitre crystallization robot (SPT Labtech). The protein complex crystallized in the condition G11 (0.2 M sodium/potassium phosphate pH 7.5, 0.1 M HEPES pH 7.5, 22.5 % PEG Smear Medium, 10 % glycerol). Crystals were cryo-protected with mother liquor supplemented with 25% v/v glycerol and subsequently flash-cooled by direct plunging into liquid nitrogen. X-ray diffraction data of protein crystals were collected at the P13 beamline (PETRA III, EMBL Hamburg). Obtained data were processed using XDS^76^ and severe data pathologies, including strong anisotropy and translational noncrystallographic symmetry, were revealed, yielding similar characteristics as reported previously^77^. Based on these findings, the data were elliptically-truncated and corrected using the STARANISO^78,79^ server and accordingly treated during the following steps. Initial phases were determined by maximum-likelihood molecular replacement in Phaser^80^ using the domains D1–3 part of the KIT-SCF structure (PDB ID: 2E9W)^68^ as a search model. Model (re)building was performed in Coot^81^, and coordinate and ADP refinement was performed in PHENIX^82^. Model and map validation tools in Coot, the PHENIX suite, and the PDB_REDO server^83^ were used to validate the quality of crystallographic models. Atomic coordinates and structure factors of the protein-protein complex were deposited in the Protein Data Bank under the PDB code 9H71.

### In silico binder interface characterization

SAP and SASA metrics were calculated using PyRosetta and PyMOL software using the calculate_sap and get_area functions, respectively. ΔSAP and ΔSASA were obtained by calculating each metric for the binder before and after removing target proteins from complex models. To calculate the polar interface surface, the ΔSASA was calculated for each binder atom. The sum of oxygen and nitrogen ΔSASA values yielded the polar interface values, while all carbon residues were considered to be non-polar. Using the DSSP algorithm, binder-target strand pairing interactions were identified by counting β-strand conformation binder and target residues with backbone interactions less than 3.5 Å in distance. Strand pairing orientation (parallel or antiparallel) was determined by calculating the average position of the N-terminal and C-terminal halves of the binder and target strand-pairing residues. If the distance between the binder N-terminal strand contacts and the target N-terminal strand contacts was less than that of the distance to the target C-terminal strand contacts, the strand-pair was determined to be parallel. Otherwise, it was determined to be antiparallel. The software used to determine these metrics is provided in the Data Availability section.

### PDGFRɑ antagonism assay

Heparan-deficient Chinese hamster ovary cells stably overexpressing PDGFRα (CHO-PDGFRα) were grown to 70-90% confluency in CHO growth medium (Kaighn’s Modification of Ham’s F12 (F12K) medium (ATCC# 30-2004) + 10% Fetal Bovine Serum (FBS) (Biowest, #S1620) + 4% Penicillin-Streptomycin (P/S) (Gibco, #15140122) with 10 μg/mL puromycin (Gibco, #A11138-03). The cells were starved for 4 h in serum free F12K media and treated with synthetic and/or native ligand for 15 min at 37 degrees Celsius. Cells were subsequently washed with PBS and lysed with buffer containing 20 mM Tris–HCl (Sigma-Aldrich, 1185-53-1) (pH 7.5), 150 mM NaCl, 15% glycerol (Sigma-Aldrich, G5516), 1% triton (Sigma-Aldrich, 9002-93-1), 3% SDS (Sigma-Aldrich, 151-21-3), 25 mM β-glycerophosphate (Sigma-Aldrich, 50020-100 G), 50 mM NaF (Sigma-Aldrich, 7681-49-4), 10 mM sodium pyrophosphate (Sigma-Aldrich, 13472-36-1), 0.5% orthovanadate (Sigma-Aldrich, 13721-39-6), 1% PMSF (Roche Life Sciences, 329-98-6), 25 U benzonase nuclease (EMD, 70664-10KUN), protease inhibitor cocktail (PierceTM Protease Inhibitor Mini Tablets, Thermo Scientific, A32963), and phosphatase inhibitor cocktail 2 (Sigma-Aldrich, P5726). Lysates were collected, mixed with 4x Laemmli buffer (BioRad, #161-0747), and boiled at 95 Celsius for 10 min before 10 uL were loaded on to 4–10% SDS-PAGE gels and run for 30 min at 250 Volts.

### Western blotting

The PDGFRɑ antagonism assay was analyzed using two different western blot techniques. One repeat of each was analyzed via traditional techniques which is as follows: after separation, proteins were transferred onto a nitrocellulose membrane (12 min, semi-dry transfer) and blocked for one hour in 5% bovine serum albumin. Membranes were probed with the following primary antibodies: phospho-PDGFRα (Tyr762) (Cell Signaling Technology, #24188) 1:1000 dilution, Phospho-Akt (Ser473) (Cell Signaling Technology, #9271) 1:1000 dilution, Phospho-p44/42 MAPK (Erk1/2) (Thr202/Tyr204) (Cell Signaling Technology, #9101) 1:10,000 dilution, and either S6 (Cell Signaling Technology, #2117) at 1:1000 dilution or H3 (Cell Signaling Technology, #9715) at 1:5000 dilution as a loading control. After overnight incubation on a rocker, the membrane was probed with HRP-conjugated secondary antibody, washed 3 times, and imaged with a Bio-Rad ChemiDoc Imager. The blot image was quantified using ImageJ peak band intensity. In addition, samples were analyzed using a Biotechne Jess Automated Western Blot Machine (Biotechne, #004-650). Samples were diluted 1:3 before being prepared as per the kit instructions for 12-230 kDA separation modules (Biotechne, #SM-W001). Samples were probed using the same primary antibodies as above via a RePlex assay (Biotechne, #RP-001) that was run using the default settings for anti-rabbit chemiluminescence (Biotechne, #DM-001). Signal was quantified using area under the curve for each protein of interest. All data points from each assay were normalized to a housekeeping gene in the same lane and then normalized to the 1 nM PDGF-AA condition within each experiment.

### Vectors and constructs/lentiviral generation and infection

Lentiviral particles of the pHAGE-PDGFRɑ plasmid (Addgene, 116769)^84^ were generated by transfecting 60-80% confluent HEK293FT cells, maintained in 10 mL HEK growth medium (Gibco™ DMEM, high glucose (Gibco, 11965092) + 10% FBS + 1% P/S) on a 100 mm TC-treated culture dish, with 20 µg pHAGE-PDGFRɑ, 15 µg psPAX2 (Addgene, 12260), and 5 µg pMD2.g (Addgene, 12259) combined with 1.8 mL Opti-MEM medium (Gibco, 31985070) and 60 µg linear polyethylenimine. Transfected HEK293FT cells were replenished with fresh HEK growth medium 24 h after the transfection. The supernatant containing lentivirus was collected 48 and 72 h post-transfection. The collected supernatant was filtered through a 0.45 µM PES syringe filter. CHO-PDGFRα cells were generated by infecting 60–80% confluent heparan-deficient Chinese Hamster Ovary cells (CHO) cells (pgsD-677 cells) (# CRL-2244) with the filtered lentivirus-containing supernatant. Infected CHO cells were supplemented with fresh CHO growth medium 24 h after the lentiviral infection. 48 h after the infection, CHO cells were selected in the CHO selection medium (CHO growth medium containing 10 µg/mL puromycin). CHO selection medium was replaced every 48 h for 7 days. CHO-PDGFRα cells post-selection were maintained in the selection medium.

### FCRL5 cell line preparation

Doxycycline-inducible expression of Flag-FCRL5-P2A-T2A-EGFP was generated by first seeding 1 ×106 WT HeLa cells in 10 cm dish (Genesee 25-202) in Dulbecco’s Modified Eagle Medium (DMEM, Gibco 11995073) supplemented with 10% heat-inactivated fetal bovine serum (HI FBS, Gibco A5256801) and 1% penicillin-streptomycin (PS, Gibco 15140122). The next day, cells were transfected using TransIT-LT1 Transfection reagent (Mirus Bio MIR2300) according to manufacturer protocol with 2:1 donor:sleeping beauty transposase plasmids (Addgene Plasmid #34879)^85^. After 72 h, cells were covered with selection media comprised of DMEM + 10 % HI FBS + 1% PS and 2 μg/mL puromycin (Invivogen 58-58-2). Selection media was replaced every 48 h until control WT cells were completely dead. Cells were maintained in selection media.

### FCRL5bp internalization assay

Flag-FCRL5 HeLa cells were counted and seeded at 50,000 per well in a 24-well plate (Genesee 25-107) in DMEM + 10% HI FBS + 1% PS. The next day, cells were treated with either 0, 100, or 1,000 ng/mL doxycycline (Fisher Scientific BP26531), and incubated for 48 h at 37 °C 5% CO_2_. Cells were then lifted, counted using TrypanBlue, and seeded at 12,500 cells per well in a 96-well plate (Corning 3595) in phenol red-free DMEM (Gibco 31053028) supplemented with 10 % HI FBS and 0, 100, or 1000 ng/mL. Cells were incubated at 37 °C 5% CO_2_ for 8 h. Then, AviTagged-FCRL5bp was diluted in phenol red-free DMEM + 10% HI FBS and respective doxycycline concentrations. Then, FCRL5bp was complexed 1:1 with TFP ester-pHrodoDeepRed (Invitrogen P35358) labeled NeutrAvidin (Thermo Scientific 31000) for 15 min at 37 °C covered from light. Media was then replaced with respective treatments, and cells were monitored with a live-cell imaging incubator (Sartorius Incucyte S3) and internalization was quantified as the overlap of red and green fluorescent area divided by the total phase area in a well.

### FCRL5bp-EGFRn bifunctional degradation assay

Flag-FCRL5 HeLa cells were counted and seeded at 300,000 per well in a 6-well plate (Genesee 25-105) in DMEM + 10% HI FBS + 1% PS and incubated overnight at 37 °C, 5% CO_2_. Then, cells were treated with either 0 or 1000 ng/mL doxycycline (Fisher Scientific BP26531) and incubated for 48 h at 37 °C, 5% CO_2_. Media was then replaced with treatments of 100 ng/mL EGF (Gibco AF10015) or 50 nM of respective FCRL5bp, EGFRn, or bifunctional treatment were prepared in media with and without 1,000 ng/mL doxycycline. Cells were incubated with treatments for 48 h at 37 °C, 5% CO_2_, then cells were lifted in 600 uL total volume. One-sixth of the total volume of cells was reserved for live-cell immunostaining and flow cytometry. Cells were washed three times by centrifuging for 5 min at 4 °C, 500 g, discarding supernatants, and resuspending in ice-cold PBS + 1% BSA (Sigma-Aldrich A3608). After the final wash, cells were resuspended in PBS + 1% BSA + 4 ug/mL anti-EGFR (Thermo Scientific MA513319) and incubated on ice for 30 min before repeating washing three times in PBS + 1% BSA. Cells were then resuspended in PBS + 1% BSA + 4 ug/mL Alexa Fluor 647 AffiniPure Goat Anti- Mouse IgG (H + L) (Jackson ImmunoResearch Laboratories 115-605-003) and incubated on ice for 30 min covered from light. Cells were then washed three times and quantified on BD Accuri C6 Plus flow cytometer, and data were processed using FlowJo v10 Software. The remaining cells were washed three times in ice-cold PBS before resuspending in RIPA lysis buffer (Thermo Scientific 89900) + protease inhibitor cocktail tablet (Thermo Scientific A32955) and lysing cells on ice for 15 min. Lysates were then centrifuged at 4 °C, 18,000 g for 15 min. The protein concentrations were quantified with a bicinchoninic acid assay kit (Thermo Scientific 23227) according to manufacturer’s protocols. Samples were prepared at equal protein with NuPage 4x lithium dodecyl sulfate buffer (Invitrogen NP0007) + 0.1 M DL- dithiothreitol (Thermo Scientific R0861), and boiled at 95 °C for 10 min before spinning down and loading on a 4–12% Bis-Tris protein gel (Bio-Rad 3450124). The gel was loaded and ran at 200 V for 1 h in 1x XT MES running buffer (Bio-Rad 1610789). The gel was then released from its cassette and transferred to a nitrocellulose membrane using a Trans-Blot Turbo Transfer Kit (Bio-Rad1704271) for 15 min at 25 A and 2.5 V. The membrane was then trimmed and blocked for 1 h at RT in Intercept (PBS) blocking buffer (LI-COR 927-70001). Then, the membrane was covered with blocking buffer + D38B1 anti-EGFR antibody (Cell Signaling Technology 4267) and incubated at 4 °C overnight on a rocker. The membrane was then washed three times for 5 min in PBS + 0.1 % Tween-20 (Thermo Scientific J20605.AP), and incubated for 1 h rocking at RT in goat anti-rabbit IgG (H + L) antibody IRDye 800CW (LI-COR 926-32211) in blocking buffer. The membrane washing procedure was repeated, then rinsed three times in PBS before imaging on an Odyssey CLx Imaging System. The membrane was then incubated overnight at 4 °C in blocking buffer + 7F9 anti-Vinculin antibody (Santa Cruz Biotech sc-73614). The washing procedure was repeated and the membrane was incubated for 1 h at RT in goat anti-mouse IgG (H + L) antibody IRDye 680RD (LI-COR 926-68070) in blocking buffer. The washing, rinsing, and imaging procedure was repeated. EGFR staining was quantified relative to vinculin staining using Image Studio software, and reported as a percentage relative to respective no doxycycline or +doxycycline control.

### Reporting summary

Further information on research design is available in the Nature Portfolio Reporting Summary linked to this article.

## Supplementary information

Supplementary Information

Reporting Summary

Transparent Peer Review file

## Source data

Source Data

## Data Availability

Source data are provided with this paper, including all experimental data presented in this manuscript. Namely, In silico datasets, SPR traces, parameters for kinetic fits, and functional binder experimental data, and the data presented in the supplemental information file are included. Crystallographic data for the KITbp–KIT complex have been deposited in the Protein Data Bank under accession code PDB 9H71. Data corresponding to the α-CTX binder (α-CTXbp) were previously reported by Vázquez Torres in ref. ^62^. The Crystallographic data used here are available under PDB accession code 9BK6. No restrictions apply to data access. Larger files (PDB files, sequences, and analysis scripts for our in silico experiments, FACS data, design methods scripts and instructions, PDB files that were experimentally screened, and previously unpublished sequences that had been tested) can be found at: https://files.ipd.uw.edu/pub/strand_pairing_binders_2025/in_silico_benchmark_outputs.zip, https://files.ipd.uw.edu/pub/strand_pairing_binders_2025/anaysis_scripts.zip, https://files.ipd.uw.edu/pub/strand_pairing_binders_2025/facs_data.zip, https://files.ipd.uw.edu/pub/strand_pairing_binders_2025/target_pdbs.zip, https://files.ipd.uw.edu/pub/strand_pairing_binders_2025/interface_tensors.zip, https://files.ipd.uw.edu/pub/strand_pairing_binders_2025/experimentally_tested_design_pdbs.zip, https://files.ipd.uw.edu/pub/strand_pairing_binders_2025/alk3_kit_failed_libraries.zip. Source data are provided with this paper.

## Code availability

Code for running RFdiffusion has been released on GitHub, free for academic, personal and commercial use at https://github.com/RosettaCommons/RFdiffusion. An interface conditioning script that can be used to replicate the approach here is provided at https://files.ipd.uw.edu/pub/strand_pairing_binders_2025/interface_tensors.zip, along with scripts used to calculate SC_50_ values from yeast surface display titrations (as originally reported by Cao et. al.), scripts for calculating TMscores and for deriving and plotting t-SNE transformations of this data. The code is distributed under the MIT License and can be freely reused and modified. A script to run AF2 with an initial guess as was used in this work is provided at this Zenodo link: https://doi.org/10.5281/zenodo.7730843 ref. ^6^.

## References

[1] Cao, L. et al. Design of protein-binding proteins from the target structure alone. *Nature* **605**, 551–560 (2022). 10.1038/s41586-022-04654-9 PMC9117152 35332283

[2] Watson, J. L. et al. De novo design of protein structure and function with RFdiffusion. *Nature* **620**, 1089–1100 (2023). 10.1038/s41586-023-06415-8 PMC10468394 37433327

[3] Gainza, P. et al. De novo design of protein interactions with learned surface fingerprints. *Nature* **617**, 176–184 (2023). 10.1038/s41586-023-05993-x PMC10131520 37100904

[4] Vázquez Torres, S. et al. De novo design of high-affinity binders of bioactive helical peptides. *Nature* **626**, 435–442 (2024). 10.1038/s41586-023-06953-1 PMC10849960 38109936

[5] Cao, L. et al. De novo design of picomolar SARS-CoV-2 miniprotein inhibitors. *Science* **370**, 426–431 (2020). 10.1126/science.abd9909 PMC7857403 32907861

[6] Bennett, N. R. et al. Improving de novo protein binder design with deep learning. *Nat. Commun.* **14**, 2625 (2023). 10.1038/s41467-023-38328-5 PMC10163288 37149653

[7] Stranges, P. B. & Kuhlman, B. A comparison of successful and failed protein interface designs highlights the challenges of designing buried hydrogen bonds: Successful Interface Designs Avoid Polar Interactions. *Protein Sci.* **22**, 74–82 (2013). 10.1002/pro.2187 PMC3575862 23139141

[8] Watkins, A. M. & Arora, P. S. Anatomy of β-strands at protein-protein interfaces. *ACS Chem. Biol.* **9**, 1747–1754 (2014). 10.1021/cb500241y PMC4136675 24870802

[9] Richardson, J. S. & Richardson, D. C. Natural beta-sheet proteins use negative design to avoid edge-to-edge aggregation. *Proc. Natl. Acad. Sci. USA* **99**, 2754–2759 (2002). 10.1073/pnas.052706099 PMC122420 11880627

[10] Sahtoe, D. D. et al. Transferrin receptor targeting by de novo sheet extension. *Proc. Natl. Acad. Sci. USA* **118**, e2021569118 (2021). 10.1073/pnas.2021569118 PMC8092486 33879614

[11] Fujiwara, K., Ebisawa, S., Watanabe, Y., Toda, H. & Ikeguchi, M. Local sequence of protein β-strands influences twist and bend angles: Side Chain Influences β-Strand Twisting. *Proteins* **82**, 1484–1493 (2014). 10.1002/prot.24518 24464770

[12] Fujiwara, K., Ebisawa, S., Watanabe, Y., Fujiwara, H. & Ikeguchi, M. The origin of β-strand bending in globular proteins. *BMC Struct. Biol.* **15**, 21 (2015). 10.1186/s12900-015-0048-y PMC4618951 26492857

[13] Richardson, J. S., Getzoff, E. D. & Richardson, D. C. The beta bulge: a common small unit of nonrepetitive protein structure. *Proc. Natl. Acad. Sci. USA* **75**, 2574–2578 (1978). 10.1073/pnas.75.6.2574 PMC392604 275827

[14] Fox, N. K., Brenner, S. E. & Chandonia, J.-M. SCOPe: structural classification of proteins-extended, integrating SCOP and ASTRAL data and classification of new structures. *Nucleic Acids Res.* **42**, D304–D309 (2014). 10.1093/nar/gkt1240 PMC3965108 24304899

[15] Chandonia, J.-M. et al. SCOPe: improvements to the structural classification of proteins - extended database to facilitate variant interpretation and machine learning. *Nucleic Acids Res.* **50**, D553–D559 (2022). 10.1093/nar/gkab1054 PMC8728185 34850923

[16] Bragdon, B. et al. Bone morphogenetic proteins: a critical review. *Cell. Signal.* **23**, 609–620 (2011). 10.1016/j.cellsig.2010.10.003 20959140

[17] Sorkin, M. et al. Hair follicle specific ACVR1/ALK2 critically affects skin morphogenesis and attenuates wound healing. *Wound Repair Regen.* **25**, 521–525 (2017). 10.1111/wrr.12549 PMC5568946 28513105

[18] Liu, M., Goldman, G., MacDougall, M. & Chen, S. BMP signaling pathway in dentin development and diseases. *Cells* **11**, 2216 (2022). 10.3390/cells11142216 PMC9317121 35883659

[19] Sugimoto, H. et al. Activin-like kinase 3 is important for kidney regeneration and reversal of fibrosis. *Nat. Med.* **18**, 396–404 (2012). 10.1038/nm.2629 PMC3998727 22306733

[20] Ruan, X. et al. Activin receptor-like kinase 3: a critical modulator of development and function of mineralized tissues. *Front. Cell Dev. Biol.* **11**, 1209817 (2023). 10.3389/fcell.2023.1209817 PMC10347416 37457289

[21] Marino, F. et al. Role of c-kit in myocardial regeneration and aging. *Front. Endocrinol.* **10**, 371 (2019). 10.3389/fendo.2019.00371 PMC6593054 31275242

[22] Di Siena, S. et al. Activated c-Kit receptor in the heart promotes cardiac repair and regeneration after injury. *Cell Death Dis.* **7**, e2317 (2016). 10.1038/cddis.2016.205 PMC4973348 27468693

[23] Cimini, M. et al. C-kit dysfunction impairs myocardial healing after infarction. *Circulation* **116**, I77–I82 (2007). 10.1161/CIRCULATIONAHA.107.708107 17846329

[24] Yaniz-Galende, E. et al. Stem cell factor gene transfer promotes cardiac repair after myocardial infarction via in situ recruitment and expansion of c-kit+ cells. *Circ. Res.* **111**, 1434–1445 (2012). 10.1161/CIRCRESAHA.111.263830 PMC3651889 22931954

[25] Lutz, M. et al. Local injection of stem cell factor (SCF) improves myocardial homing of systemically delivered c-kit + bone marrow-derived stem cells. *Cardiovasc. Res.* **77**, 143–150 (2008). 10.1093/cvr/cvm027 18006465

[26] Takematsu, E. et al. Transmembrane stem cell factor protein therapeutics enhance revascularization in ischemia without mast cell activation. *Nat. Commun.* **13**, 2497 (2022). 10.1038/s41467-022-30103-2 PMC9076913 35523773

[27] Kim, B.-J. et al. Platelet-derived growth factor receptor-alpha positive cardiac progenitor cells derived from multipotent germline stem cells are capable of cardiomyogenesisin vitroandin vivo. *Oncotarget* **8**, 29643–29656 (2017). 10.18632/oncotarget.16772 PMC5444692 28410244

[28] Kalra, K., Eberhard, J., Farbehi, N., Chong, J. J. & Xaymardan, M. Role of PDGF-A/B ligands in cardiac repair after myocardial infarction. *Front. Cell Dev. Biol.* **9**, 669188 (2021). 10.3389/fcell.2021.669188 PMC8424099 34513823

[29] Horikawa, S. et al. PDGFRα plays a crucial role in connective tissue remodeling. *Sci. Rep.* **5**, 17948 (2015). 10.1038/srep17948 PMC4671150 26639755

[30] Ono, C. et al. Upregulated Fcrl5 disrupts B cell anergy and causes autoimmune disease. *Front. Immunol.* **14**, 1276014 (2023). 10.3389/fimmu.2023.1276014 PMC10569490 37841260

[31] Pandey, P. et al. New insights about the PDGF/PDGFR signaling pathway as a promising target to develop cancer therapeutic strategies. *Biomed. Pharmacother.* **161**, 114491 (2023). 10.1016/j.biopha.2023.114491 37002577

[32] Saiz, M. L. et al. Epigenetic targeting of the ACE2 and NRP1 viral receptors limits SARS-CoV-2 infectivity. *Clin. Epigenet.* **13**, 187 (2021). 10.1186/s13148-021-01168-5 PMC8504098 34635175

[33] Rizzolio, S. et al. Neuropilin-1 upregulation elicits adaptive resistance to oncogene-targeted therapies. *J. Clin. Invest.* **128**, 3976–3990 (2018). 10.1172/JCI99257 PMC6118581 29953416

[34] Tang, Y. H. et al. Neuropilin-1 is over-expressed in claudin-low breast cancer and promotes tumor progression through acquisition of stem cell characteristics and RAS/MAPK pathway activation. *Breast Cancer Res.* **24**, 8 (2022). 10.1186/s13058-022-01501-7 PMC8787892 35078508

[35] de la Rosa, G., Corrales-García, L. L., Rodriguez-Ruiz, X., López-Vera, E. & Corzo, G. Short-chain consensus alpha-neurotoxin: a synthetic 60-mer peptide with generic traits and enhanced immunogenic properties. *Amino Acids* **50**, 885–895 (2018). 10.1007/s00726-018-2556-0 29626299

[36] Ensan, D. et al. Targeting ALK2: An open science approach to developing therapeutics for the treatment of diffuse intrinsic pontine glioma. *J. Med. Chem.* **63**, 4978–4996 (2020). 10.1021/acs.jmedchem.0c00395 PMC8213057 32369358

[37] Carvalho, D. et al. ALK2 inhibitors display beneficial effects in preclinical models of ACVR1 mutant diffuse intrinsic pontine glioma. *Commun. Biol.* **2**, 156 (2019). 10.1038/s42003-019-0420-8 PMC6509210 31098401

[38] Zhou, X. P. et al. Germline mutations in BMPR1A/ALK3 cause a subset of cases of juvenile polyposis syndrome and of Cowden and Bannayan-Riley-Ruvalcaba syndromes. *Am. J. Hum. Genet.* **69**, 704–711 (2001). 10.1086/323703 PMC1226057 11536076

[39] Ehata, S. & Miyazono, K. Bone morphogenetic protein signaling in cancer; Some topics in the recent 10 years. *Front. Cell Dev. Biol.* **10**, 883523 (2022). 10.3389/fcell.2022.883523 PMC9174896 35693928

[40] Corless, C. L. et al. PDGFRA mutations in gastrointestinal stromal tumors: frequency, spectrum and in vitro sensitivity to imatinib. *J. Clin. Oncol.* **23**, 5357–5364 (2005). 10.1200/JCO.2005.14.068 15928335

[41] Simpson, J. E. et al. Autophagy supports PDGFRA-dependent brain tumor development by enhancing oncogenic signaling. *Dev. Cell* **59**, 228–243.e7 (2024). 10.1016/j.devcel.2023.11.023 38113891

[42] Jansson, S. et al. The PDGF pathway in breast cancer is linked to tumour aggressiveness, triple-negative subtype and early recurrence. *Breast Cancer Res. Treat.* **169**, 231–241 (2018). 10.1007/s10549-018-4664-7 PMC5945746 29380207

[43] Evans, E. K. et al. A precision therapy against cancers driven by KIT/PDGFRA mutations. *Sci. Transl. Med*. **9**, (2017). 10.1126/scitranslmed.aao1690 29093181

[44] Curtin, J. A., Busam, K., Pinkel, D. & Bastian, B. C. Somatic activation of KIT in distinct subtypes of melanoma. *J. Clin. Oncol.* **24**, 4340–4346 (2006). 10.1200/JCO.2006.06.2984 16908931

[45] Küçükköse, E. et al. KIT promotes tumor stroma formation and counteracts tumor-suppressive TGFβ signaling in colorectal cancer. *Cell Death Dis.* **13**, 617 (2022). 10.1038/s41419-022-05078-z PMC9288482 35842424

[46] Rubin, B. P. et al. KIT activation is a ubiquitous feature of gastrointestinal stromal tumors. *Cancer Res.* **61**, 8118–8121 (2001). 11719439

[47] Sakuma, Y., Sakurai, S., Oguni, S., Hironaka, M. & Saito, K. Alterations of the c-kit gene in testicular germ cell tumors. *Cancer Sci.* **94**, 486–491 (2003). 10.1111/j.1349-7006.2003.tb01470.x PMC11160296 12824871

[48] Hirota, S. et al. Gain-of-function mutations of c-kit in human gastrointestinal stromal tumors. *Science* **279**, 577–580 (1998). 10.1126/science.279.5350.577 9438854

[49] Bryan, C. M. et al. Computational design of a synthetic PD-1 agonist. *Proc. Natl. Acad. Sci. USA* **118**, e2102164118 (2021). 10.1073/pnas.2102164118 PMC8307378 34272285

[50] Edman, N. I. et al. Modulation of FGF pathway signaling and vascular differentiation using designed oligomeric assemblies. *Cell* **187**, 3726–3740.e43 (2024). 10.1016/j.cell.2024.05.025 PMC11246234 38861993

[51] Silva, D.-A. et al. De novo design of potent and selective mimics of IL-2 and IL-15. *Nature* **565**, 186–191 (2019). 10.1038/s41586-018-0830-7 PMC6521699 30626941

[52] Huang, B. et al. Designed endocytosis-inducing proteins degrade targets and amplify signals. *Nature* **638**, 796–804 (2025). 10.1038/s41586-024-07948-2 PMC11839401 39322662

[53] Chevalier, A. et al. Massively parallel de novo protein design for targeted therapeutics. *Nature* **550**, 74–79 (2017). 10.1038/nature23912 PMC5802399 28953867

[54] Jumper, J. et al. Highly accurate protein structure prediction with AlphaFold. *Nature* **596**, 583–589 (2021). 10.1038/s41586-021-03819-2 PMC8371605 34265844

[55] Varadi, M. et al. AlphaFold Protein Structure Database: massively expanding the structural coverage of protein-sequence space with high-accuracy models. *Nucleic Acids Res.* **50**, D439–D444 (2022). 10.1093/nar/gkab1061 PMC8728224 34791371

[56] Varadi, M. et al. AlphaFold Protein Structure Database in 2024: providing structure coverage for over 214 million protein sequences. *Nucleic Acids Res.* **52**, D368–D375 (2024). 10.1093/nar/gkad1011 PMC10767828 37933859

[57] Hinck, A. P. Structural studies of the TGF-βs and their receptors - insights into evolution of the TGF-β superfamily. *FEBS Lett.* **586**, 1860–1870 (2012). 10.1016/j.febslet.2012.05.028 22651914

[58] Lemmon, M. A. & Schlessinger, J. Cell signaling by receptor tyrosine kinases. *Cell* **141**, 1117–1134 (2010). 10.1016/j.cell.2010.06.011 PMC2914105 20602996

[59] Dauparas, J. et al. Robust deep learning-based protein sequence design using ProteinMPNN. *Science* **378**, 49–56 (2022). 10.1126/science.add2187 PMC9997061 36108050

[60] Zhang, Y. & Skolnick, J. TM-align: a protein structure alignment algorithm based on the TM-score. *Nucleic Acids Res.* **33**, 2302–2309 (2005). 10.1093/nar/gki524 PMC1084323 15849316

[61] Gloegl, M. et al. Target-conditioned diffusion generates potent TNFR superfamily antagonists and agonists. *bioRxiv* 10.1101/2024.09.13.612773 (2024). PMC12416549 39636970

[62] Vázquez Torres, S. et al. De novo designed proteins neutralize lethal snake venom toxins. *Nature* **639**, 225–231 (2025). 10.1038/s41586-024-08393-x PMC11882462 39814879

[63] Franco, A. et al. Human Fc receptor-like 5 binds intact IgG via mechanisms distinct from those of Fc receptors. *J. Immunol.* **190**, 5739–5746 (2013). 10.4049/jimmunol.1202860 PMC3660407 23616577

[64] McDonald, I. K. & Thornton, J. M. Satisfying hydrogen bonding potential in proteins. *J. Mol. Biol.* **238**, 777–793 (1994). 10.1006/jmbi.1994.1334 8182748

[65] Lo Conte, L., Chothia, C. & Janin, J. The atomic structure of protein-protein recognition sites. *J. Mol. Biol.* **285**, 2177–2198 (1999). 10.1006/jmbi.1998.2439 9925793

[66] van Kempen, M. et al. Fast and accurate protein structure search with Foldseek. *Nat. Biotechnol.* **42**, 243–246 (2024). 10.1038/s41587-023-01773-0 PMC10869269 37156916

[67] Cheng, P.-N., Pham, J. D. & Nowick, J. S. The supramolecular chemistry of β-sheets. *J. Am. Chem. Soc.* **135**, 5477–5492 (2013). 10.1021/ja3088407 PMC3642101 23548073

[68] Yuzawa, S. et al. Structural basis for activation of the receptor tyrosine kinase KIT by stem cell factor. *Cell* **130**, 323–334 (2007). 10.1016/j.cell.2007.05.055 17662946

[69] Appleton, B. A. et al. Structural studies of neuropilin/antibody complexes provide insights into semaphorin and VEGF binding. *EMBO J.* **26**, 4902–4912 (2007). 10.1038/sj.emboj.7601906 PMC2099469 17989695

[70] Kschonsak, M. et al. Structures of HCMV Trimer reveal the basis for receptor recognition and cell entry. *Cell* **184**, 1232–1244.e16 (2021). 10.1016/j.cell.2021.01.036 33626330

[71] Chennamsetty, N., Voynov, V., Kayser, V., Helk, B. & Trout, B. L. Design of therapeutic proteins with enhanced stability. *Proc. Natl. Acad. Sci. USA* **106**, 11937–11942 (2009). 10.1073/pnas.0904191106 PMC2715526 19571001

[72] Muratspahić, E. et al. De novo design of miniprotein agonists and antagonists targeting G protein-coupled receptors. *bioRxivorg 2025* **03**, .644666 (2025). 23.

[73] Benatuil, L., Perez, J. M., Belk, J. & Hsieh, C.-M. An improved yeast transformation method for the generation of very large human antibody libraries. *Protein Eng. Des. Sel.* **23**, 155–159 (2010). 10.1093/protein/gzq002 20130105

[74] Huang, T. & Hinck, A. P. Production, isolation, and structural analysis of ligands and receptors of the TGF-β superfamily. *Methods Mol. Biol.* **1344**, 63–92 (2016). 10.1007/978-1-4939-2966-5_4 PMC4846357 26520118

[75] Wicky, B. I. M. et al. Hallucinating symmetric protein assemblies. *Science* **378**, 56–61 (2022). 10.1126/science.add1964 PMC9724707 36108048

[76] Kabsch, W. X. D. S. *Acta Crystallogr. D. Biol. Crystallogr.* **66**, 125–132 (2010). 10.1107/S0907444909047337 PMC2815665 20124692

[77] Jamshidiha, M. et al. Coping with strong translational noncrystallographic symmetry and extreme anisotropy in molecular replacement with Phaser: human Rab27a. *Acta Crystallogr. D. Struct. Biol.* **75**, 342–353 (2019). 10.1107/S2059798318017825 PMC6450061 30950405

[78] Tickle, I. STARANISO: use of a WebGL-based 3D interactive graphical display to represent and visualise data quality metrics for anisotropic macromolecular diffraction data. *Acta Crystallogr. A Found. Adv.* **75**, e162 (2019).

[79] Vonrhein, C. et al. Advances in automated data analysis and processing within autoPROC, combined with improved characterisation, mitigation and visualisation of the anisotropy of diffraction limits using STARANISO. *Acta Crystallogr. A Found. Adv.* **74**, a360 (2018).

[80] McCoy, A. J. et al. Phaser crystallographic software. *J. Appl. Crystallogr.* **40**, 658–674 (2007). 10.1107/S0021889807021206 PMC2483472 19461840

[81] Emsley, P., Lohkamp, B., Scott, W. G. & Cowtan, K. Features and development of Coot. *Acta Crystallogr. D. Biol. Crystallogr.* **66**, 486–501 (2010). 10.1107/S0907444910007493 PMC2852313 20383002

[82] Adams, P. D. et al. PHENIX: a comprehensive Python-based system for macromolecular structure solution. *Acta Crystallogr. D. Biol. Crystallogr.* **66**, 213–221 (2010). 10.1107/S0907444909052925 PMC2815670 20124702

[83] Joosten, R. P., Long, F., Murshudov, G. N. & Perrakis, A. The PDB_REDO server for macromolecular structure model optimization. *IUCrJ* **1**, 213–220 (2014). 10.1107/S2052252514009324 PMC4107921 25075342

[84] Ng, P. K.-S. et al. Systematic functional annotation of somatic mutations in cancer. *Cancer Cell* **33**, 450–462.e10 (2018). 10.1016/j.ccell.2018.01.021 PMC5926201 29533785

[85] Mátés, L. et al. Molecular evolution of a novel hyperactive Sleeping Beauty transposase enables robust stable gene transfer in vertebrates. *Nat. Genet.* **41**, 753–761 (2009). 10.1038/ng.343 19412179

