# Supplementary material for: Improved protein binder design using β-pairing targeted RFdiffusion
Source: Nat Commun. 2026 Jan 10;17:1101. doi: 10.1038/s41467-025-67866-3 (PMC12852815; doi:10.1038/s41467-025-67866-3)
Supplement: Supplementary file 1 [file 41467_2025_67866_MOESM1_ESM.pdf]

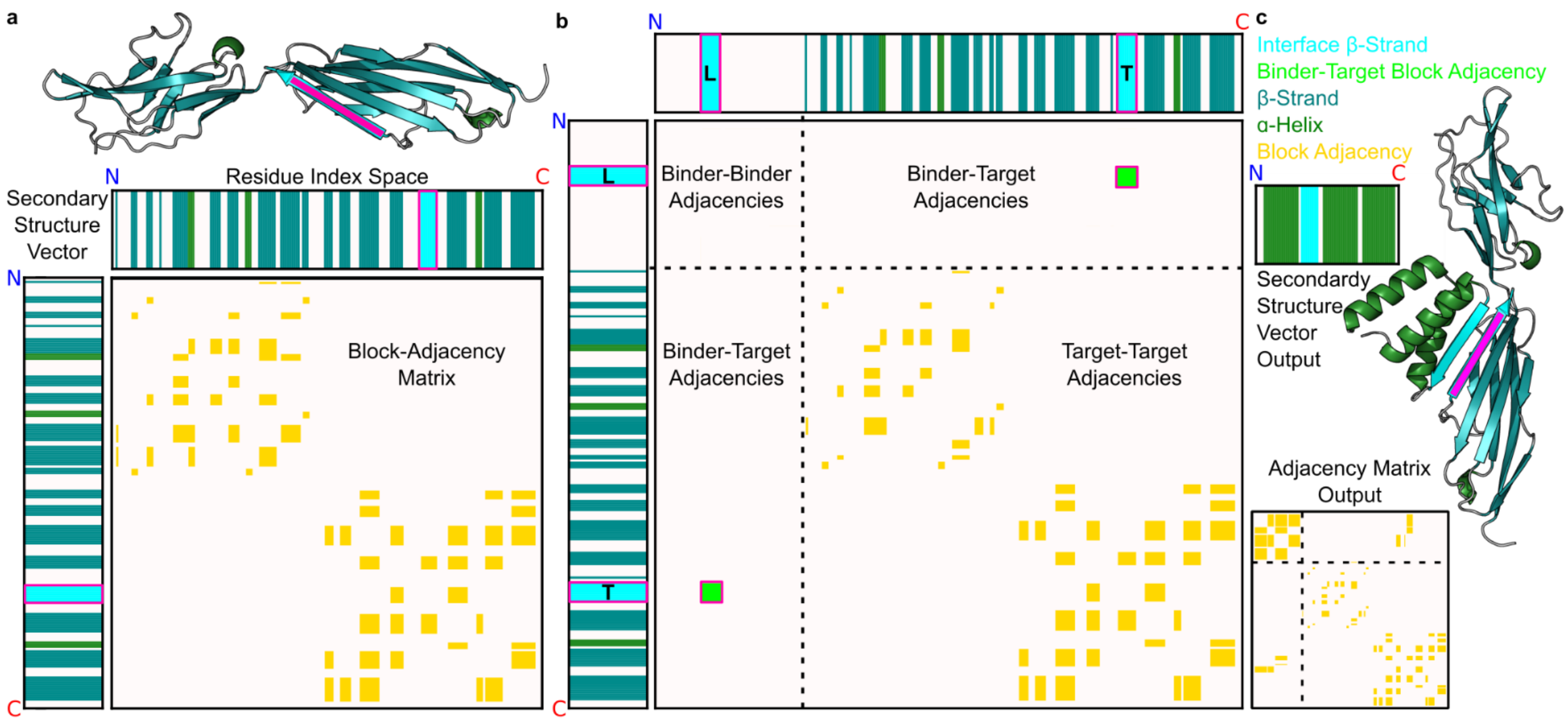

Supplementary Figure 1. *Design of β-strand pairing scaffolds with RFdiffusion.* To leverage the diverse generative potential of RFdiffusion while encouraging β-strand interfaces in design outputs, we implemented an interface conditioning algorithm that generates SS/ADJ conditioning tensors given simple user inputs. The model understands fold conditioning in the form of tensors that label the secondary structures (blue) of each residue (a, top and left), and the adjacencies of these secondary structure blocks (a, yellow center). User specified parameters specify information about the binder-interfacial secondary structure block (a β-strand in this case), the length of this block (b, cyan block in binder tensor L), and the target site residues to which the binder block is adjacent (b, cyan block in target tensor T). From these predefined parameters, the algorithm randomly samples the location of the binder-interfacial secondary structure block in residue index space, while maintaining a defined adjacency (green) to the specified target site residues. This user-defined conditioning tensor guides diffusion outputs towards a β-strand pairing binder-target interface (c). Previously, RFdiffusion interface design calculations could be directed to specific residues specified as target “hotspots” to specify the intended target residues to bind to, this new inter-chain SS/ADJ conditioning enables user indication of “β-strand hotspots" or “α-helical hotspots” during binder scaffold generation. Binder scaffold outputs conditioned on expanded binder-target SS/ADJ tensors enabled design of user-specified β-strand interface types.

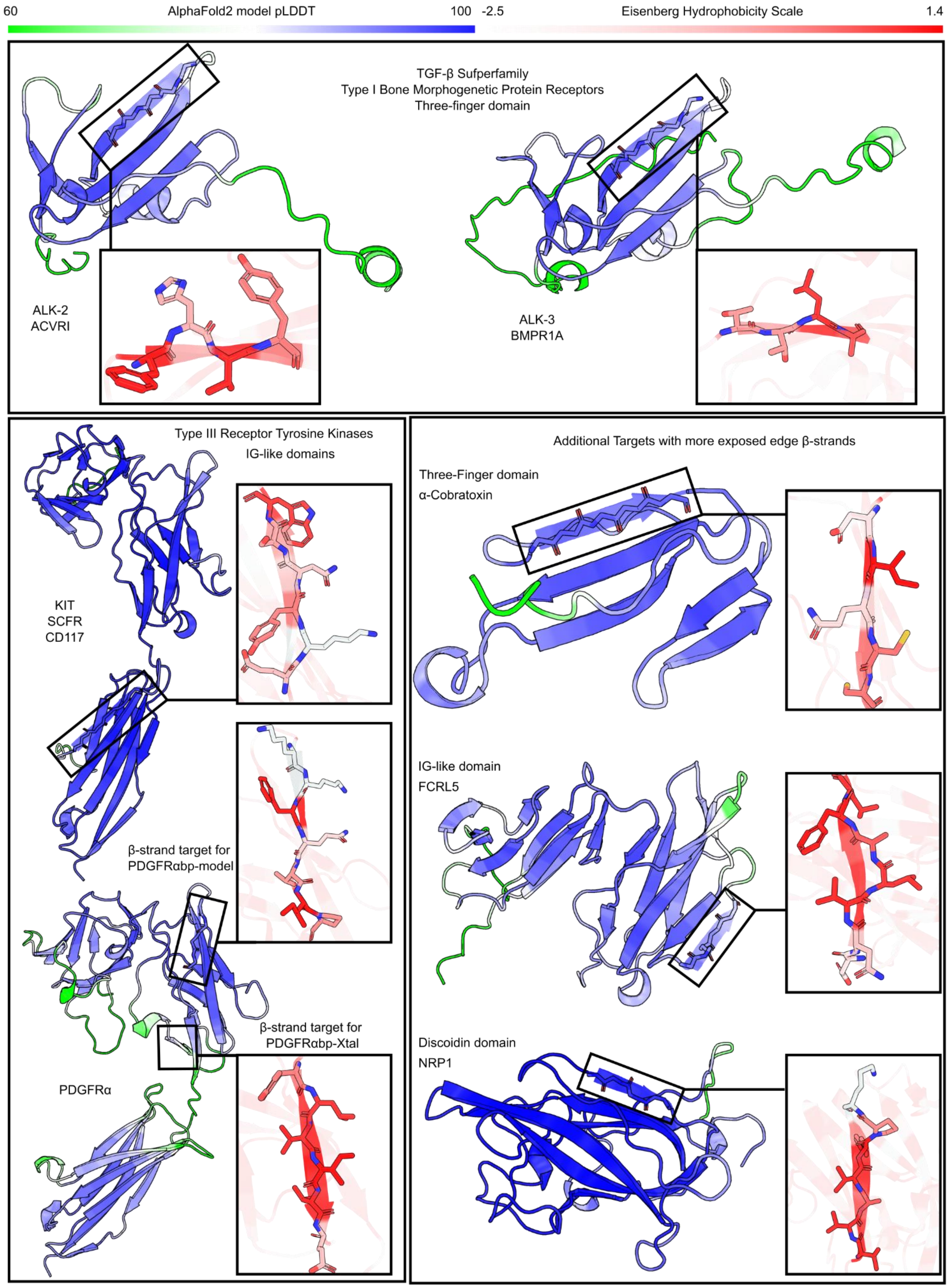
60
AlphaFold2 model pLDDT
100
-2.5
Eisenberg Hydrophobicity Scale
1.4
TGF-β Sufperfamily
Type I Bone Morphogenetic Protein Receptors
Three-finger domain
ALK-2
ACVRI
ALK-3
BMPR1A
Type III Receptor Tyrosine Kinases
IG-like domains
KIT
SCFR
CD117
β-strand target for
PDGFRαbp-model
β-strand target for
PDGFRαbp-Xtal
PDGFRα
Additional Targets with more exposed edge β-strands
Three-Finger domain
α-Cobratoxin
IG-like domain
FCRL5
Discoidin domain
NRP1

1

Supplementary Figure 2. *Target candidates for binder design.* **(a)** Edge β-strands on target proteins as binding sites. In order to test interaction specificity of designed binders, target proteins with similar folds were selected. Three targets (ALK-2, ALK-3, and α-CTX) contain three-finger domains, with the N-terminal edge β-strands targeted for ALK-2 and ALK-3 binders, and the C-terminal edge β-strand targeted for short-chain consensus α-neurotoxin binders. Three other targets (KIT, PDGFRα, and FCRL5) contain immunoglobulin domains. Ig fold N-terminal strands were targeted for KIT and PDGFRα (7LBF) binders, and a non-terminal edge β-strand was targeted on FCRL5. Additionally, we targeted the C-terminal β-strand on PDGFRα (AF2-model) binders. Finally an edge β-strand on a discoidin domain of NRP1 was targeted. AlphaFold 2 models are shown with per-residue pLDDT values ranging from 60 (green) to 100 (blue). High resolution experimentally determined structures (PDB IDs – ALK-2:7YRU, ALK-3:2H62, α-CTX:1CTX, KIT:2E9W, NRP1:2QQI, and PDGFRA:7LBF) of the targeted edge β-strands are shown, with residues labeled by Eisenberg Hydrophobicity Scale (white are more hydrophilic and red are more hydrophobic). No experimental structure exists for FCRL5, and the AlphaFold 2 model edge β-strand is shown here.

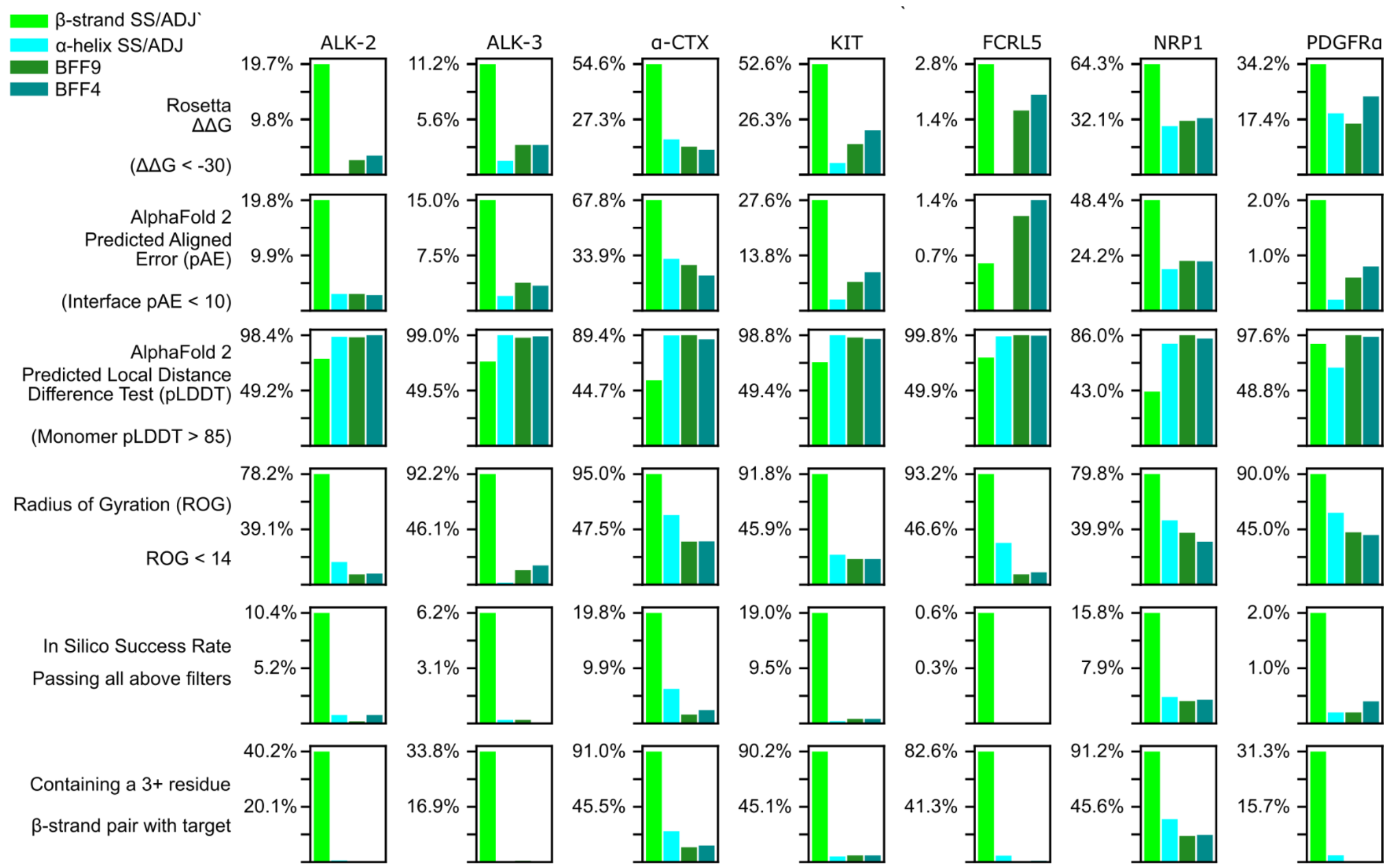

Supplementary Figure 3. *Success rates from different RFdiffusion methods across several predictive in silico metrics.* 40,000 total designs were generated, with equal numbers generated from different RFdiffusion scaffold generation methods across eight target sites. The methods used included hotspot-only conditioning by the default RFdiffusion model (teal, the default for hotspot conditioned binder design), hotspot-only conditioning by a SS/ADJ aware model (dark green, no secondary structure interface conditioning provided), α-helix interface and hotspot conditioning (cyan), and β-strand interface and hotspot conditioning (green). AlphaFold 2 predictions yielded two in silico filtering scores: predicted error among interface residues (interface pAE) and overall confidence in the binder fold (pLDDT). Binders with interface pAE < 10 and binder pLDDT > 85 are considered successful designs with respect to these metrics. Rosetta ΔΔG represents the Rosetta predicted change in energy in the binder designs and targets upon binding. Lower ΔΔG is predictive of higher affinity interactions, with a ΔΔG value of -30 used as a standard of success here. Radius of gyration is a statistic of protein globularity that is predictive of a protein's fold stability. In nature, nearly all 65 amino acid proteins (the size of all designs in this in silico benchmarking set) have a radius of gyration < 14, mostly reflecting known globular folds, such as 3+ α-helix bundles, mixed α/β proteins, and β-sandwich folds. Binders meeting each of these four criteria were considered in silico successes with a high likelihood of experimental success. Additionally, the percentage of outputs exhibiting binder-target β-strand pairing are plotted, although this was not used as a filter for success. In cases where strand pairing binders were generated without strand interface conditioning, binder-target strand pairs tended to contain fewer binder strands and strand pairing interactions. Source data are provided as a Source Data file.

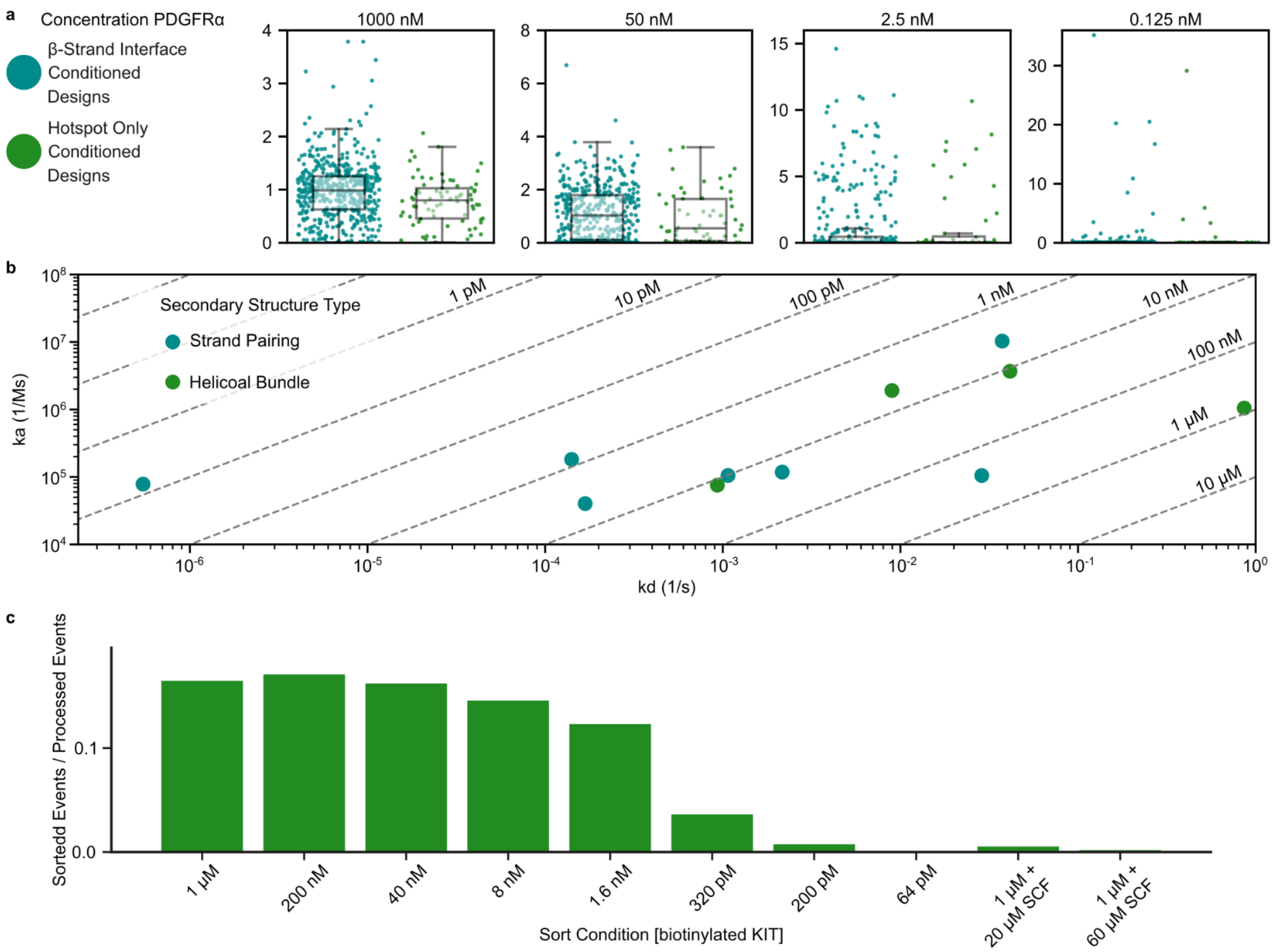

Supplementary Figure 4. *Binding screens suggest preference for strand pairing binders at target sites.* **(a)** β-strand pairing PDGFRα binders are more enriched for target binding in yeast surface display (YSD). Two design approaches, β-strand interface conditioned (N=5616) and hotspot only conditioned (N=10026), were utilized to target PDGFRα. This library containing designs from both approaches were expressed in vectors to display binders extracellularly and with a Myc-tag. Libraries were pooled, sorted for expression by binding to fluorescent Anti-Myc-FITC. The expression positive fraction was initially sorted for PDGFRα binding by incubating cells with biotinylated PDGFRα and Streptavidin phycoerythrin prior to sorting. This parent sort was repeated with titrated PDGFRα concentrations as labeled, and the fold enrichment of each binder in this sort was plotted relative to the NGS counts in the parent sort. **(b)** Affinity kinetics of YSD enriched binders against PDGFRα. The 96 designs that were most enriched during YSD were bacterially expressed and purified for binding measurements against biotinylated PDGFRα via SPR. Binders with KD values > 1μM and exhibiting half the observed maximal SPR response are shown. **(c)** YSD binding data for the KIT binder design library. The binding fraction is represented as the fraction of overall sorting events being FITC (surface expressed binder) and Streptavidin-PE (associated with biotinylated receptor) positive. Lower binding signal in the presence of native ligand confirms the KIT binder library demonstrates the binders largely bind at the targeted Stem Cell Factor (SCF) ligand-binding site. Source data are provided as a Source Data file.

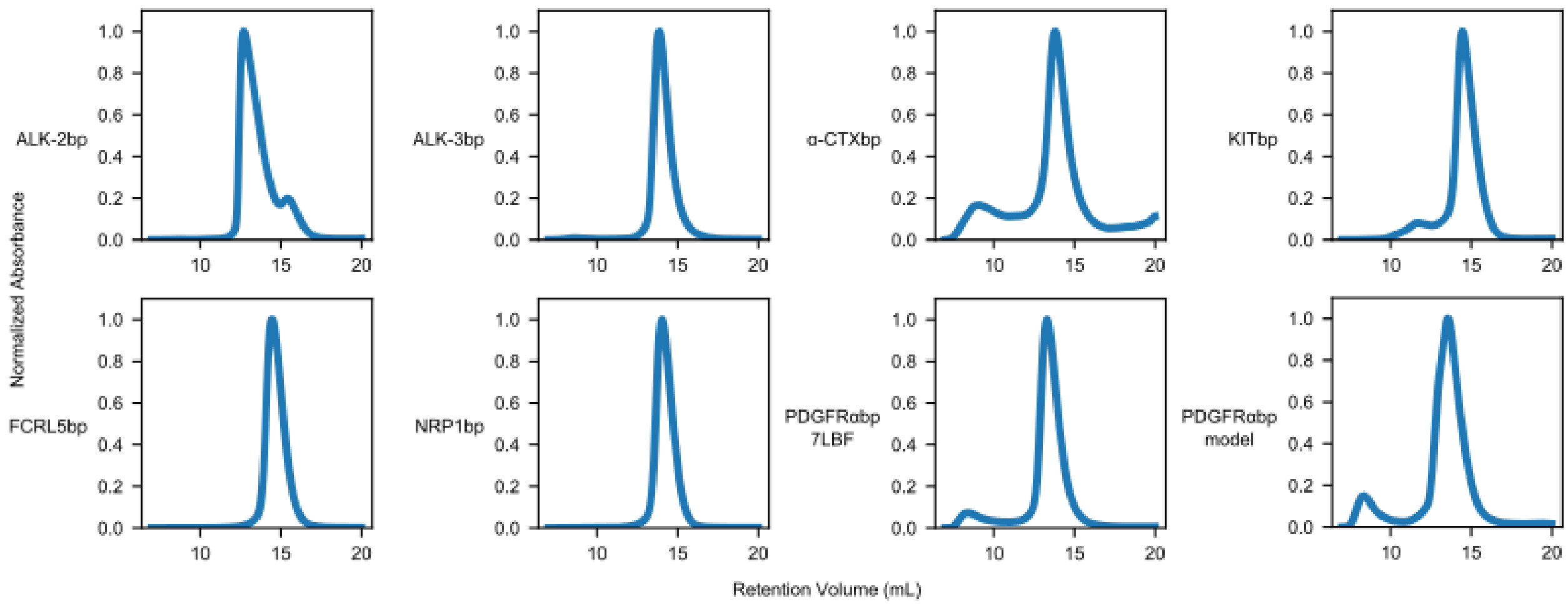

Supplementary Figure 5. *Size-exclusion chromatography purification of β-strand pairing binders shows binders are largely monodisperse.* Each binder was expressed and purified in high yields (>1mg) from 500 mL *E. coli* cultures. Each trace is within the expected range for monomeric species. There is minimal evidence of significant aggregation during purification at room temperature, with aggregate peak areas (<10 mL retention volume) comprising less than 10% of the overall eluate for 7/8 designs. The mg/mL concentrations of collected fractions from purifications were quantified as follows: 1.0 (ALK-2bp), 3.7 (ALK-3bp), 24.352 (α-CTXbp), 1.3 (KITbp), 2.8 (FCRL5bp), 6.5 (NRP1bp), 2.0 (PDGFRαbp-7LBF), and 0.7 (PDGFRα-model). Source data are provided as a Source Data file.

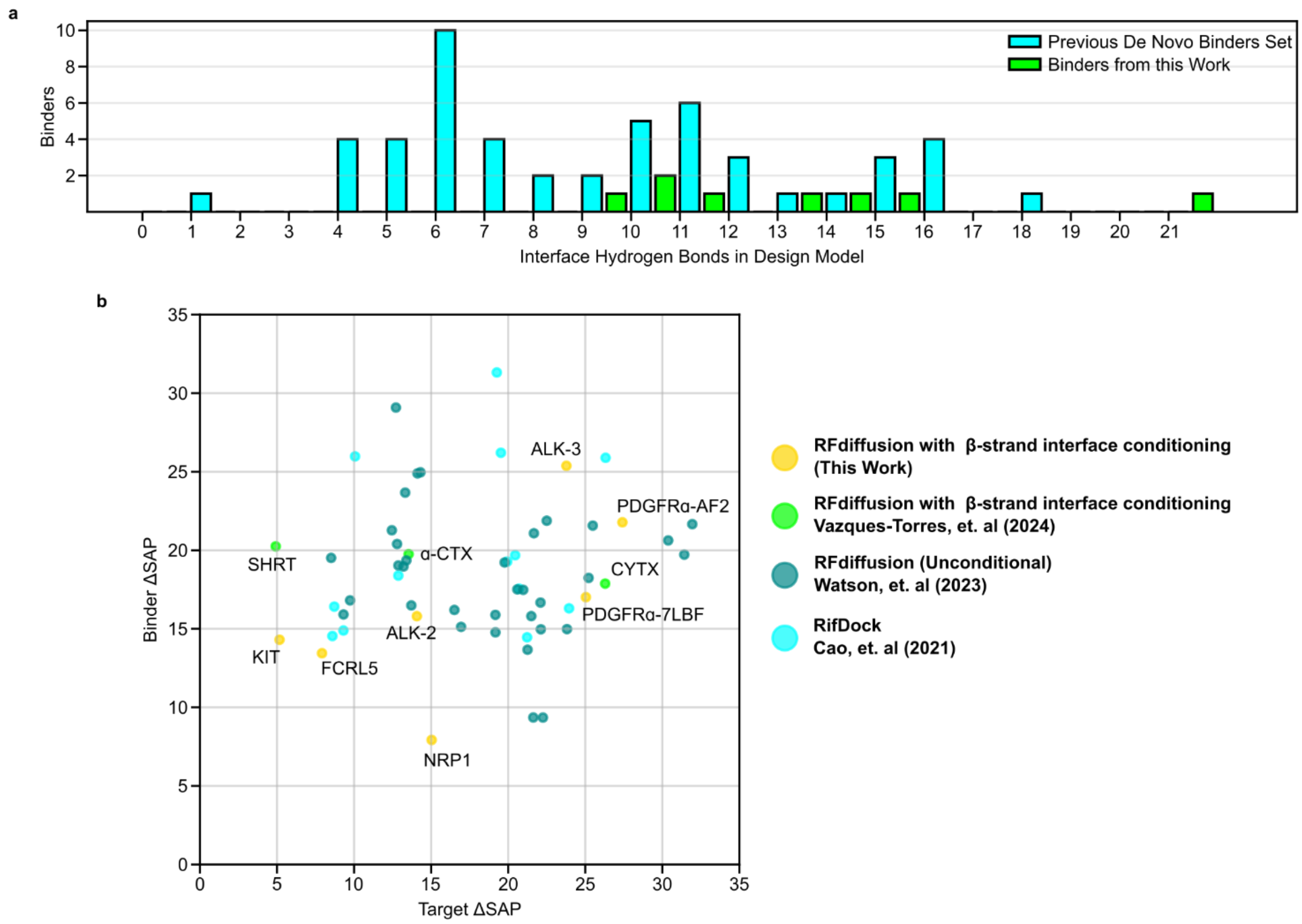

Supplementary Figure 6. *Analysis of strand pairing binder interface polarity in context of previously published de novo binders.* **(a)** Number of hydrogen bonds among 52 binder design models with validated binding data (e.g. biolayer interferometry or SPR). Examples include binders introduced by Cao., et. al. along with several recently developed de novo binders made with deep learning methods base methods (i.e. ProteinMPNN, RFdiffusion, AlphaFold 2). By definition, strand pairing binders form hydrogen bonds with their targets, leading to interfaces with more hydrogen bonds than typical de novo binder interfaces. Additionally, KITbp forms 21 hydrogen bonds with KIT, three more than any previous de novo designed binder, to our knowledge. **(b)** Change in Spatial Aggregation Propensity (ΔSAP) of binders and targets upon complex dissociation. The ΔSAP statistics from binders identified in three previous works are also shown, with 13 binder complexes from Cao, et. al., and 64 from Watson, et. al. The targets for these works include CD3δ, EGFR, FGFR, H3, IGF1R, IL7Ra, Influenza HA, Insulin Receptor, PDGFRβ, PDL1, TGF-β, Tie-2, TrkA, and VirB8. Binders designed with β-strand interface conditioning (three of which were introduced by Vazques-Torres, et. al.) are labeled with their target protein name. The binder footprints for SHRT (Vazques-Torres, et. al.), KIT, and FCRL5 are all more hydrophilic (by SAP score) than any of the sites targeted in these two studies, while KITbp, FCRL5bp, and NRP1bp are all among the least hydrophobic designed binder interfaces from these studies. Source data are provided as a Source Data file.

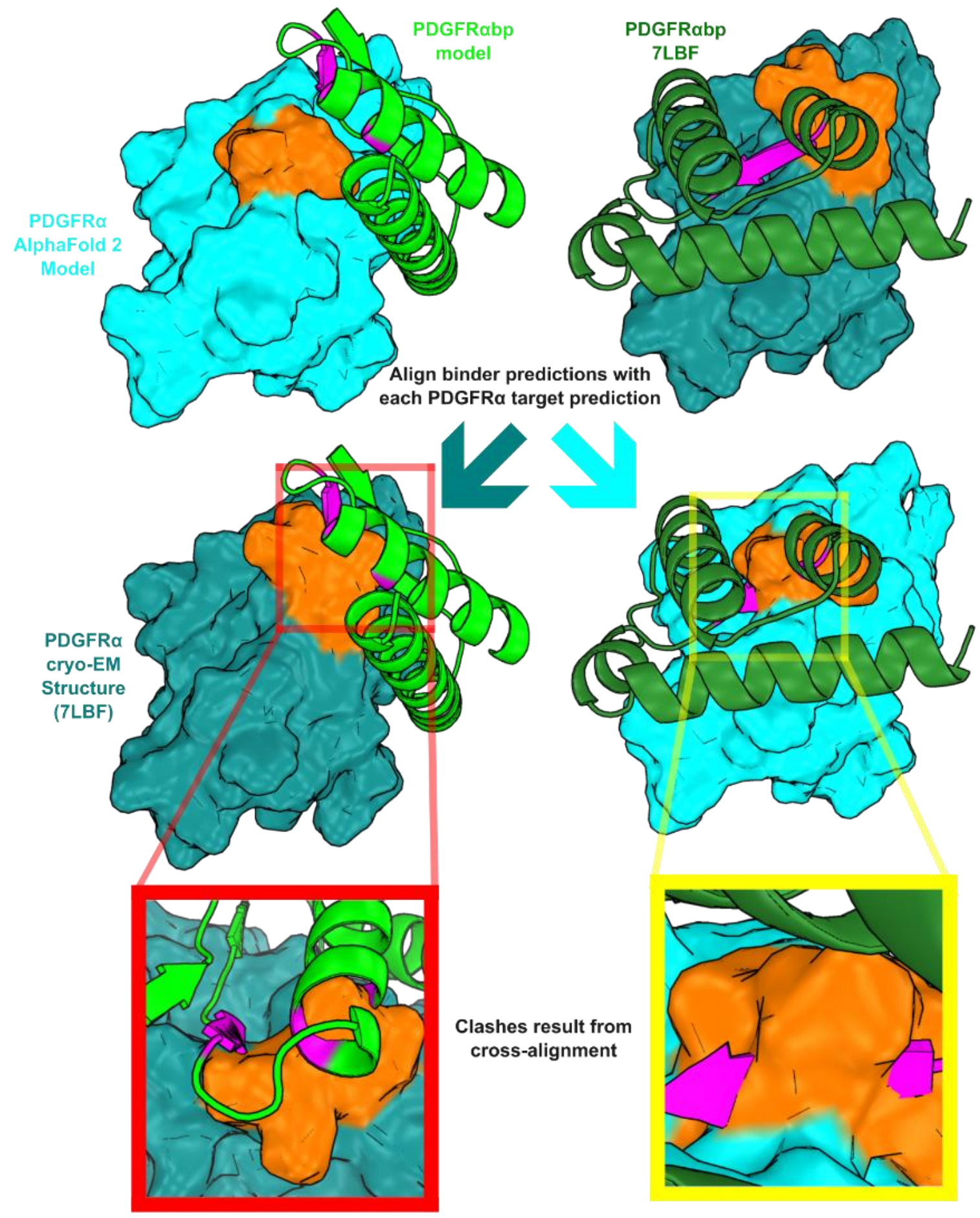

Supplementary Figure 7. *The two PDGFRα binder design models are predicted to induce different PDGFRα conformations.* Our PDGFRαbp-model binder (lime green) designed against the PDGFRα AlphaFold 2 model (cyan) binds to target site residues (orange) in a conformation that significantly varies from the available cryo-EM structure (PDB ID 7LBF, teal). When the AlphaFold 2 model binder (dark green) is aligned to the cryo-EM model, the binder interface clashes with the target site residues (red inset). Similarly, aligning the PDGFRAbp-7LBF binder (dark green) designed against the cryo-EM structure to the AlphaFold 2 model results in a target site clash. Structure agnostic (i.e., only sequences are provided) AlphaFold prediction of each binder in complex with PDGFRA reflects the conformational variability, with the AlphaFold model conformation favored in PDGFRAbp-model predictions while the cryo-EM conformation is favored when predicted with PDGFRAbp-7LBF.

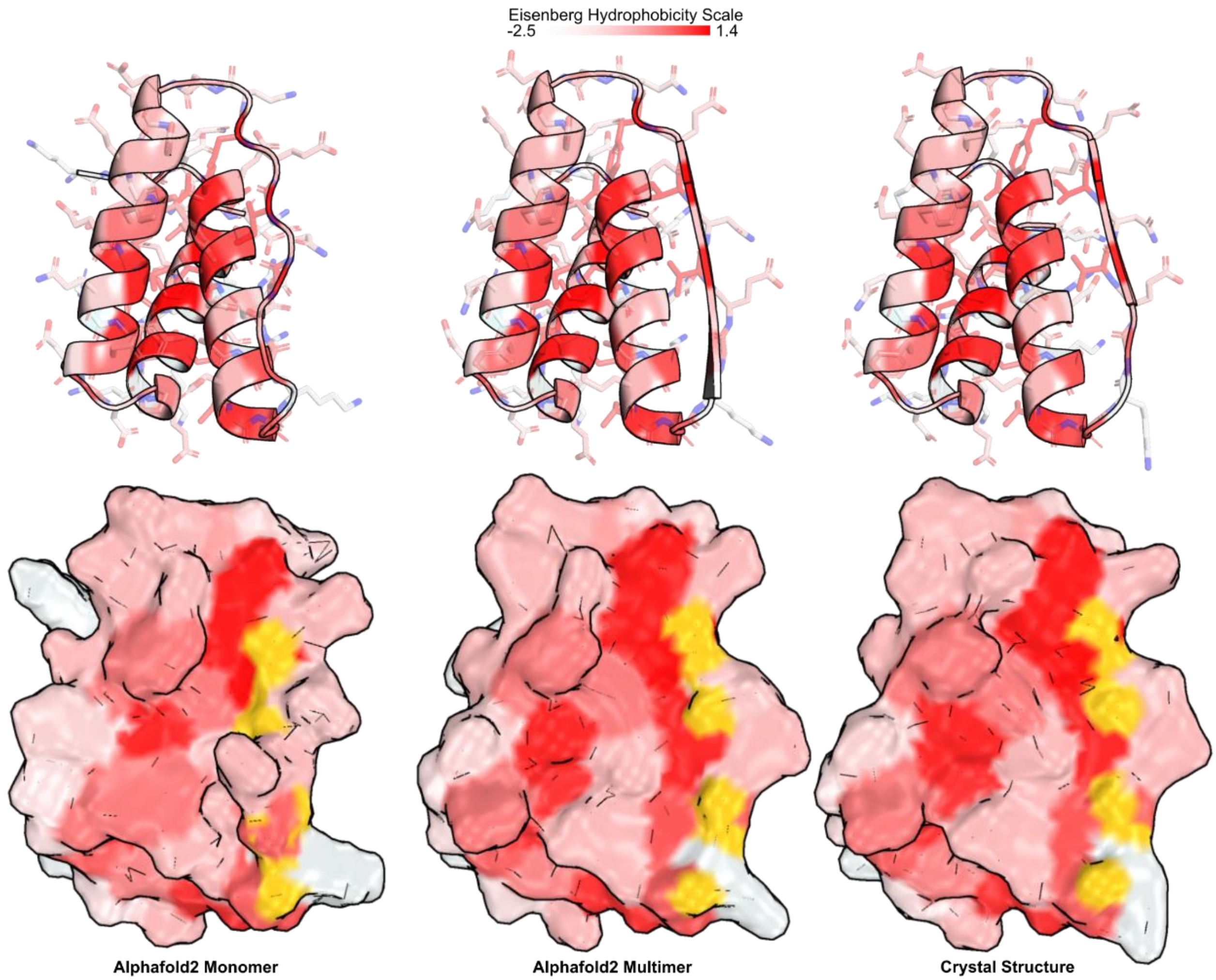

Supplementary Figure 8. *Surface hydrophobicity representations of the KITbp interface for the monomeric (top) KIT-bound (middle) AlphaFold2 predictions of KITbp.* The crystal structure representation (bottom) and the AlphaFold2 multimer prediction display more of KITbp hydrophobic residues than the AlphaFold 2 monomer prediction. The hydrogen bonding atoms (gold) are observed in a β-strand conformation amenable to target β-strand pairing in the KIT-bound conformations, while these same atoms are occluded in the AlphaFold 2 monomer prediction.

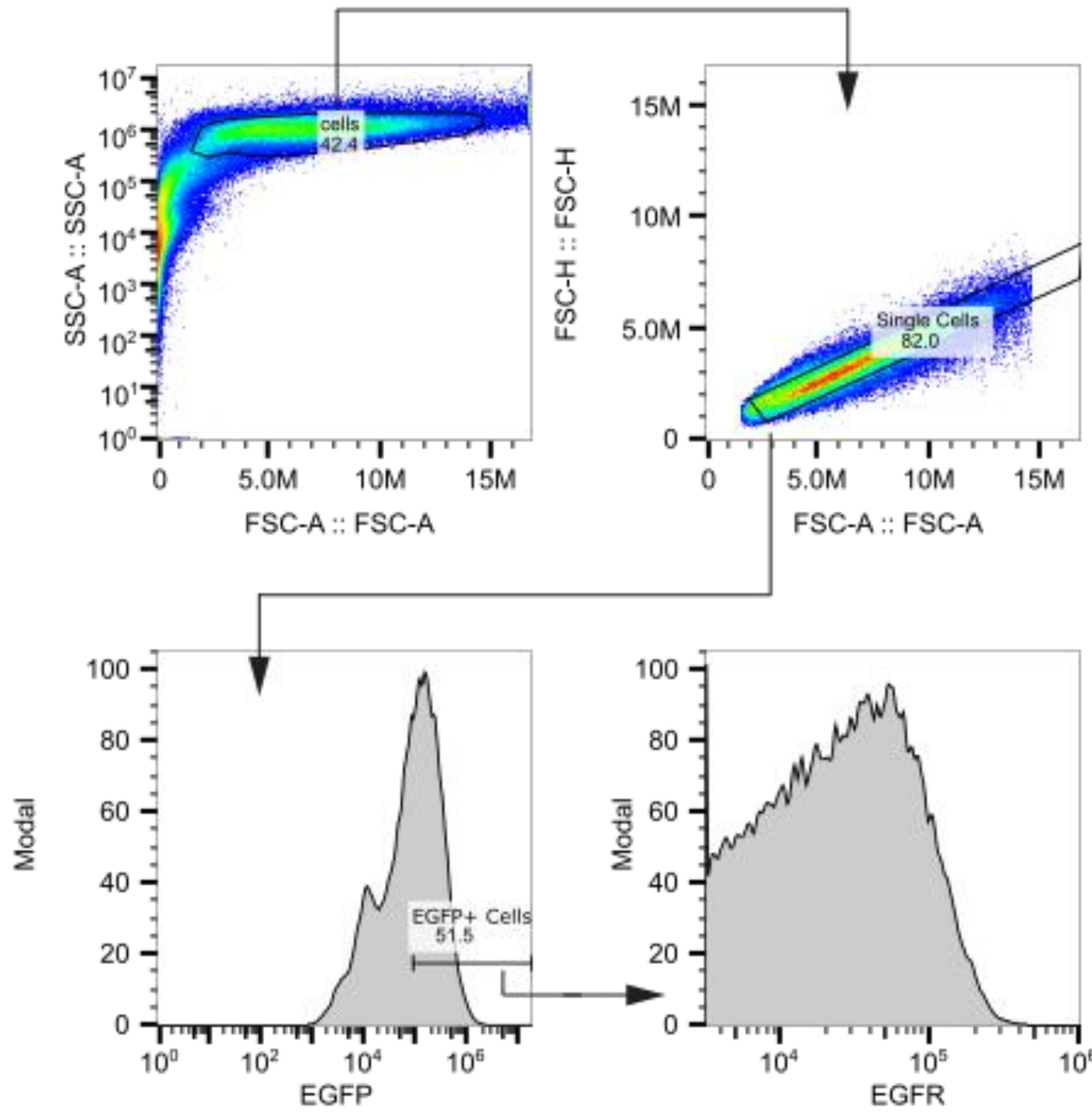

Supplementary Figure 9. *Gating strategy used for quantifying surface EGFR levels*. Gating strategy used for analyzing live HeLa cells with stably integrated Dox-Inducible Flag-FCRL5-T2A-P2A-EGFP for FCRL5+ (EGFP) expression and subsequent analysis of EGFR surface expression.

Library	Entirely Helical Designs	Strand containing Designs	Designs with Strand Interfaces	Parallel Strand Pairing Designs	Antiparallel Strand Pairing Designs	Strand Pairing Binders	Entirely Helical Binders	Top 50 Binders (Strand Pairing)	Top 50 Binders (Entirely Helical)	Top 50 Binders (Parallel Strand Pairing)	Top 50 Binders (Antiparallel Strand Pairing)
ALK-2	4207	5257	5039	18	5018	2	0	2	0	2	0
ALK-3	1735	4263	4196	241	3879	10	0	10	0	0	10
KIT	0	1298	1298	165	1065	1197	0	50	0	7	43
FCRL5	1	4840	4839	421	4236	118	0	50	0	2	48
PDGFRα	9922	5720	5497	2953	2433	73	29	36	14	31	5

Supplementary Table 1. *Strand-pairing binders show improved yeast surface display binding (YSD) success rates.* Binder designs passing in silico filters against each target were ordered and screened for binding by YSD and sorting cells binding to fluorophore complexed target receptors. For ALK-2, ALK-3, and PDGFRα some designs that lacked target strand-pairing interactions passed in silico filters, but were less enriched in the same YSD binding experiments compared to designs with strand-pairing interfaces in the same library. For KIT and FCRL5, designing helical binders with quality in silico metrics was exceedingly difficult. Observed preferences for strand-pair orientation varied for each target, both parallel and antiparallel strand-pairing orientations were observed to show binding signal for three out of five of these targets. Binders are defined as designs with estimated affinities (SC_{50}) less than 1μM. For ALK-2 and ALK-3, we purified and characterized all the binders identified in this screen. From the many KIT, FCRL5, and PDGFRα binders identified by YSD, smaller libraries were selected for individual purification and characterization (N=27, N=12, and N=96, respectively). These designs were selected by ranking designs by calculated YSD SC_{50}, and selecting the top N designs. For PDGFRα, a larger library was considered for testing to better compare strand-pairing and entirely helical binders. Source data are provided as a Source Data file.

**Crystal data**	
Space group	$P2_12_12_1$
Unit cell parameters *a*, *b*, *c* [Å]	41.273, 78.643, 291.008
Unit cell parameters *α*, *β*, *γ* [°]	90, 90, 90
**Data collection**	

Wavelength [Å]	0.9762
Resolution range [Å]	69.18–2.80 (2.90–2.80)
Total reflections	250 107 (37 292)
Unique reflections	24 388 (3 846)
Multiplicity	10.3 (9.7)
Completeness [%]	99.8 (99.4)
Mean I/σ [I]	5.24 (0.69)
Wilson B-factor	51.56
R_{meas} [%]	43.5 (263.9)
$CC_{1/2}$	99.0 (65.2)
**Data statistics for anisotropy-corrected data**	
Resolution range [Å]	69.18–2.80 (2.90–2.80)
Unique reflections	15 541 (626)
Completeness [%]	63.60 (26.31)
Wilson B-factor	34.48
**Refinement statistics for anisotropy-corrected data**	
Reflections used in refinement	15 541 (626)
Reflections used for R-free	735 (42)
R_{work} [%]	22.54 (30.42)
R_{free} [%]	28.24 (36.73)
Number of non-hydrogen atoms	
Total	5 639
Macromolecules	5 472
Ligands	136
Solvent	31
Protein residues	682
RMS (bonds) [Å]	0.001

RMS (angles) [°]	0.40
Ramachandran favored [%]	95.40
Ramachandran allowed [%]	4.60
Ramachandran outliers [%]	0.00
Rotamer outliers [%]	0.16
Clashscore	4.12
B-factors [Å^2]	
Average	48.76
Macromolecules	48.92
Ligands	47.47
Solvent	27.28
Number of TLS groups	11

Supplementary Table 2. *Crystallographic data collection and refinement statistics for the determined structure of the KITbp–KIT123 complex (PDB ID: 9H71).* Statistics for the highest-resolution shell are shown in parentheses.
