# Supplementary material for: Improved protein binder design using β-pairing targeted RFdiffusion
Source: Nat Commun. 2026 Jan 10;17:1101. doi: 10.1038/s41467-025-67866-3 (PMC12852815; doi:10.1038/s41467-025-67866-3)
Supplement: Supplementary file 3 [file 41467_2025_67866_MOESM3_ESM.pdf]

nature portfolio

Peer Review File

Improved protein binder design using β-pairing targeted RFdiffusion

Corresponding Author: Professor David Baker

This file contains all reviewer reports in order by version, followed by all author rebuttals in order by version.

Version 0:

Reviewer comments:

Reviewer #1

(Remarks to the Author)
The main achievement of the paper is the development and application of beta-strand interface-conditioned RFdiffusion. This computational approach generates protein binders specifically tailored to target polar protein surfaces with exposed beta-strands. The method overcomes challenges in designing high-affinity binders for polar protein regions by ensuring precise hydrogen bonding complementarity between the binder and the target's beta-strands. The authors claim that the designed beta sheets show no aggregation due to their customized target recognition. The off-target binding was also tested on the panel of test proteins, and a reasonable level of specificity was found. The work is technically sound, and the results support the conclusions. The method enhances the scope of computational protein design in developing a useful binder with free beta sheet edges. Although the presented data on aggregation-free behavior and specificity is promising, developing a therapeutic protein with a free beta edge would still be risky. Despite my doubts about the possible future applications, I recommend publication in Nat Comms because of the scientific advancement in approaching these challenging targets.
Minor points to address:
1. The CD and size exclusion chromatography data indicate sufficient protein stability. Did the authors use the same protein concentrations in the two types of experiments? Please indicate the concentrations for the size exclusion data. What was the temperature? Cold denaturation can prevent hydrophobic aggregation for beta-sheet structures.
2. For therapeutic applications, it is essential that the designer proteins have long-term stability and do not refold to an amyloid state. Is it possible to run a forced stability test for amyloid formation?
3. Neither the abstract nor the discussion indicates that the methodology relies on yeast display selection as a crucial final step. This can be misleading. It should be pointed out that the conditioned RFDiffusion results in substantial in silico enrichment before the yeast display selection.
4. Please resolve the abbreviations in Fig.1 (BFF4, t-SNE).

(Remarks on code availability)

Reviewer #2

(Remarks to the Author)
Review of "Improved protein binder design using beta-pairing targeted RFdiffusion"

General Assessment:
The manuscript titled "Improved protein binder design using beta-pairing targeted RFdiffusion" presents an advancement in the field of computational protein design. The authors introduced a simple yet effective approach based on previously reported RFDiffusion to designing protein binders that target interfaces with exposed beta-strands with high affinity and specificity. This work holds promise for therapeutic applications, particularly in the development of binders for challenging targets that are often involved in critical biological pathways and diseases. The manuscript is well-structured, with a clear and logical flow of information, and the experimental design is robust and well-reasoned. The use of surface plasmon resonance (SPR) and co-crystal structure analysis to validate the computational models adds a layer of rigor to the study.

Overall, the manuscript is of high quality but would benefit from minor revisions.

Minor comments and suggestions:
1. In the abstract and introduction, the authors describe the problem to be solved as designing binders for "polar protein targets." However, the actual focus of the manuscript is the design of binding proteins for target proteins with exposed beta-strands. While there is some overlap between "polar protein targets" and "exposed beta-strands," these concepts are not synonymous. Exposed beta-strands do not inherently imply that the interfaces are polar. The polarity of the interface is influenced by the side chains of the beta-strands as well. If the target interfaces discussed in the manuscript are indeed polar, it would be helpful for the authors to use figures to better demonstrate this characteristic.

2. The methods section lacks a detailed description of the computational approaches used. Terms such as "tensors" are mentioned throughout the manuscript but are not clearly defined.

3. Section: Results
Subsection: Single Exposed Edge beta-strand: α-CTX, FCRL5, and NRP1
Context: "Binders for α-CTX and NRP1 (1.9 nM and 84 nM respectively) were obtained by testing the 96 designs based solely on in silico metrics."
Comment: A more detailed discussion on why some targets can be addressed through computational metrics alone, while others require experimental library screening, would be beneficial.
4. Section: Results
Subsection: Structural characterization of a KITbp:KIT complex confirms design model accuracy
Context: "Alphafold 2 prediction of the monomeric KITbp without KIT target places the beta-strand pairing residues in a coil conformation that better shields the binder's hydrophobic core residues and hydrogen bonding atoms of the beta-strand interface (Extended data figure 7);"
Comment: Here the author use "Alphafold" rather than "AlphaFold", the capitalization of "AlphaFold 2" should be consistent with the rest of the manuscript.
5. Section: Main Figure 1
Sub-figure: 1c
Context: "(c) Binder design success rates."
Comment: The acronym "BFF4" appears in Figure 1c without prior explanation in the main text. This may cause confusion for readers who are not familiar with the term. To enhance clarity, it would be beneficial to introduce the full term or provide a brief explanation in the main text, even though it is explained in the figure legend.
6. Section: Main Figure 1
Sub-figure: 1d
Context: "(d) Structural clustering of RFdiffusion output binder scaffolds using hotspot conditioning (left) or beta-strand interface conditioning (right) t-SNE transforms of all-by-all pairwise RMSD data among binder scaffolds across all targets are plotted, with close proximity of points representing structural similarity."
Comment: The transformation from RMSD data to t-SNE plots, as shown in Figure 1d, is a critical step in understanding the structural clustering of the binder scaffolds. It would be beneficial to provide a detailed description of this process in the Methods section or the Supplementary Methods. This should include how the all-by-all pairwise RMSD data were calculated and how these distances were transformed into t-SNE coordinates. Such information is essential for readers to fully understand and reproduce the structural analysis presented in the study.
7. Section: Main Figure 2
Sub-figure: 2a
Context: "(a) Design models of binding complexes for each target. Left: Entire complex design models with binders indicated in green/gold and targets in teal/cyan. Beta-strand interface conditioned scaffold design yielded binders with interfacial beta-strands (gold) forming strand-pairing hydrogen bonding interactions with target edge beta-strands (cyan). Middle: Close up view of design model backbone hydrogen-bond interactions with putative hydrogen bonds shown in white. Right: schematic representation of strand pairing interactions showcase the diversity of sequence-independent beta-strand pairing interactions."
Comment: Figure 2a provide detailed views of the hydrogen-bond interactions and schematic representations of the beta-strand pairing interactions, respectively. To enhance the clarity and interpretability of these panels, it would be beneficial to include residue numbers or specific labels to indicate the positions of the target residues involved in the interactions. This would allow readers to more easily identify the key residues contributing to the binding.
8. Section: Main Figure 2
Sub-figure: 2b
Context: "(b) Circular dichroism thermal melts. Full spectrum analyses (left) performed at 25C (cyan), 75C (green), and 95C (gold) assess the overall binder fold at these three temperatures, while single wavelength thermal melts (right) were measured at 217 nM to calculate binder Tm values."
Comment: The notation for temperature in Figure 2b uses "C" (e.g., 25C, 75C, 95C), while the main text uses "°C" (e.g., 25 °C). For consistency, it is recommended to use "°C" throughout the manuscript.

(Remarks on code availability)
A README file is provided with clear instructions on running the script used in this manuscript.

Reviewer #3

(Remarks to the Author)
In this study, the authors developed a workflow for de novo design et subsequent testing of protein binders targeting polar regions of exposed beta-sheets structures. The authors first generated 500 protein scaffolds by conditioning an RFdiffusion

model to specifically target exposed beta-strands of seven proteins. These targets were selected to offer a range of different context and accessibility for the binding partner. For comparison, the same number of scaffolds were generated using a standard RFdiffusion approach. In silico, analysis showed significant improvement in the numbers of candidates meeting quality criteria.
The second part of the paper experimentally evaluated candidate binders for each protein target.
This paper successfully shows the design of binders with high affinity and specificity for each of the protein target investigated. In the experimental section, the text lacks clarity and cohesion, making it difficult to follow exactly what has been tested for each protein target. Although the authors seem to have performed extensive experimental testing of their yeast screen and the successful binders, some of the data is often mentioned with little to no representation to support it. I have detailed my comments below.

Main comments:

1) The number of candidate binders that were experimentally tested and how they were selected is difficult to follow.
As far as I understand, 5,000 candidate binders were tested in silico: ten candidate binder sequences were generated for each of 500 scaffolds, bringing the total to 5,000 potential binders per approach for each target. Subsets were determined to be in silico successes according to AlphaFold 2 and Rosetta metrics, as represented in Figure 1, panel c. Additional criteria ("partial diffusion scaffold optimization and additional sequence sampling") are mentioned in the last paragraph of p.3, and slightly more detailed in the methods section, "computation protein design workflow". However, it is unclear which parameters caused such differences in library size, as high in silico success rates do not seem to be synonymous with larger experimental libraries (e.g. 2.8% of in silico successes and 4,846 library variants for FCRL5, or 52.6% of in silico successes and 1,230 library variants for KIT). In the last paragraph of p.3, 100 to 10^5 variants are said to have been experimentally tested, in the previous paragraph 5,000 variants are said to have been tested in silico whereas the methods section mentions a 10^5 scale. Are the 10^5 variants per target the sum of the variants generated with the two diffusion models? The number of candidates is not stated for ALK-2 and ALK-3. For α-CTX and NRP1, were 96 designs tested for each in total (i.e. 48 each)?
Additional details, for instance a table summarising the cutoffs and resulting library sizes, would make it easier for the reader to understand the selection process.

2) In the last paragraph of p.3, designs are described as being "first screened using yeast surface display". No yeast display results are mentioned for four of the targets (ALK-2, ALK-3, α-CTX and NRP1), although the method section describe the generation of yeast libraries for ALK-2 and ALK-3. Could the authors clarify this point and detail if and why they took the decision to bypass yeast display screening for some targets?
The same paragraph in p.3 suggests that libraries were generated for each targets using both RF diffusion approaches, however data is only shown for one of them in Extended data Figure 4. If this data exists, adding it to the paper would better showcase the reproducibility of these results.

3) The numbers of scaffolds and protein designed for each target and approach, as well as the number of targets are not consistent:
- 6 targets cited in the abstract – although the
- 5,000 candidate binders (p.3) for seven targets
- 40,000 designs for 8 targets (extended data figure 3), with seven targets shown in the figure

4) Throughout the paper, references are made to previous unsuccessful studies which failed to yield an acceptable binder to specific targets. Could the authors clarify if they are discussing unpublished data or specific publications?
- p.3, l.12-16
- p.5, l.4
- p.7, l.35-36

5) This paper clearly demonstrates that the present interface conditioning method can generate high affinity binders to beta-strands regions of proteins. It also shows that a higher number of promising candidates are generated compared to a more common "hotspot" approach. This is demonstrated both in silico (Figure 1) and experimentally (Extended data Figure 4). However, there are no direct comparison about the quality of the binders generated by each approach. The fold-enrichment described in Extended data Figure 4 is dependent on the activity of other library members and could be influenced by protein expression levels. Direct comparison of binding affinity for the most enriched clone(s) from the two RF diffusion approaches would strongly strengthen the claim of this article.

6) Could the authors clarify which yeast binding assay they used to measure binding inhibition of KITbp by SCF (p.9)? It would be helpful to add the data as an extended figure.

7) In Figure 5, panel a, at which timepoint were the photos taken? Showing two timepoints (early and late) would help the reader better visualise the results.

8) In Figure 5, panel b, the plotting of western blot analysis allows an easier comparison of results, and the calculation of the IC50. However, as western blot analysis is highly dependent on the selection of a region of interest, the addition of representative blot images in the extended data would be welcome.

9) Extended data figure 8 is missing (p.11, l.4).

10) On p.14, l.3, the reference for the “previously described protocol’ is missing.

Minor points:

- p.7, l.42: “to bind the a cryo-EM structure”
- p.8, l.9: is the reference meant to be extended data figure 5 and not 4?
- p.11, l.5: “reaching a steady state at in this expression system”

(Remarks on code availability)

Reviewer #4

(Remarks to the Author)
In this manuscript, Sappington et al. show that RFdiffusion can be conditioned to generate designs that bind to a surface exposed beta strand in target protein via a beta strand interface. Success in this area has been challenging for computational binder design protocols, which is frustrating given that many protein targets, particularly cell surface receptors, have surface exposed beta strands.

The authors report details of the development of the approach and show its utility by applying the conditioned RFdiffusion to 8 medically relevant protein targets. They report that in each case, preconditioning RFdiffusion for beta strand interfaces results in favourable designs. They experimentally characterise the designs in a variety of ways, ranging from screening relatively large numbers of candidate designs using yeast display to characterisation or binders using SPR to crystal structures of binder-target complexes to functional assays.

Overall, this is an interesting study that merits publication if the authors address the points below. Additionally, the text was very concise which meant that explanations of methodology, description of results, and the closing discussion felt briefer than they needed to be.

Major points:
- The major concern is that the authors do not clearly differentiate aspects of this study from a previous report by Torres et al., 2025 (ref 64 in the current manuscript; https://doi.org/10.1038/s41586-024-08393-x).

- It seems that in ref 64 the authors report SS/ADJ as a tool to better direct RFdiffusion towards exposed beta-strand interfaces, but in ref 64 SS/ADJ is used at the point of inference (ie on a model that hasn’t been conditioned for SS/ADJ) whereas in the current manuscript SS/ADJ is used to train the model prior to use. This difference should be made much clearer in the text, including early discussion and citation of ref 64, clearer descriptions of both approaches, and better distinction between the two. Moreover, given that SS/ADJ as a concept is not being introduced here (that was done in ref 64), a comparison of the two should be include using one or ideally more of the targets to show benefits, if any, of conditioning the model.

- Page 11: The authors say “In a companion study, α-CTXbp was found to potently neutralize α-CTX, protecting mice from a lethal neurotoxin challenge.” This sentence was surprising as it suggests that the same design has been previously reported in Torres et al., 2025 (https://doi.org/10.1038/s41586-024-08393-x) although it isn’t clear which design is common to both papers. However, the methodology used to design binders in ref 64 and in the current manuscript appear to be different and so it is therefore not really clear whether α-CTXbp was characterized in ref 64 or whether this sentence relates to another design that targets α-CTX. This should be clarified.

Minor points:
- Page 4: ALK-2 and ALK-3 were chosen as targets as they have similar structures which presents a design challenge. However, they only show 30% sequence identity (presumably this value is global), which is low. Extended data figure 2 suggests that, while the beta strands targeted in each protein have some structural similarity with respect to the overall fold, they have very different sequences. Is this really as difficult a challenge as the authors claim?

- Figure 2: The ALK-2bp interacts ALK-2 via parallel beta strands while ALK-3bp interacts with ALK-3 via antiparallel beta strands. This raises a question about the favorability of one configuration vs the other. Was there a preference for anti-parallel or parallel or no preference across the 8 targets? In the case of ALK-2, did all designs interact via a parallel beta strand interface or were both modes sampled and if both modes were present what was the distribution overall and with respect to the scoring metrics (ie did higher scoring designs all interact with the same mode?)

- Figure 2: The terms BFF4 and BFF9 (SFig 3) are not explained

- Page 5: Authors state that previous attempts to make binders to ALK-2, ALK-3 or KIT had failed. Has this been reported anywhere?

- Page 5: The authors say "... the actual binder interaction footprints …" I think the use of "actual" isn't appropriate here as they don't resolve an experimental structure of either complex. They are basically saying that their approach returned different designs.

- Page 7: the authors report that 20% of designs obtained using non conditioned RFdiffusion interacted with the target via a beta-strand interface. How did these designs compare to those generated using the conditioned RFdiffusion protocol? Did they have the same/different interface? Was this parallel or antiparallel? Etc. More details would be helpful.

- Page 8 cites "extended data figure 4" but should this be 5?

- Page 8 - the authors talk about the additional interface contacts involved in design/target binding. It would be useful to know more about the broader interfaces that are generated (e.g. total interface surface area, surface area not related to contacts involving the targeted beta strand, types of interaction involving residues not in the beta strand; are these exclusively hydrophobic?)

- Figure 3: The all-against-all comparison appears to be of limited value given the differences between the structures and compositions of interfaces being targeted. Do the authors have any evidence from the large number of sequences they test that binders that score less well would show reduced specificity and would bind to one or more of the other targets used in the study?

- Page 9: The authors say "The KITbp binding site on KIT overlaps with that of the KIT native ligand stem cell factor (SCF), and consistent with this, saturating concentrations of SCF (20μM) reduced KITbp binding by >99% in a yeast surface binding assay." Which figure does this observation relate to?

- Page 9: The authors say "Lone beta-strands are rare in nature and are found primarily in protein-protein complexes, where a loop in one partner forms a beta-strand conformation that extends the beta-sheet of the other partner." This statement should be supported by references.

- Page 10: The authors say "such KIT-dependent conformational switching may contribute to the observed binding specificity." NMR spectroscopy could be used to evaluate whether KIT exists in multiple conformations in the absence of KITbp and if yes whether any of these conformations preferentially interact with KITbp.

- Figure 4: It would be useful to see density at interface. The FoFc map provided suggests some uncertainty with the side chain position of Trp262 in both copies in the asymmetric unit.

- Page 13: The authors say ""We show that beta-strand interface conditioned RFdiffusion outperforms previous methods for the design of binders against edge beta-strand containing targets." but they do not do direct experimental comparisons of binders generated with and without conditioning. See also the "major" points above.

- Extended data figure 1: missing (a) and (b) labels

- Extended data figure 2: Why not use standard AF pLDDT colour scheme?

- Extended data figure 5: SEC traces are used to support the claim that designs are monodisperse and that "the lack of self association", but no calibration of the columns are provided to show that elution volumes are consistent with monomeric molecular weights. Several traces show evidence of other species, both at shorter and longer elution times. The claim of lack of self association would be strengthened if each sample was run at several concentrations and the elution volume shown to be invariant of loading concentration.

- Extended data figure 7: would help to show secondary structure of structures to help orient reader

- Reference formatting was not appropriate

- Though not my work, the system described in Schwarz-Linek et al (2003; https://doi.org/10.1038/nature01589) might be an interesting test case for the conditioned RFdiffusion approach described here.

(Remarks on code availability)
We looked at the code but did not have time to test it while reviewing the paper; we are planning to do so. We looked at the crystallographic model and density file and associated statistics and these seemed fine though the Rfree is high for a structure of this resolution.

Version 1:

Reviewer comments:

Reviewer #1

(Remarks to the Author)
My concerns and suggestions have been addressed appropriately in the revision. I suggest publication.

(Remarks on code availability)

Reviewer #2

(Remarks to the Author)
The authors have adequately addressed all of my comments and concerns. Publication is recommended.

(Remarks on code availability)

Reviewer #3

(Remarks to the Author)
I am satisfied by the response from the authors. They have provided additional information regarding library design and size which clarified their approach and the work described in the paper. The additional experimental data answered my concerns regarding experimental bias and controls.

I only have some minor comments left as detailed below.

1) In extended data table 1, for PDGFRa multiple libraries are mentioned in the paper:
- At p.7: libraries designed against the cryo-EM structure or the AlphaFold 2 model, made of 3780 and 391 members
- In extended data figure 4: “Two binder libraries, β-strand interface conditioned (N=5616) and hotspot only conditioned (N=5717)”
Are those two different experiments and are the numbers listed in extended data table 1 referring to one or both? Could the authors clarify in the table caption.

2) Different library sizes are cited in the paper’s main text than listed in data table 1. My understanding is that the numbers in extended data table 1 are before selection criteria were applied. Could the authors explicitly state in the caption if that is the case. While non-essential, adding a column in the table to summarise how many designs were experimentally tested (as stated in the main text for some of the targets) would help with clarity and representation of the importance of the selection criteria.

3) p.4: the first part of the sentence “PDGFRα where both helical bundle designs and β-strand pairing designs passed in silico filters, significantly more binders were obtained with the strand directed approach than with standard RFdiffusion at this selection stage (Extended Data Figure 4).” seems incomplete.

4) p.9, typo:
“. the There are 21 sidechain-sidechain”

5) p.12, figure 5
“(b-c) FCRL5 is degraded HeLa-FCRL5 cells by de novo binders”
Did the authors mean that "EGFR" is degraded in HeLa-FCRL5?

(Remarks on code availability)

Reviewer #4

(Remarks to the Author)
Largely speaking, the authors have answered our original questions. Some minor remaining points:

Response 11 – should this be Ext Data Fig 4c?

Response 15 – should this be Ext Data Fig 6?

In the section "β-strand interface conditioning improves binder designs", Sappington et al. state that the authors of the original 2023 RFdiffusion paper tested training with SS/ADJ. Sappington et al. now further supplement this by providing an adjacency matrix when running the RFdiffusion run_inference.py script. We think it would be clearer if Sappington et al. more clearly state that in the text this step is performed at inference, possibly in this sentence:

“We explored whether providing interface conditioning information indicating a binder β-strand pairing with a target edge β-strand at the point of inference could yield designable strand pairing complexes.”

(Remarks on code availability)
Conda env installs fine and code works fine in our hands after the following change:

1) Error in line 82 of make_interface_tensor.py
In our hands, the script creates an invalid path for the .txt file that is needed to feed into the RFdiffusion run_inference.py script:

python make_interface_tensor.py --input_pdb alk2_target.pdb --out_dir /local_home/####/NAT_REVIEW/software/ss_adjacency_tensors --binderlen 65 --target_adj B99-103 --binder_ss E --binder_ss_len 6

Error
sh: 1: cannot create /local_home/####/NAT_REVIEW/software/ss_adjacency_tensors../software.txt: Directory nonexistent

We were able to fix this by changing line 82 in make_interface_tensor.py from

```
os.system(f'echo {name}_adj{"-".join([str(x[0]) for x in adj_set])}_{counter} >> {args.out_dir}../{args.out_dir.split("/")[-2]}.txt')
```

to

```
parent_dir = os.path.dirname(os.path.normpath(args.out_dir))
out_name = os.path.basename(os.path.normpath(args.out_dir)) + ".txt"
outfile = os.path.join(parent_dir, out_name)
os.system(f'echo {name}_adj{"-".join([str(x[0]) for x in adj_set])}_{counter} >> {outfile}')
```

2) Small change to README file
Need to add ‘python’ before the run_inference.py script commands in the third paragraph

Reviewer #5

(Remarks to the Author)
I co-reviewed this manuscript with one of the reviewers who provided the listed reports. This is part of the Nature Communications initiative to facilitate training in peer review and to provide appropriate recognition for Early Career Researchers who co-review manuscripts.

(Remarks on code availability)

Version 2:

Reviewer comments:

Reviewer #3

(Remarks to the Author)
My comments have been addressed and the typos corrected. I recommend publication.

(Remarks on code availability)

Open Access This Peer Review File is licensed under a Creative Commons Attribution 4.0 International License, which permits use, sharing, adaptation, distribution and reproduction in any medium or format, as long as you give appropriate credit to the original author(s) and the source, provide a link to the Creative Commons license, and indicate if changes were made.
In cases where reviewers are anonymous, credit should be given to 'Anonymous Referee' and the source.
The images or other third party material in this Peer Review File are included in the article's Creative Commons license, unless indicated otherwise in a credit line to the material. If material is not included in the article's Creative Commons license and your intended use is not permitted by statutory regulation or exceeds the permitted use, you will need to obtain permission directly from the copyright holder.
To view a copy of this license, visit https://creativecommons.org/licenses/by/4.0/

REVIEWER COMMENTS (**Author responses bolded)**

Reviewer #1 (Remarks to the Author):

The main achievement of the paper is the development and application of beta-strand interface-conditioned RFdiffusion. This computational approach generates protein binders specifically tailored to target polar protein surfaces with exposed beta-strands. The method overcomes challenges in designing high-affinity binders for polar protein regions by ensuring precise hydrogen bonding complementarity between the binder and the target's beta-strands. The authors claim that the designed beta sheets show no aggregation due to their customized target recognition. The off-target binding was also tested on the panel of test proteins, and a reasonable level of specificity was found. The work is technically sound, and the results support the conclusions. The method enhances the scope of computational protein design in developing a useful binder with free beta sheet edges. Although the presented data on aggregation-free behavior and specificity is promising, developing a therapeutic protein with a free beta edge would still be risky. Despite my doubts about the possible future applications, I recommend publication in Nat Comms because of the scientific advancement in approaching these challenging targets. Minor points to address:

1. The CD and size exclusion chromatography data indicate sufficient protein stability. Did the authors use the same protein concentrations in the two types of experiments? Please indicate the concentrations for the size exclusion data. What was the temperature? Cold denaturation can prevent hydrophobic aggregation for beta-sheet structures.

We thank the reviewer for raising this point. The same binder samples were used for all measurements in this study. We have now included the protein concentrations observed during size exclusion chromatography (SEC) in the extended data figure description and clarified that all purifications were conducted at room temperature in the Methods section.

2. For therapeutic applications, it is essential that the designer proteins have long-term stability and do not refold to an amyloid state. Is it possible to run a forced stability test for amyloid formation?

We agree that long-term stability is an important consideration. While CD thermal melts provide a standard assessment of protein stability, we note that for therapeutic development, additional optimizations such as disulfide incorporation or edge-scaffold shielding could be implemented to prevent aggregation. We have included this clarification in the revised manuscript.

3. Neither the abstract nor the discussion indicates that the methodology relies on yeast display selection as a crucial final step. This can be misleading. It should be pointed out that the conditioned RFDiffusion results in substantial in silico enrichment before the yeast display selection.

We thank the reviewer for this suggestion. We have revised the main text to clarify that the interface-conditioned RFdiffusion approach yielded substantial in silico enrichment, and that yeast surface display was used in some, but not all, cases to further improve binder affinity. For instance, binders such as NRP1 and α-CTX were identified without high-throughput enrichment. Extended Data Table 1 was added to give further detail on the role of yeast surface display in enrichment, where it can be seen that our success rates should be high enough to yield binders in lower-throughput contexts, especially with more strict in silico filtering. In two cases (ALK-2 and ALK-3), we likely did need experimental enrichment to yield binders. This lower success rate is acknowledged for these edge cases in the main text.

4. Please resolve the abbreviations in Fig.1 (BFF4, t-SNE).

We have updated the figure to replace the "BFF4" label with default (as this is the colloquial name for the default binder design checkpoint, which is publicly available on the RFDiffusion github) and replaced "t-SNE" with its full term, "t-distributed Stochastic Neighbor Embedding."

Reviewer #2 (Remarks to the Author):

Review of "Improved protein binder design using beta-pairing targeted RFdiffusion"

General Assessment:
The manuscript titled "Improved protein binder design using beta-pairing targeted RFdiffusion" presents an advancement in the field of computational protein design. The authors introduced a simple yet effective approach based on previously reported RFDiffusion to designing protein binders that target interfaces with exposed beta-strands with high affinity and specificity. This work holds promise for therapeutic applications, particularly in the development of binders for challenging targets that are often involved in critical biological pathways and diseases. The manuscript is well-structured, with a clear and logical flow of information, and the experimental design is robust and well-reasoned. The use of surface plasmon resonance (SPR) and co-crystal structure analysis to validate the computational models adds a layer of rigor to the study.

Overall, the manuscript is of high quality but would benefit from minor revisions.

Minor comments and suggestions:

1. In the abstract and introduction, the authors describe the problem to be solved as designing binders for "polar protein targets." However, the actual focus of the manuscript is the design of binding proteins for target proteins with exposed beta-strands. While there is some overlap between "polar protein targets" and "exposed beta-strands," these concepts are not synonymous. Exposed beta-strands do not inherently imply that the interfaces are polar. The polarity of the interface is influenced by the side chains of the beta-strands as well. If the target interfaces discussed in the manuscript are indeed polar, it would be helpful for the authors to use figures to better demonstrate this characteristic.

We appreciate this clarification. We have revised the text to emphasize that exposed beta-strand backbones often present polar atoms, making them a distinct class of polar interfaces. To further support this, we have added a plot (Extended Data Fig. 6) comparing deltaSAP and hydrogen bondering as polarity metrics across our targets and other Baker lab designs. These figures illustrate the trend that our beta-strand pairing binders represent several of the most polar de novo designed binder interfaces we have ever observed.

2. The methods section lacks a detailed description of the computational approaches used. Terms such as “tensors” are mentioned throughout the manuscript but are not clearly defined.

We have clarified the definition and role of interface conditioning tensors in the main text. They are simply arrayed information labeling binder residue secondary structure and target interactions for RFdiffusion.

3. Section: Results
Subsection: Single Exposed Edge beta-strand: α-CTX, FCRL5, and NRP1
Context: "Binders for α-CTX and NRP1 (1.9 nM and 84 nM respectively) were obtained by testing the 96 designs based solely on in silico metrics."
Comment: A more detailed discussion on why some targets can be addressed through computational metrics alone, while others require experimental library screening, would be beneficial.

We have elaborated on the factors influencing experimental library size, noting that design difficulty, target flexibility, and project goals often determined the scale of library testing. Here, we made a conscious choice to order large libraries, as we were not sure of the feasibility of strand-pairing binders, expecting potential difficulties with expression and specificity. As we obtained early results (KIT, PDGFRa, and FCRL5 YSD sorting data) indicating this would not be as much of an issue as expected, we followed up later studies (a-CTX and NRP1) with smaller scale orders to reflect this. However, tested larger libraries for ALK-2 and ALK-3 due to prior binder design campaigns indicating the exceptional challenge presented by this family of targets. We have also added yeast surface display

success rates to extended data table 1 and further clarified the library size rationale in the Methods under the subsection "Production scale computational protein design workflow".

4. Section: Results
Subsection: Structural characterization of a KITbp:KIT complex confirms design model accuracy
Context: "Alphafold 2 prediction of the monomeric KITbp without KIT target places the beta-strand pairing residues in a coil conformation that better shields the binder's hydrophobic core residues and hydrogen bonding atoms of the beta-strand interface (Extended data figure 7);"
Comment: Here the author use "Alphafold" rather than "AlphaFold", the capitalization of "AlphaFold 2" should be consistent with the rest of the manuscript.

Corrected to AlphaFold 2 throughout.

5. Section: Main Figure 1
Sub-figure: 1c
Context: "(c) Binder design success rates."
Comment: The acronym "BFF4" appears in Figure 1c without prior explanation in the main text. This may cause confusion for readers who are not familiar with the term. To enhance clarity, it would be beneficial to introduce the full term or provide a brief explanation in the main text, even though it is explained in the figure legend.

Replaced with "Default." See reviewer 1 comment 4.

6. Section: Main Figure 1
Sub-figure: 1d
Context: "(d) Structural clustering of RFdiffusion output binder scaffolds using hotspot conditioning (left) or beta-strand interface conditioning (right) t-SNE transforms of all-by-all pairwise RMSD data among binder scaffolds across all targets are plotted, with close proximity of points representing structural similarity."
Comment: The transformation from RMSD data to t-SNE plots, as shown in Figure 1d, is a critical step in understanding the structural clustering of the binder scaffolds. It would be beneficial to provide a detailed description of this process in the Methods section or the Supplementary Methods. This should include how the all-by-all pairwise RMSD data were calculated and how these distances were transformed into t-SNE coordinates. Such information is essential for readers to fully understand and reproduce the structural analysis presented in the study.

We have added a detailed description of how TMscore matrices were computed and transformed into t-SNE plots in the Methods section under the subsection "In silico

comparison of RFdiffusion generated scaffolds". Scripts to perform this transformation are included in Additional Files.

7. Section: Main Figure 2
Sub-figure: 2a
Context: "(a) Design models of binding complexes for each target. Left: Entire complex design models with binders indicated in green/gold and targets in teal/cyan. Beta-strand interface conditioned scaffold design yielded binders with interfacial beta-strands (gold) forming strand-pairing hydrogen bonding interactions with target edge beta-strands (cyan). Middle: Close up view of design model backbone hydrogen-bond interactions with putative hydrogen bonds shown in white. Right: schematic representation of strand pairing interactions showcase the diversity of sequence-independent beta-strand pairing interactions."
Comment: Figure 2a provide detailed views of the hydrogen-bond interactions and schematic representations of the beta-strand pairing interactions, respectively. To enhance the clarity and interpretability of these panels, it would be beneficial to include residue numbers or specific labels to indicate the positions of the target residues involved in the interactions. This would allow readers to more easily identify the key residues contributing to the binding.

Residue numbers and identifiers have been added to the structural figures in 2a to aid interpretation.

8. Section: Main Figure 2
Sub-figure: 2b
Context: "(b) Circular dichroism thermal melts. Full spectrum analyses (left) performed at 25C (cyan), 75C (green), and 95C (gold) assess the overall binder fold at these three temperatures, while single wavelength thermal melts (right) were measured at 217 nM to calculate binder Tm values."
Comment: The notation for temperature in Figure 2b uses "C" (e.g., 25C, 75C, 95C), while the main text uses "℃" (e.g., 25 ℃). For consistency, it is recommended to use "℃" throughout the manuscript.

We now use "°C" consistently throughout the manuscript.

Reviewer #2 (Remarks on code availability):

A README file is provided with clear instructions on running the script used in this manuscript.

Reviewer #3 (Remarks to the Author):

In this study, the authors developed a workflow for de novo design et subsequent testing of protein binders targeting polar regions of exposed beta-sheets structures. The authors first generated 500 protein scaffolds by conditioning an RFdiffusion model to specifically target exposed beta-strands of seven proteins. These targets were selected to offer a range of different context and accessibility for the binding partner. For comparison, the same number of scaffolds were generated using a standard RFdiffusion approach. In silico, analysis showed significant improvement in the numbers of candidates meeting quality criteria.
The second part of the paper experimentally evaluated candidate binders for each protein target.
This paper successfully shows the design of binders with high affinity and specificity for each of the protein target investigated. In the experimental section, the text lacks clarity and cohesion, making it difficult to follow exactly what has been tested for each protein target. Although the authors seem to have performed extensive experimental testing of their yeast screen and the successful binders, some of the data is often mentioned with little to no representation to support it. I have detailed my comments below.

Main comments:

1) The number of candidate binders that were experimentally tested and how they were selected is difficult to follow.
As far as I understand, 5,000 candidate binders were tested in silico: ten candidate binder sequences were generated for each of 500 scaffolds, bringing the total to 5,000 potential binders per approach for each target. Subsets were determined to be in silico successes according to AlphaFold 2 and Rosetta metrics, as represented in Figure 1, panel c. Additional criteria ("partial diffusion scaffold optimization and additional sequence sampling") are mentioned in the last paragraph of p.3, and slightly more detailed in the methods section, "computation protein design workflow". However, it is unclear which parameters caused such differences in library size, as high in silico success rates do not seem to be synonymous with larger experimental libraries (e.g. 2.8% of in silico successes and 4,846 library variants for FCRL5, or 52.6% of in silico successes and 1,230 library variants for KIT). In the last paragraph of p.3, 100 to 10^5 variants are said to have been experimentally tested, in the previous paragraph 5,000 variants are said to have been tested in silico whereas the methods section mentions a 10^5 scale. Are the 10^5 variants per target the sum of the variants generated with the two diffusion models?
The number of candidates is not stated for ALK-2 and ALK-3. For α-CTX and NRP1, were 96 designs tested for each in total (i.e. 48 each)?
Additional details, for instance a table summarising the cutoffs and resulting library sizes, would make it easier for the reader to understand the selection process.

Additional clarification was provided throughout the manuscript to clearly define and differentiate our in silico experiments from our larger design campaigns for testing in the lab. See reviewer 1 comment 3 and reveiwer 2 comment 3 for additional comments on library sizes.

> 2) In the last paragraph of p.3, designs are described as being "first screened using yeast surface display". No yeast display results are mentioned for four of the targets (ALK-2, ALK-3, α-CTX and NRP1), although the method section describe the generation of yeast libraries for ALK-2 and ALK-3. Could the authors clarify this point and detail if and why they took the decision to bypass yeast display screening for some targets?
> The same paragraph in p.3 suggests that libraries were generated for each targets using both RF diffusion approaches, however data is only shown for one of them in Extended data Figure 4. If this data exists, adding it to the paper would better showcase the reproducibility of these results.

See reviewer 1 comment 3 and reveiwer 2 comment 3 for additional comments on library sizes. Please find Extended Data Table 1, which presents statistics on the secondary structures represented in our YSD libraries along with their respective success rates in our binding assay.

> 3) The numbers of scaffolds and protein designed for each target and approach, as well as the number of targets are not consistent:
> - 6 targets cited in the abstract – although the
> - 5,000 candidate binders (p.3) for seven targets
> - 40,000 designs for 8 targets (extended data figure 3), with seven targets shown in the figure

We have ensured that references to the number of targets (six, seven, or eight) are now consistent throughout the manuscript and figures. Note that there are eight binder against seven targets, with two binders presented for PDGFRα. Thank you for your attention to this detail.

> 4) Throughout the paper, references are made to previous unsuccessful studies which failed to yield an acceptable binder to specific targets. Could the authors clarify if they are discussing unpublished data or specific publications?
> - p.3, l.12-16
> - p.5, l.4
> - p.7, l.35-36

We have unpublished data on failed binder design campaigns. Finding this data generated by lab alumni can be difficult, but we were able to recover sequences

that failed to bind for ALK-3 and KIT, which we have included in this submission in Additional Files.

> 5) This paper clearly demonstrates that the present interface conditioning method can generate high affinity binders to beta-strands regions of proteins. It also shows that a higher number of promising candidates are generated compared to a more common “hotspot” approach. This is demonstrated both in silico (Figure 1) and experimentally (Extended data Figure 4). However, there are no direct comparison about the quality of the binders generated by each approach. The fold-enrichment described in Extended data Figure 4 is dependent on the activity of other library members and could be influenced by protein expression levels. Direct comparison of binding affinity for the most enriched clone(s) from the two RF diffusion approaches would strongly strengthen the claim of this article.

This is an important consideration. Although we attempted design of both helical and strand pairing binders for these targets, in each case strand pairing binders were vastly overrepresented among our in silico successes. In some cases (e.g., KIT, FCRL5, and NRP1), we were unable to generate entirely helical in silico successes. When this was possible for other targets (ALK-2, ALK-3, PDGFRa), we indeed ordered and tested the helical designs alongside the strand-pairing binders. We only identified potential helical binders for PDGFRa that were tested by SPR alongside the strand pairing designs. We now include experimental data comparing conditioned and unconditioned designs that were tested side-by-side by yeast surface display and SPR (extended data figure 4 and extended data table 1).

6) Could the authors clarify which yeast binding assay they used to measure binding inhibition of KITbp by SCF (p.9)? It would be helpful to add the data as an extended figure.

We have added an extended data figure showing the KITbp–SCF competition assay. The method is presented in the manuscript, and no changes were made for the competition assay besides adding ligand as described.

> 7) In Figure 5, panel a, at which timepoint were the photos taken? Showing two timepoints (early and late) would help the reader better visualise the results.

We include a mention of the timepoint for the photos in the figure legend.

> 8) In Figure 5, panel b, the plotting of western blot analysis allows an easier comparison of results, and the calculation of the IC50. However, as western blot analysis is highly dependent on the selection of a region of interest, the addition of representative blot images in the extended data would be welcome.

Representative blots have been included in the Figure for visualization.

9) Extended data figure 8 is missing (p.11, l.4).

We have corrected the figure reference.

10) On p.14, l.3, the reference for the “previously described protocol’ is missing.

Citation has been added.

Minor points:

- - p.7, l.42: “to bind the a cryo-EM structure”
- - p.8, l.9: is the reference meant to be extended data figure 5 and not 4?
- - p.11, l.5: “reaching a steady state at in this expression system”

All corrected.

Reviewer #4 (Remarks to the Author):

In this manuscript, Sappington et al. show that RFdiffusion can be conditioned to generate designs that bind to a surface exposed beta strand in target protein via a beta strand interface. Success in this area has been challenging for computational binder design protocols, which is frustrating given that many protein targets, particularly cell surface receptors, have surface exposed beta strands.

The authors report details of the development of the approach and show its utility by applying the conditioned RFdiffusion to 8 medically relevant protein targets. They report that in each case, preconditioning RFdiffusion for beta strand interfaces results in favourable designs. They experimentally characterise the designs in a variety of ways, ranging from screening relatively large numbers of candidate designs using yeast display to characterisation or binders using SPR to crystal structures of binder-target complexes to functional assays.

Overall, this is an interesting study that merits publication if the authors address the points below. Additionally, the text was very concise which meant that explanations of methodology, description of results, and the closing discussion felt briefer than they needed to be.

Major points:
- The major concern is that the authors do not clearly differentiate aspects of this study from a previous report by Torres et al., 2025 (ref 64 in the current manuscript; https://doi.org/10.1038/s41586-024-08393-x).

The immediate impact of that binder rapid experimental progress resulted in that being published earlier than this. Here, we describe that method in more detail,

provide code for implementation, and demonstrate the generalizability of edge strand pairing for binder design.

- It seems that in ref 64 the authors report SS/ADJ as a tool to better direct RFdiffusion towards exposed beta-strand interfaces, but in ref 64 SS/ADJ is used at the point of inference (ie on a model that hasn't been conditioned for SS/ADJ) whereas in the current manuscript SS/ADJ is used to train the model prior to use. This difference should be made much clearer in the text, including early discussion and citation of ref 64, clearer descriptions of both approaches, and better distinction between the two. Moreover, given that SS/ADJ as a concept is not being introduced here (that was done in ref 64), a comparison of the two should be include using one or ideally more of the targets to show benefits, if any, of conditioning the model.

This difference is incorrect. The binder presented here is the same as the binder introduced by Susana's paper, and designed using the same model and methods described.

- Page 11: The authors say "In a companion study, α-CTXbp was found to potently neutralize α-CTX, protecting mice from a lethal neurotoxin challenge." This sentence was surprising as it suggests that the same design has been previously reported in Torres et al., 2025 (https://doi.org/10.1038/s41586-024-08393-x) although it isn't clear which design is common to both papers. However, the methodology used to design binders in ref 64 and in the current manuscript appear to be different and so it is therefore not really clear whether α-CTXbp was characterized in ref 64 or whether this sentence relates to another design that targets α-CTX. This should be clarified.

We include a citation of Susana's paper earlier in the text (as soon as α-CTXbp is introduced), as well as clarification that this is the same binder described there.

Minor points:

- Page 4: ALK-2 and ALK-3 were chosen as targets as they have similar structures which presents a design challenge. However, they only show 30% sequence identity (presumably this value is global), which is low. Extended data figure 2 suggests that, while the beta strands targeted in each protein have some structural similarity with respect to the overall fold, they have very different sequences. Is this really as difficult a challenge as the authors claim?

We have toned down the claim regarding design difficulty and now emphasize backbone similarity despite low sequence identity. Our presumption was that the inherently side-chain independent interactions of strand-pairs might lead to promiscuous interactions between proteins with similar edge strands. These targets, having nearly identical edge strand backbones, provide at least one example to test this hypothesis.

- Figure 2: The ALK-2bp interacts ALK-2 via parallel beta strands while ALK-3bp interacts with ALK-3 via antiparallel beta strands. This raises a question about the favorability of one configuration vs the other. Was there a preference for anti-parallel or parallel or no preference across the 8 targets? In the case of ALK-2, did all designs interact via a parallel beta strand interface or were both modes sampled and if both modes were present what was the distribution overall and with respect to the scoring metrics (ie did higher scoring designs all interact with the same mode?)

Extended data table 1 includes data on the relative success rates of parallel and antiparallel designs, which seemed to be target dependent.

- Figure 2: The terms BFF4 and BFF9 (SFig 3) are not explained

Removed from all figures. See reviewer 1 comment 4.

- Page 5: Authors state that previous attempts to make binders to ALK-2, ALK-3 or KIT had failed. Has this been reported anywhere?

No, but we have included the sequences of these failed designs in Additional Files. See reviewer 3 comment 4.

- Page 5: The authors say "... the actual binder interaction footprints …" I think the use of "actual" isn't appropriate here as they don't resolve an experimental structure of either complex. They are basically saying that their approach returned different designs.

Agreed it is not correct to say "actual". Toned language appropriately.

- Page 7: the authors report that 20% of designs obtained using non conditioned RFdiffusion interacted with the target via a beta-strand interface. How did these designs compare to those generated using the conditioned RFdiffusion protocol? Did they have the same/different interface? Was this parallel or antiparallel? Etc. More details would be helpful.

We did not observe any statistical differences between strand pairing binders generated with or without conditioning. For NRP1, there was perhaps a slight preference for antiparallel strand pairs among unconditional designs (57 out of 112 strand pairing designs were antiparallel). This compares with (352 out of 482 for conditioned outputs). Overall, there seemed to be a preference for antiparallel pairing (1640 out of 2179 strand pairs). While experimental data indicated that both

parallel and antiparallel strand pairing can yield binders. See reviewer 3 comment 5.

- Page 8 cites “extended data figure 4” but should this be 5?

Fixed.

- Page 8 - the authors talk about the additional interface contacts involved in design/target binding. It would be useful to know more about the broader interfaces that are generated (e.g. total interface surface area, surface area not related to contacts involving the targeted beta strand, types of interaction involving residues not in the beta strand; are these exclusively hydrophobic?)

We added calculations of total interface surface area and composition, now discussed in the main text.

- Figure 3: The all-against-all comparison appears to be of limited value given the differences between the structures and compositions of interfaces being targeted. Do the authors have any evidence from the large number of sequences they test that binders that score less well would show reduced specificity and would bind to one or more of the other targets used in the study?

Typically, spatial aggregation propensity (SAP), determined by the degree of exposed hydrophobic surface area, is used to estimate a binder’s propensity for aggregation and non-specific binding. These binders were filtered on this metric, but the polar backbone groups of edge strand interfaces made us concerned that the binders would nonspecifically bind other edge strands without exhibiting high SAP scores. This specificity experiment was performed to address this hypothesis. Additional experimentation would be needed to fully characterize off-target binding against a proteome for a proper analysis, and we hope to do so in future publications that directly address this topic.

- Page 9: The authors say “The KITbp binding site on KIT overlaps with that of the KIT native ligand stem cell factor (SCF), and consistent with this, saturating concentrations of SCF (20μM) reduced KITbp binding by >99% in a yeast surface binding assay.” Which figure does this observation relate to?

Now included in extended data figure 6a.

- Page 9: The authors say “Lone beta-strands are rare in nature and are found primarily in protein-protein complexes, where a loop in one partner forms a beta-strand conformation that extends the beta-sheet of the other partner.” This statement should be supported by references.

We revised the sentence and acknowledged the limited literature while clarifying our reasoning.

> - Page 10: The authors say “such KIT-dependent conformational switching may contribute to the observed binding specificity.” NMR spectroscopy could be used to evaluate whether KIT exists in multiple conformations in the absence of KITbp and if yes whether any of these conformations preferentially interact with KITbp.

While NMR spectroscopy would be the gold-standard for evaluation here, our primary aim is to demonstrate the ability of beta-strand interface-conditioned RFdiffusion to design specific binders. The predicted flexibility of KITbp may reflect a form of metastability not observed in previously designed binders, but this was an incidental finding rather than a predefined focus of our work. As such, we did not pursue a detailed biophysical characterization, which we believe falls outside the scope of this study.

> - Figure 4: It would be useful to see density at interface. The FoFc map provided suggests some uncertainty with the side chain position of Trp262 in both copies in the asymmetric unit.

We are not entirely clear on the suggested uncertainty at Trp262. The reviewer might potentially refer to two unmodelled 'blobs' around this residue – see the screenshot below where Fo-Fc is visualized by red and green colors (green means there is some extra density in data which is not modelled while red means there is some structure modelled in the place where there is no experimental density). However, these two green blobs are just some solvent molecules that are typical for many of the deposited structures and are also present across our whole structure and not only here. We modelled some of these blobs (water molecules, co-solvent molecules, etc.) but at this resolution, not everything can be reliably modelled to prevent data overfitting.
We are not entirely clear on the suggested uncertainty at Trp262. The reviewer might potentially refer to two unmodelled 'blobs' around this residue – see the screenshot below where Fo-Fc is visualized by red and green colors (green means there is some extra density in data which is not modelled while red means there is some structure modelled in the place where there is no experimental density). However, these two green blobs are just some solvent molecules that are typical for many of the deposited structures and are also present across our whole structure and not only here. We modelled some of these blobs (water molecules, co-solvent molecules, etc.) but at this resolution, not everything can be reliably modelled to prevent data overfitting.

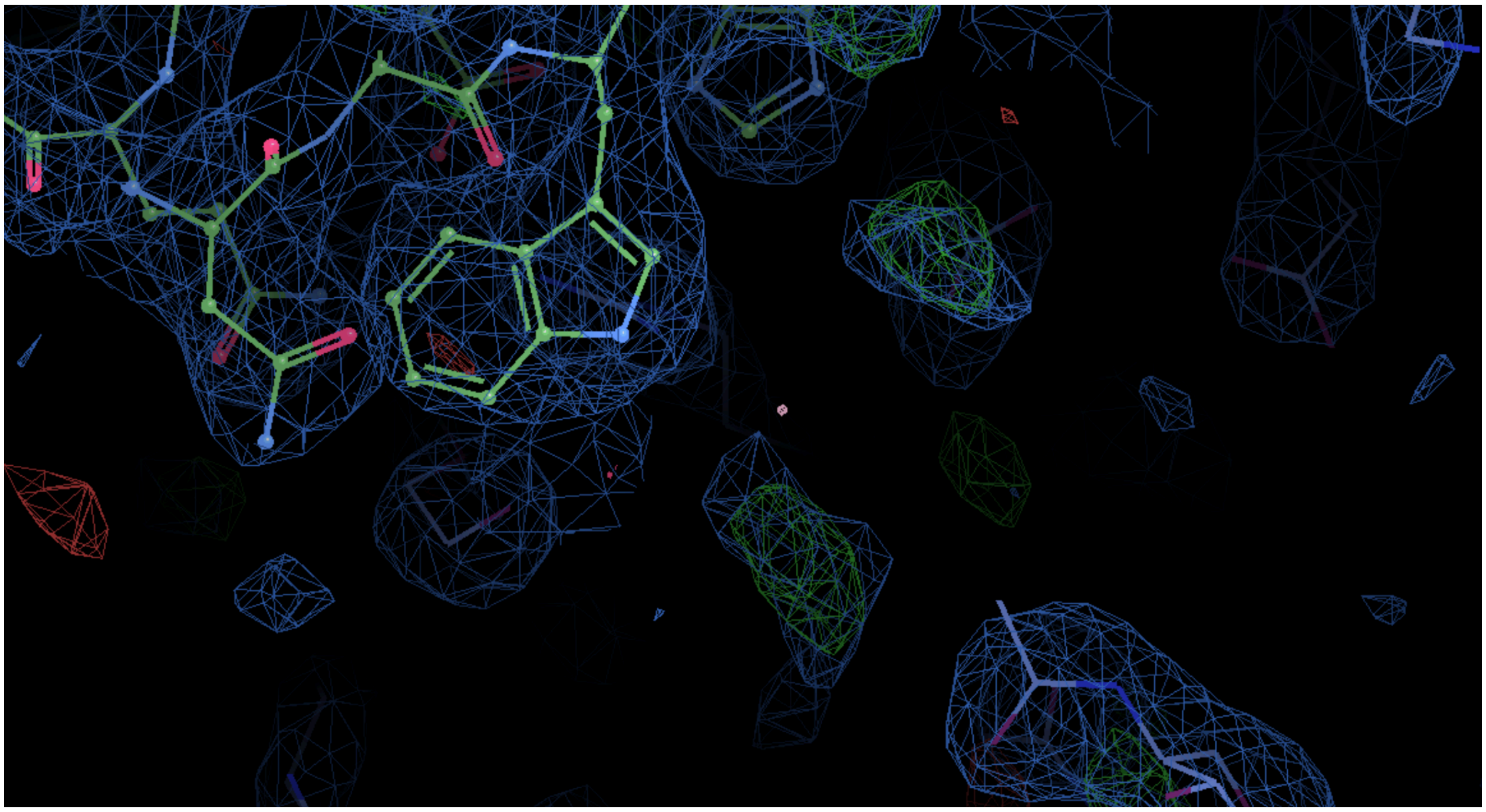

- Page 13: The authors say “”We show that beta-strand interface conditioned RFdiffusion outperforms previous methods for the design of binders against edge beta-strand containing targets.” but they do not do direct experimental comparisons of binders generated with and without conditioning. See also the “major” points above.

We now qualify this claim and note that additional side-by-side experimental comparisons are shown in the extended data figure 5 and table 1.

Extended data figure 1: missing (a) and (b) labels

Corrected figure labeling

- Extended data figure 2: Why not use standard AF pLDDT colour scheme?

We were not aware of the extent that the blue, yellow, and orange pLDDT color scheme was standard at the time of making this figure. The depicted scale is how the figure author visualizes pLDDT as it gives slightly more per residue granularity than the 4-binned standard scheme.

- Extended data figure 5: SEC traces are used to support the claim that designs are monodisperse and that “the lack of self association”, but no calibration of the columns are provided to show that elution volumes are consistent with monomeric molecular weights. Several traces show evidence of other species, both at shorter

and longer elution times. The claim of lack of self association would be strengthened if each sample was run at several concentrations and the elution volume shown to be invariant of loading concentration.

We have adjusted the wording to clarify that SEC traces suggest monodispersity but do not confirm monomeric status without additional validation. We instead state that we did not not observe significant aggregation during any of our experiments.

- Extended data figure 7: would help to show secondary structure of structures to help orient reader

Secondary structure representations were added to what is now extended data figure 8.

- Reference formatting was not appropriate

All references have been reformatted to comply with journal standards.

- Though not my work, the system described in Schwarz-Linek et al (2003; https://doi.org/10.1038/nature01589) might be an interesting test case for the conditioned RFdiffusion approach described here.

We thank the reviewer for this suggestion and agree that this system could be a compelling future test case for our approach.

Reviewer #4 (Remarks on code availability):

We looked at the code but did not have time to test it while reviewing the paper; we are planning to do so. We looked at the crystallographic model and density file and associated statistics and these seemed fine though the Rfree is high for a structure of this resolution.

Point-by-Point Response to Reviewers

Reviewer #1:

1. CD and SEC protein concentrations and temperatures
We thank the reviewer for raising this point. The same binder samples were used for all measurements in this study. We have now included the protein concentrations observed during size exclusion chromatography (SEC) in the extended data figure description and clarified that all purifications were conducted at room temperature in the Methods section.

2. Stability testing and amyloid aggregation
We agree that long-term stability is an important consideration. While CD thermal melts provide a standard assessment of protein stability, we note that for therapeutic development, additional optimizations such as disulfide incorporation or edge-scaffold shielding could be implemented to prevent aggregation. We have included this clarification in the revised manuscript.

3. Clarification of yeast display's role in enrichment
We thank the reviewer for this suggestion. We have revised the main text to clarify that the interface-conditioned RFdiffusion approach yielded substantial in silico enrichment, and that yeast surface display was used in some, but not all, cases to further improve binder affinity. For instance, binders such as NRP1 and α-CTX were identified without high-throughput enrichment. Extended Data Table 1 was added to give further detail on the role of yeast surface display in enrichment, where it can be seen that our success rates should be high enough to yield binders in lower-throughput contexts, especially with more strict in silico filtering. In two cases (ALK-2 and ALK-3), we likely did need experimental enrichment to yield binders. This lower success rate is acknowledged for these edge cases in the main text.

4. Abbreviations in Fig. 1
We have updated the figure to replace the "BFF4" label with default (as this is the colloquial name for the default binder design checkpoint, which is publicly available on the RFDiffusion github) and replaced "t-SNE" with its full term, "t-distributed Stochastic Neighbor Embedding."

Reviewer #2:

1. Clarification of "polar surfaces" vs. "exposed beta-strands"
We appreciate this clarification. We have revised the text to emphasize that exposed beta-strand backbones often present polar atoms, making them a distinct class of polar interfaces. To further support this, we have added a plot (Extended Data Fig. 6) comparing deltaSAP and hydrogen bondering as polarity metrics across our targets and other Baker lab designs. These figures illustrate the trend that our beta-strand pairing binders represent several of the most polar de novo designed binder interfaces we have ever observed.

2. Definition of computational terms (e.g., "tensor")

We have clarified the definition and role of interface conditioning tensors in the Methods section.

3. Variation in in silico vs. experimental success
We have elaborated on the factors influencing experimental library size, noting that design difficulty, target flexibility, and project goals often determined the scale of library testing. Here, we made a conscious choice to order large libraries, as we were not sure of the feasibility of strand-pairing binders, expecting potential difficulties with expression and specificity. As we obtained early results (KIT, PDGFRa, and FCRL5 YSD sorting data) indicating this would not be as much of an issue as expected, we followed up later studies (a-CTX and NRP1) with smaller scale orders to reflect this. However, tested larger libraries for ALK-2 and ALK-3 due to prior binder design campaigns indicating the exceptional challenge presented by this family of targets. We have also added yeast surface display success rates to extended data table 1 and further clarified the library size rationale in the Methods under the subsection “Production scale computational protein design workflow”.

4. Capitalization consistency of AlphaFold
Corrected throughout.

5. BFF4 label in Fig. 1c
Replaced with “Default.” See reviewer 1 comment 4.

6. Clarification of t-SNE methodology in Fig. 1d
We have added a detailed description of how TMscore matrices were computed and transformed into t-SNE plots in the Methods section under the subsection “In silico comparison of RFdiffusion generated scaffolds”. Scripts to perform this transformation are included in Additional Files.

7. Residue labels in Fig. 2a
Residue numbers and identifiers have been added to the structural figures to aid interpretation.

8. Temperature notation consistency in Fig. 2b
We now use “°C” consistently throughout the manuscript.

Reviewer #3:

1. Clarification of library sizes and selection criteria
Additional clarification was provided throughout the manuscript to clearly define and differentiate our in silico experiments from our larger design campaigns for testing in the lab. See reviewer 1 comment 3 and reveiwer 2 comment 3 for additional comments on library sizes.

2. Explanation of yeast display use across targets
See reviewer 1 comment 3 and reveiwer 2 comment 3 for additional comments on library sizes. Please find Extended Data Table 1, which presents statistics on the secondary structures

represented in our YSD libraries along with their respective success rates in our binding assay.

3. Target count consistency
We have ensured that references to the number of targets (six, seven, or eight) are now consistent throughout the manuscript and figures. Note that there are eight binder against seven targets, with two binders presented for PDGFRα. Thank you for your attention to this detail.

4. Clarify whether prior failed binders were unpublished or published
We have unpublished data on failed binder design campaigns. Finding this data generated by lab alumni can be difficult, but we were able to recover sequences that failed to bind for ALK-3 and KIT, which we have included in this submission in Additional Files.

5. Direct comparison of conditioned vs unconditioned binder affinity
This is an important consideration. Although we attempted design of both helical and strand pairing binders for these targets, in each case strand pairing binders were vastly overrepresented among our in silico successes. In some cases (e.g., KIT, FCRL5, and NRP1), we were unable to generate entirely helical in silico successes. When this was possible for other targets (ALK-2, ALK-3, PDGFRa), we indeed ordered and tested the helical designs alongside the strand-pairing binders. We only identified potential helical binders for PDGFRa that were tested by SPR alongside the strand pairing designs. We now include experimental data comparing conditioned and unconditioned designs that were tested side-by-side by yeast surface display and SPR (extended data figure 4 and extended data table 1).

6. Clarification of yeast assay and competition data
We have added an extended data figure showing the KITbp–SCF competition assay. The method is presented in the manuscript, and no changes were made for the competition assay besides adding ligand as described.

7. Timepoints in Figure 5a
We include a mention of the timepoint for the photos in the figure legend.

8. Representative western blot images
Representative blots have been included in the Figure for visualization.

9. Missing Extended Data Fig. 8
We have corrected the figure reference.

10. Missing citation on protocol
Citation has been added.

Reviewer #4:

1. Clarification of overlap with Torres et al. (2025)

This difference is incorrect. The binder presented here is the same as the binder introduced by Susana's paper, and designed using the same model and methods described. The immediate impact of that binder rapid experimental progress resulted in that being published earlier than this. Here, we describe that method, demonstrate its generalizability, and make it more available.

2. Clarification of α-CTXbp overlap between studies
We include a citation of Susana's paper earlier in the text, as well as clarification that this is the same binder described there.

3. Design challenge of ALK-2 and ALK-3
We have toned down the claim regarding design difficulty and now emphasize backbone similarity despite low sequence identity. Our presumption was that the inherently side-chain independent interactions of strand-pairs might lead to promiscuous interactions between proteins with similar edge strands. These targets, having nearly identical edge strand backbones, provide at least one example to test this hypothesis.

4. Parallel vs antiparallel strand pairing preference
Extended data table 1 includes data on the relative success rates of parallel and antiparallel designs, which seemed to be target dependent.

5. Unexplained BFF4/BFF9 labels
Removed from all figures.

6. Evidence for previous failed designs
No, but we have included the sequences of these failed designs in Additional Files. See reviewer 3 comment 4.

7. Comparison of non-strand-pairing vs. strand-pairing RFdiffusion designs
We did not observe any statistical differences between strand pairing binders generated with or without conditioning. For NRP1, there was perhaps a slight preference for antiparallel strand pairs among unconditional designs (57 out of 112 strand pairing designs were antiparallel). This compares with (352 out of 482 for conditioned outputs). Overall, there seemed to be a preference for antiparallel pairing (1640 out of 2179 strand pairs). While experimental data indicated that both parallel and antiparallel strand pairing can yield binders. See reviewer 3 comment 5.

8. Clarification of Figure reference (extended data fig. 5)
Fixed.

9. Interface contact analysis and surface area
We added calculations of total interface surface area and composition, now discussed in the main text.

10. Specificity across targets
Typically, spatial aggregation propensity (SAP) is used to estimate a binder's propensity for aggregation and non-specific binding. These binders were filtered on this metric, but the polar backbone groups of edge strand interfaces made us concerned that the binders would nonspecifically bind other edge strands without exhibiting high SAP scores. This specificity experiment was performed to address this hypothesis. Additional experimentation would be needed to fully characterize off-target binding against a proteome for a proper analysis, and we hope to do so in future publications that directly address this topic.

11. KITbp competition figure
Now included in extended data figure 6a.

12. Statement on lone beta-strands
We revised the sentence and acknowledged the limited literature while clarifying our reasoning.

13. KIT conformational states
While NMR spectroscopy would be the gold-standard for evaluation here, our primary aim is to demonstrate the ability of beta-strand interface-conditioned RFdiffusion to design specific binders. The predicted flexibility of KITbp may reflect a form of metastability not observed in previously designed binders, but this was an incidental finding rather than a predefined focus of our work. As such, we did not pursue a detailed biophysical characterization, which we believe falls outside the scope of this study.

14. Density at interface (Figure 4)
We are not entirely clear on the suggested uncertainty at Trp262. The reviewer might potentially refer to two unmodelled 'blobs' around this residue – see the screenshot below where Fo-Fc is visualized by red and green colors (green means there is some extra density in data which is not modelled while red means there is some structure modelled in the place where there is no experimental density). However, these two green blobs are just some solvent molecules that are typical for many of the deposited structures and are also present across our whole structure and not only here. We modelled some of these blobs (water molecules, co-solvent molecules, etc.) but at this resolution, not everything can be reliably modelled to prevent data overfitting.

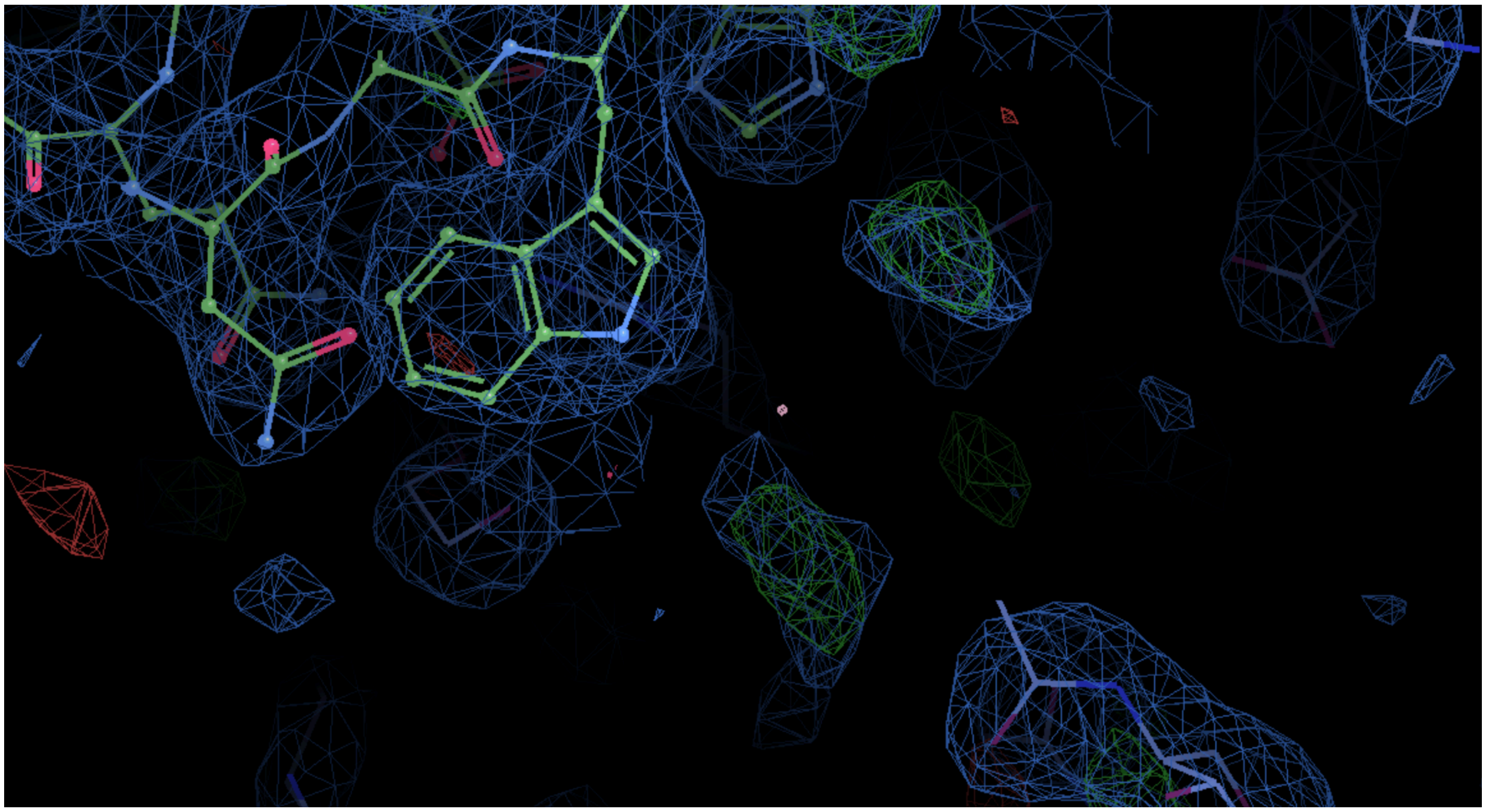

15. Claim of outperforming previous methods
We now qualify this claim and note that additional side-by-side experimental comparisons are shown in the extended data figure 5 and table 1.

16. Extended Data figure formatting
Corrected figure labeling

17. SEC calibration and claims of monodispersity
We have adjusted the wording to clarify that SEC traces suggest monodispersity but do not confirm monomeric status without additional validation. We instead state that we did not not observe significant aggregation during any of our experiments.

18. Extended Data Fig. 7 readability
Secondary structure representations were added to what is now extended data figure 8.

19. Reference formatting
All references have been reformatted to comply with journal standards.

20. Schwarz-Linek et al. (2003) suggestion :
We thank the reviewer for this suggestion and agree that this system could be a compelling future test case for our approach.

REVIEWER COMMENTS

Reviewer #1 (Remarks to the Author):

My concerns and suggestions have been addressed appropriately in the revision. I suggest publication.

Reviewer #2 (Remarks to the Author):

The authors have adequately addressed all of my comments and concerns. Publication is recommended.

Reviewer #3 (Remarks to the Author):

I am satisfied by the response from the authors. They have provided additional information regarding library design and size which clarified their approach and the work described in the paper. The additional experimental data answered my concerns regarding experimental bias and controls.

I only have some minor comments left as detailed below.

1) In extended data table 1, for PDGFRa multiple libraries are mentioned in the paper:
- At p.7: libraries designed against the cryo-EM structure or the AlphaFold 2 model, made of 3780 and 391 members
- In extended data figure 4: "Two binder libraries, β-strand interface conditioned (N=5616) and hotspot only conditioned (N=5717)"
Are those two different experiments and are the numbers listed in extended data table 1 referring to one or both? Could the authors clarify in the table caption.

> **Thank you for your detailed attention to the library sizes, as they were indeed incongruent between the extended data and the main text by mistake. The correct library sizes were indicated in the extended data table, but we neglected to correct the numbers in the text in our last submission. The library size errors result from typos, counting only designs detected sequencing counts from NGS (dropout can be a result of some designs exhibiting poor expression and/or display on yeast cell surfaces), and mistakes made during counting of the library sizes (e.g., the hotspot only conditioned designs were indicated by designs with the patterns 'normal' (N=5727) and 'bcov' (N=4299), which combined yielded the total hotspot-only conditioned library size, N=10026). Both libraries, β-strand interface conditioned (N=5616) and**

hotspot only conditioned (N=10026), were tested in the exact same experiment. We chose to describe them as two different libraries based entirely on the fact that different design methods were used, but libraries were tested in the exact same experiment, simultaneously, as indicated in the extended data figure 4 description.

2) Different library sizes are cited in the paper's main text than listed in data table 1. My understanding is that the numbers in extended data table 1 are before selection criteria were applied. Could the authors explicitly state in the caption if that is the case. While non-essential, adding a column in the table to summarise how many designs were experimentally tested (as stated in the main text for some of the targets) would help with clarity and representation of the importance of the selection criteria.

Every design in each library (all designs counted in extended data table 1) was experimentally tested by yeast surface display. Some library numbers indicated in the manuscript were incorrect due to miscounting (some designs dropping out during initial YSD sorts and therefore were not counted NGS). The library numbers are now consistent, and all reflect the number of DNA sequences ordered and screened. Additionally, we have added to the extended data table 1 description to include the number of designs that were then purified and characterized for solubility and binding.

3) p.4: the first part of the sentence "PDGFRɑ where both helical bundle designs and β-strand pairing designs passed in silico filters, significantly more binders were obtained with the strand directed approach than with standard RFdiffusion at this selection stage (Extended Data Figure 4)." seems incomplete.

Thank you for carefully reading the text. This was rephrased to "For PDGFRɑ, where both helical bundle designs and β-strand pairing designs passed in silico filters, significantly more binders were obtained with the strand directed approach than with standard RFdiffusion at this selection stage (Extended Data Figure 4)." for proper syntax.

4) p.9, typo:
". the There are 21 sidechain-sidechain"

Fixed.

5) p.12, figure 5
"(b-c) FCRL5 is degraded HeLa-FCRL5 cells by de novo binders"
Did the authors mean that "EGFR" is degraded in HeLa-FCRL5?

Yes, that is what we intended. Thank you!

Reviewer #4 (Remarks to the Author):

Largely speaking, the authors have answered our original questions. Some minor remaining points:

Response 11 – should this be Ext Data Fig 4c?

Response 15 – should this be Ext Data Fig 6?

Yes, the figures were renumbered when writing the reviewer responses, but were changed afterward for better organization.

In the section "β-strand interface conditioning improves binder designs", Sappington et al. state that the authors of the original 2023 RFdiffusion paper tested training with SS/ADJ. Sappington et al. now further supplement this by providing an adjacency matrix when running the RFdiffusion run_inference.py script. We think it would be clearer if Sappington et al. more clearly state that in the text this step is performed at inference, possibly in this sentence:

“We explored whether providing interface conditioning information indicating a binder β-strand pairing with a target edge β-strand at the point of inference could yield designable strand pairing complexes.”

We agree that this change will make the text more clear to readers. We replaced the original sentence, “We explored whether providing interface conditioning information indicating a binder β-strand pairing with a target edge β-strand could yield designable strand pairing complexes.” with the suggestion above.

Reviewer #4 (Remarks on code availability):

Conda env installs fine and code works fine in our hands after the following change:

1) Error in line 82 of make_interface_tensor.py
In our hands, the script creates an invalid path for the .txt file that is needed to feed into the RFdiffusion run_inference.py script:

python make_interface_tensor.py --input_pdb alk2_target.pdb --out_dir /local_home/####/NAT_REVIEW/software/ss_adjacency_tensors --binderlen 65 --target_adj B99-103 --binder_ss E --binder_ss_len 6

Error
sh: 1: cannot create
/local_home/####/NAT_REVIEW/software/ss_adjacency_tensors../software.txt:
Directory nonexistent

We were able to fix this by changing line 82 in make_interface_tensor.py from

```
os.system(f'echo {name}_adj{"-".join([str(x[0]) for x in adj_set])}_{counter} >>
{args.out_dir}../{args.out_dir.split("/")[-2]}.txt')
```

to

```
parent_dir = os.path.dirname(os.path.normpath(args.out_dir))
out_name = os.path.basename(os.path.normpath(args.out_dir)) + ".txt"
outfile = os.path.join(parent_dir, out_name)
os.system(f'echo {name}_adj{"-".join([str(x[0]) for x in adj_set])}_{counter} >>
{outfile}')
```

Thank you for taking the time to troubleshoot this on your system and for providing the cleaner code block. We replaced line 82 in the script with the suggested lines.

2) Small change to README file
Need to add ‘python’ before the run_inference.py script commands in the third paragraph

Fixed.

Reviewer #5 (Remarks to the Author):

I co-reviewed this manuscript with one of the reviewers who provided the listed reports. This is part of the Nature Communications initiative to facilitate training in peer review and to provide appropriate recognition for Early Career Researchers who co-review manuscripts.
